# Tailored Mobility in a Zeolite Imidazolate Framework (ZIF) Antibody Conjugate**

**DOI:** 10.1002/chem.202100803

**Published:** 2021-05-21

**Authors:** Ander Chapartegui‐Arias, Anna Raysyan, Ana M. Belenguer, Carsten Jaeger, Teodor Tchipilov, Carsten Prinz, Carlos Abad, Sebastian Beyer, Rudolf J. Schneider, Franziska Emmerling

**Affiliations:** ^1^ BAM Federal Institute for Materials Research and Testing Richard-Willstätter-Str.11 12489 Berlin Germany; ^2^ Department of Chemistry Humboldt-Universität zu Berlin Brook-Taylor-Str. 2 12489 Berlin Germany; ^3^ Yusuf Hamied Department of Chemistry University of Cambridge Lensfield Road Cambridge CB2 1EW United Kingdom; ^4^ Department of Biomedical Engineering Institute for Tissue Engineering and Regenerative Medicine The Chinese University of Hong Kong Shatin Hong Kong; ^5^ Technische Universität Berlin Straße des 17. Juni 135 10623 Berlin Germany; ^6^ SALSA School of Analytical Sciences Adlershof Albert-Einstein-Straße 5 12489 Berlin Germany

**Keywords:** antibodies, immunoassays, metal-organic frameworks, nanoparticles, zeolite analogues

## Abstract

Zeolitic imidazolate framework (ZIF) hybrid fluorescent nanoparticles and ZIF antibody conjugates have been synthesized, characterized, and employed in lateral‐flow immunoassay (LFIA). The bright fluorescence of the conjugates and the possibility to tailor their mobility gives a huge potential for diagnostic assays. An enzyme‐linked immunosorbent assay (ELISA) with horseradish peroxidase (HRP) as label, proved the integrity, stability, and dispersibility of the antibody conjugates, LC‐MS/MS provided evidence that a covalent link was established between these metal‐organic frameworks and lysine residues in IgG antibodies.

## Graphical abstract

**Framing an assay**: We present nanosized zeolitic imidazolate frameworks (ZIFs) conjugated to immunoglobulin G (IgG) antibodies by using a strong and flexible covalent amino‐alkyl link. These nanoZIF‐IgG particles display good mobility and an excellent behaviour in dispersion. Their application to antibody‐based bioanalytical assays, including enzyme‐linked immunosorbent assay (ELISA) and lateral flow immunoassay (LFIA), was examined.

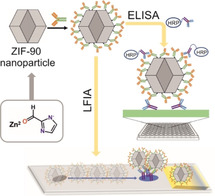

## Introduction

The confrontation with global infections requires the development of robust, sensitive, and selective (bio)analytical tools with good response time. Antibodies can provide the required selectivity in fast analytical approaches. Yet, antibodies, in their natural habitat, are generally protected from external factors, such as ultraviolet radiation, dehydration, and high temperatures. Therefore, the use of antibodies in robust assays requires them to be protected by a pseudo‐skeleton.^[1]^

MOFs have emerged as ideal host materials for a wide range of low molecular weight substances and bio‐macromolecules. MOFs are crystalline porous compounds based on metal ions or clusters connected by organic ligands, which can be designed into many different architectures and tailored for specific uses by adjusting their topology, pore size and/or chemical composition.[^2^, ^3^] In recent years, nanoscale MOFs (nMOFs), are being developed for bio‐applications as they have much larger effective surface areas than traditional MOFs, rendering them improved chemical stability, more efficient surface modification capabilities, and, in consequence, enhanced biological activity of the respective composites.

The particle size of MOFs is determined by the nucleation and growth rates in their synthesis. As a very simplified rule, higher nucleation speed results in smaller particles. Therefore, the synthetic conditions for the preparation of nMOFs must favour nucleation over growth.^[3]^

**Figure 1:**
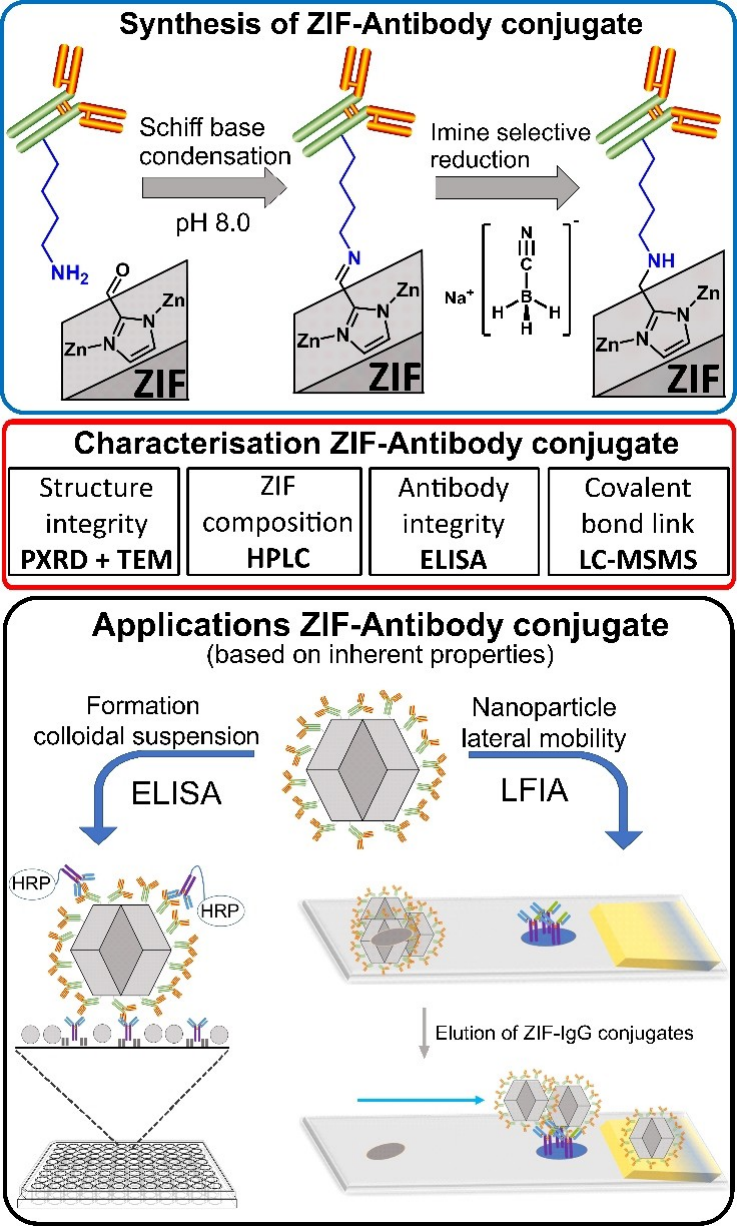
Top: Simplified scheme of the chemistry involved in forming a covalent bond between ZIF‐90 and a mouse IgG antibody resulting in a stable ZIF‐IgG conjugate. Middle: Characterization techniques for ZIF‐antibody conjugates. Bottom: Two applications of the ZIF‐antibody conjugate particles: ELISA and LFIA.

nMOFs have the potential of forming stable colloids in aqueous media essential for use as therapeutic carriers and required for bioanalytical applications. A colloidal system can flow like a liquid and therefore can be used in diagnostic assays like ELISA and LFIA (Figure 1).^[3]^ Despite the great advantages of nMOFs, they suffer from weak resistance to moisture or aqueous environment, owing to the facile decomposition of the metal‐ligand bonds. Metal‐ligand bonds can be significantly strengthened by the use of ligands rich in nitrogen, such as imidazoles, which, on deprotonation, form imidazolate anions.^[4]^ Zeolitic imidazolate frameworks (ZIFs) are a subfamily of MOFs formed by a self‐assembly approach which consists of M‐Im‐M units (with, e. g., M=Zn^2+^, Co^2+^, and Im=imidazolate linkers).^[5]^ Nanoscale ZIFs (nZIFs) display properties combining the advantages of both zeolites and nMOFs, such as ultrahigh surface areas, unimodal nanopores, high crystallinities, abundant functionalities, and exceptional thermal and chemical stabilities^.[5]^ We are most interested in those nZIFs that can form colloids and present large cavities connected through small windows, such as ZIF‐8[^3^, ^6^] and ZIF‐90.^[7]^ ZIF‐8 chemical structure is [Zn(2‐mim)_2_] where 2‐mim is 2‐methylimidazolate and ZIF‐90 is [Zn(i2ca)_2_] where i2ca is imidazolate‐2‐carboxaldehyde. Both ZIFs present a sodalite‐type architecture where Si/Al is replaced with tetrahedral Zn^II^ in ZIFs and O is replaced by their corresponding imidazolate linkers.[^3^, ^7^] ZIF‐8[^3^, ^6^, ^8^] and ZIF‐90^[7]^ have been demonstrated to have permanent porosity, high thermal stability, and remarkable chemical resistance to boiling water, alkaline aqueous solutions, and organic solvents.^[6]^

Cravillon et al. found that excess ligand was essential for promoting faster nucleation and stabilizing the initial colloids in ZIF‐8. The combination of excess ligand, methanol as solvent, and room temperature has since formed the basis for most syntheses of nanoscale ZIF‐8 crystals.[^9^, ^10^] Modulators are used in the synthesis of ZIFs as capping agents. Modulators have two opposing effects on the size of the ZIF particles: i) they can increase the crystal size by competing with the organic ligands, reducing the number of nucleation points, or ii) they can decrease the crystal size by limiting crystal growth. It has been established that reaction time, molar ratio, and reagent concentration are critical parameters in tuning the size and homogeneity of the desired colloidal ZIF‐8 nanocrystals.^[3]^

Yaghi et al. demonstrated that the reactive aldehyde functionalities in ZIF‐90 were able to react with the primary amino group of ethanolamine forming an imine in high yield without significantly altering the original structural integrity.^[7]^

The strategy most predominantly used to date for post‐synthetic modification of MOFs is the treatment of a carboxylic acid moiety with carbodiimide reagents such as 1‐ethyl‐3‐(3‐dimethylaminopropyl)carbodiimide (EDC) to form an activated ester intermediate; this intermediate, on reaction with a primary amine, results in the formation of a covalent amide link between the MOF and the protein/antibody.[^11^, ^12^, ^13^]

The potential of nMOFs conjugated to antibodies as bioanalytical probes has been demonstrated before,[^11^, ^14^] but not for n‐MOFs. Their use bears the potential to expand the application range combining the versatility of structure and functionality of nMOFs with the selectivity and affinity of antibodies.

Common particulate labels for antibodies suffer from poor versatility of conjugation chemistry (as of nanogold) or from a lack of distinct size, morphology, and stoichiometry (such as with frequently used latex particles). We set up clear goals we wanted to achieve when designing the antibody‐conjugated nMOF for proof of concept. 1) We needed the antibody‐conjugated nMOF to have good mobility so that it could be used in ELISA and LFIA tests; 2) it had to retain the crystal structure of the MOF while conserving the binding activity of the antibody; 3) The link between nMOF and antibody had to be relatively long, flexible and robust against cleavage, and 4) the nature of the link should be such, that it could be confirmed by LC‐MS/MS following acid digestion of the antibody‐conjugated nMOF.

We had recently published the synthesis of a luminescent nano ZIF covalently linked to a fluorescent pyrene, referred as Z8P, for the sensitive detection of phthalate plasticizers which can act as endocrine‐disrupting chemicals.^[15]^ The fluorescent nano Z8P has the pyrene fluorophore covalently linked via an imine bond. Luminescent MOF (L‐MOF) sensor materials constraining analytes into the L‐MOF cavity with appropriate pore dimensions can lead to the pre‐concentration of the analytes resulting in high sensitivity and selectivity in fluorescence sensing.^[4]^ The fluorescence probe interaction with analytes like phthalates,^[15]^ or antibodies,[^4^, ^16^] results in competitive absorption, in which the analytes compete with the luminescence probe material for excitation energy leading to apparent emission quenching.

We present here the preparation of a bio‐compatible material based on the conjugation of nano zeolitic imidazolate frameworks (nZIF) with antibodies (Figure 1). We specifically selected ZIF‐90 because of its reactive aldehyde functionalities required to form imine bonds with lysine, this amino acid being prevalent in all antibodies. By selectively reducing the imine bond, a strong and flexible [−CH_2_−NH−CH_2_)_4_−] link is formed. This is the first time that such an antibody ZIF conjugate link is reported. As a proof of concept, the nano‐ZIF‐90 antibody conjugate is used on ELISA and LFIA bioanalytical methodologies.

We decided to investigate if the nano ZIF‐90IgG conjugate could tolerate the additional conjugation to a pyrene fluorophore while maintaining its sodalite type nanostructure, its mobility in aqueous solution, and biological activity without detrimentally affecting the fluorescence activity of the fluorophore.

The motivation for this research was to understand if this ZIF‐90 antibody conjugate with the additional fluorescence tag could offer value to ELISA and LFIA bioanalytical procedures. We hope to prove that the concept of a fluorescent tag, incorporated in a ZIF, conjugated with an antibody, may one day simplify immunoassays. We suggest that it may be feasible to replace the conventional enzyme‐labelled secondary antibody, that turns over a colorimetric substrate, with a simpler, direct fluorescence readout. We also demonstrate that the luminescence tag in a ZIF antibody conjugate can be useful in lateral flow bioanalytical applications.

## Results and Discussion

We selected nZIF‐90, as it is the ideal ZIF for forming a good conjugate with antibodies, based on the properties previously discussed. For the antibody we selected to study immunoglobulin G (IgG) as they are commercially available, inexpensive, and sold as monoclonals of high purity.

A strong and robust linker requires a strong covalent bond between ZIF and the antibody. This could be achieved if we could form an initial imine bond between a lysine and an aldehyde functionality installed on the ZIF, like in ZIF‐90; this imine could later be selectively reduced to −CH_2_−NH− obtaining the desired 6‐membered chain flexible link (Figure 1).

To do so, we first needed to synthesise ZIF‐90 nanoparticles in such a way that they could form a stable colloidal suspension in aqueous media and be suitable for post‐synthetic modification with IgG to form an nZIF‐90 antibody conjugate, referred here as nZIF‐90‐IgG. This synthesis presented an immediate challenge. Conventional modulators are used as capping agents to achieve uniform nanosized ZIFs. The modulator n‐butylamine which had been used previously for the synthesis of nZ8P,^[15]^ would react via a Schiff base condensation with the imidazole‐2‐carboxaldehyde (i2ca) ligand in ZIF‐90. This condensation between n‐butylamine and the i2ca renders the aldehyde functionalities of ZIF‐90 no longer available for post‐synthetic modification with IgG. Instead, sodium methoxide (NaOMe) was found to act as an excellent modulating agent without reacting with the aldehyde functionalities in ZIF‐90.

**Figure 2:**
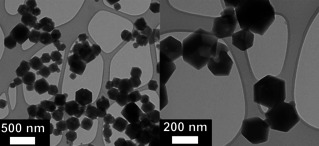
TEM pictures for ZIF‐90. The rhombic dodecahedral shape of ZIF particles^[17]^ can be seen, with some polydispersity in size: between 30 and 350 nm and averaging 150 nm. These sizes were determined from TEM data and are not absolute values.

**Figure 3:**
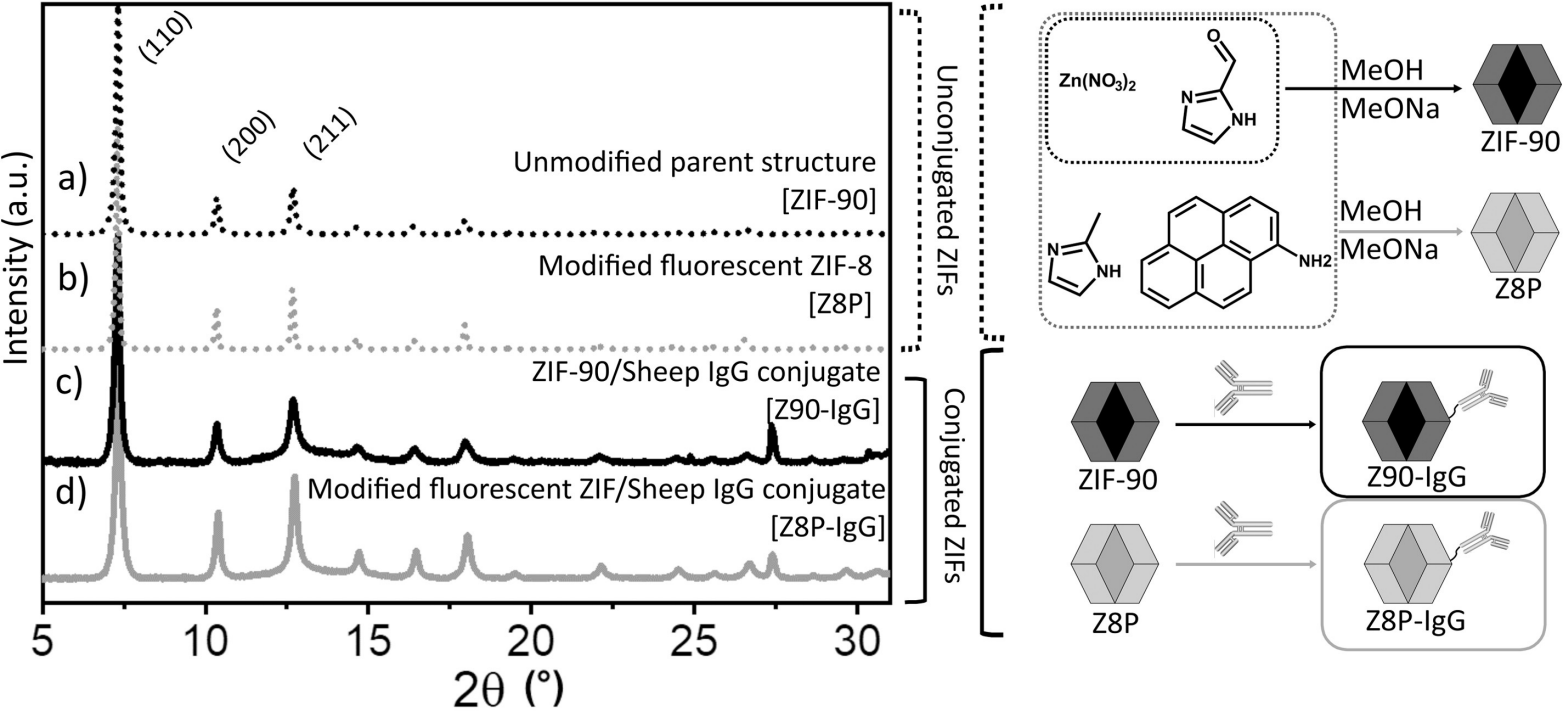
Powder diffractograms for a) nZIF‐90, b) the modified fluorescent ZIF‐8/ZIF‐90 hybrid [nZ8P], c) the nZIF‐90‐IgG conjugate [nZ90‐IgG] and d) the modified fluorescent ZIF‐8/ZIF‐90 hybrid‐IgG conjugate [nZ8P‐IgG].

ZIF‐90 nanoparticles were synthesized in methanol using sodium methoxide as the modulating agent acting as a base to deprotonate the i2ca to the respective imidazolate bridging ligand. The details for the synthesis can be found in the Experimental Section. This synthesis resulted in the formation of non‐aggregated nanoparticles in the size range of 30–350 nm,[^18^, ^19^] as confirmed by transmission electron microscopy (TEM; Figure 2).^[3]^ The corresponding powder X‐ray diffractogram (PXRD) shown in Figure 3a is consistent with a sodalite‐like crystalline structure. This infers that ZIF‐90 has 2 equivalents of i2ca^−^ ligands per one equivalent of Zn^2+^.

The i2ca ligand in the ZIF‐90 increases the hydrophilicity of the ZIF, facilitating its dispersion in aqueous solutions.

As illustrated in Figure 1, we developed a plan to synthesize nZIF‐90‐IgG, set a series of characterisation procedures, and proposed potential bioanalytical applications like ELISA and LFIA for investigation.

In order to synthesise nZIF‐90‐IgG, nZIF‐90 was reacted with IgG, resulting in the formation of an imine via Schiff base conjugation between the ZIF‐90 aldehyde functionality and the (−CH_2_)_4_−NH_2_ pendant amine functionality of lysine in IgG. The imine formed, could be selectively reduced, forming a strong and flexible [−CH_2_−NH−CH_2_)_4_−] link as schematically shown in Figure 1.

Structural characterization of nZIF‐90‐IgG by LC‐MS/MS proved the successful covalent conjugation between the ZIF i2ca moiety and the antibody through this robust link [−CH_2_−NH−CH_2_)_4_−]. To confirm the formation of this strong link, the ZIF conjugates were acid digested (see Section 9.2 in the Supporting Information) in order to cleave all amide bonds of the peptide chains in IgG. At the same time, the acid digestion broke the bonds between Zn and ligands in the ZIF releasing the imidazolate ligands.

**Figure 4:**
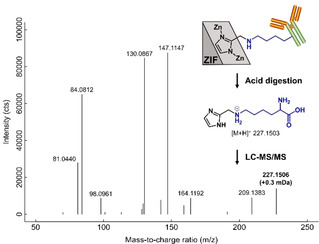
Mass spectrometric evidence of assumed covalent bonding in the ZIF‐90‐IgG conjugate. The MS/MS spectrum shows a putative lysine derivative (precursor *m*/z 227.1503, isolation window 1 Da).

If the antibody was successfully covalently bound through a lysine to ZIF‐90, this cleavage would result in the release of l‐lysines covalently bound via an −NH−CH_2_− to imidazole, as shown in Figure 4. A compound in agreement with this structure was detected in its free form via liquid chromatography high‐resolution mass spectrometry (LC‐HRMS; Figure 4). The compound with a mass‐to‐charge ratio of 227.1503 was present in both ZIF‐IgG conjugates but absent in all experimental controls (individually digested IgG and ZIFs without conjugation). All major MS/MS fragments could be assigned to substructures that explained the putative molecular structure (Section 9.2 in the Supporting Information). Thus, covalent bonding of an imidazole carboxaldehyde moiety to an amino acid residue of the antibody is well supported by the results.

The structural integrity of the nZIF‐90‐IgG is critical to its viability and so is its stability over time. Figure 3c is consistent with nZIF‐90‐IgG having a sodalite‐like crystalline structure proving that the structure of ZIF‐90 is conserved after conjugation with IgG. The calculation of the Scherrer crystallite sizes over time for both conjugates (Section 8.1 in the Supporting Information) shows no change over time on the crystallite sizes for the conjugates for up to 120 hours at 4 °C. This is a strong evidence that the crystal structure of the ZIF part of the ZIF‐IgG conjugates does not suffer significant damage over time.

Dynamic light scattering (DLS) studies in aqueous buffer is used to determine the size of colloidal suspensions. Unlike nanoparticle tracking analysis (NTA), DLS can severely overestimate the particle size of the sample. Larger particles are preferably detected as they scatter much stronger than higher concentrations of smaller particles. The DLS data for Z90‐IgG (Section 8.2 in the Supporting Information) is in the order of 1000–2000 nm, suggesting that Z90‐IgG agglomerates at this high concentration (100 μg/mL borate buffer), and can even precipitate. However, this aggregation does not appear to be detrimental to the storage (120 h at 4 °C) of nZ90‐IgG at concentrations of 200 and 750 μg/mL in borate buffer. This aggregation is also easily reversible by a short sonication burst (3–5 min).

Good mobility of nZIF‐90‐IgG in aqueous solution is critical if these nanoparticles are to be used in bioanalytical applications. The mobility of nZIF‐90‐IgG in aqueous solution relies on the formation of a stable colloidal suspension. These colloidal suspension's mobility is supported by NTA (Sections 8.2.3 and 8.2.4 in the Supporting Information).

NTA, a technique working at very low concentrations in aqueous buffers is used to determine the size of colloidal suspensions. Unlike DLS, it allows small particles to be much more visible than large particles of the same sample as compared with DLS. It provides individual particle sizing, intensity distribution, and approximate concentrations. NTA data for ZIF‐90‐IgG prepared at similar concentrations as typically used for ELISA bioanalytical studies (10 to 20 μg/mL borate buffer), shows a rather broad size distribution with most particles being between 30–350 nm, (Section 8.2.3 in the Supporting Information). This size distribution is consistent with the TEM analysis of nZIF‐90 (Figure2).

To investigate the fluorescence of nZIF antibody conjugate biomaterial, we needed first to synthesise the Z8P nanoparticles. We modified a ZIF‐8/ZIF‐90 hybrid to be fluorescent by including an aminopyrene core. For this, a fluorescent imine compound is initially formed, referred here as “imine”, by reacting 1‐aminopyrene (1‐AP) with i2ca via Schiff base condensation. This *in situ* formed “imine” together with excess of i2ca and 2‐min was reacted by solvothermal procedure using sodium methoxide as the capping agent. NaOMe was used for the same reasons as explained for the synthesis of nZIF‐90. The hybrid ZIF is precipitated obtaining the fluorescently modified ZIF (nZ8P) which contains three imidazolate ligands (i2ca, 2‐mim and the ”imine”). Details for the synthesis can be found in the Experimental Section.

**Figure 5:**
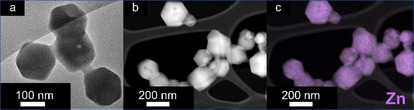
TEM images of nanoparticulate Z8P. a) rhombic dodecahedra characteristic for SOD ZIF systems; b), c) The particles show a polydispersity. c) EDX analysis confirms the presence of Zn (purple).

The synthesis of nZ8P resulted in the formation of nanoparticles in the size range of 50–150 nm,[^18^, ^19^] as confirmed by TEM; (Figure 5).^[3]^ These particles are smaller than those from nZIF‐90 as 2mim is more reactive than i2ca, deprotonating more easily and leading to the formation of smaller nanoparticles. The corresponding PXRD (see Figure 3b) is consistent with a sodalite‐like crystalline structure.

The ratio of ligand‐to‐metal in Z8P must be 2.0 as inferred from the PXRD being consistent with a sodalitetype structure. However, the proportion between the three imidazolate ligands (i2ca, 2‐mim and ”imine”) in Z8P has to be determined by HPLC analysis (Section 5.2 in the Supporting Information) while the concentration of Zn^2+^ in Z8P can be determined indirectly from the HPLC characterization, as well as directly by ICP‐OES analysis (Section 5.3 in the Supporting Information). The HPLC data, supported by the ICP data, confirms a molar ratio of 2.0 between the imidazolates and the Zn^2+^ in Z8P. The authors have previously published, for the first time, the chemical analysis of frameworks by HPLC.[^15^, ^20^]

Notably, the final composition of Z8P by HPLC is consistent with 2‐mim being included on the composition of the hybrid fluorescent ZIF, as required to achieve the desired nanostructure. HPLC analysis supports that most of the imidazolate moieties in Z8P are i2ca which are those that include the aldehyde functionality. This high share of i2ca in Z8P offers suitable binding sites for the antibodies. We can consider Z8P as a ZIF hybrid with approximately 3 : 1 of ZIF‐90 over ZIF‐8 as the imidazolate ligands in ZIF‐90 are exclusively i2ca while those in ZIF‐8 are exclusively 2‐mim.

As in the case of ZIF‐90 previously discussed, i2ca in Z8P increases the hydrophilicity of the ZIF, facilitating its dispersion in aqueous solutions.

The same post‐synthetic methodology depicted in Figure 1 is used to prepare nZ8P‐IgG. The free aldehyde functionalities of nZ8P are reacted with IgG, resulting in the formation of a Schiff base. The selective reduction of the imine bond resulted in the formation of a strong and flexible link [−CH_2_−NH−CH_2_)_4_−] between the Z8P and the antibody. The sodalite structure from nZ8P is being retained upon the formation of nZ8P‐IgG as supported by PXRD (Figure 3d). The particle size of nZ8P although retained in nZ8P‐IgG (Figure S29 in the Supporting Information), shows agglomeration as it is prepared at high concentrations and in MeOH, a poor dispersant. The crystal structures and corresponding particle sizes of nZ8P‐IgG remain intact for up to 120 hours at 4 °C based on PXRD investigations (Section 8.1 in the Supporting Information), as previously discussed for nZIF‐90‐IgG.

We have demonstrated here that both nZIF‐90‐IgG and nZ8P‐IgG conserve their sodalite‐like crystalline structure after conjugation. Their nanosized particle range allows them to form stable colloidal suspensions in aqueous media as supported by NTA and DLS experiments.

The first important property to be conserved during conjugation of ZIFs to antibodies is the binding properties of the conjugated IgGs in terms of affinity and selectivity. The second important property of the conjugates is that they should be highly mobile in dispersion (for ELISA applications) as well as mobile in lateral flow on a solid matrix (for LFIA applications, e. g. on polyvinylidene difluoride (PVDF) membranes).

**Figure 6:**
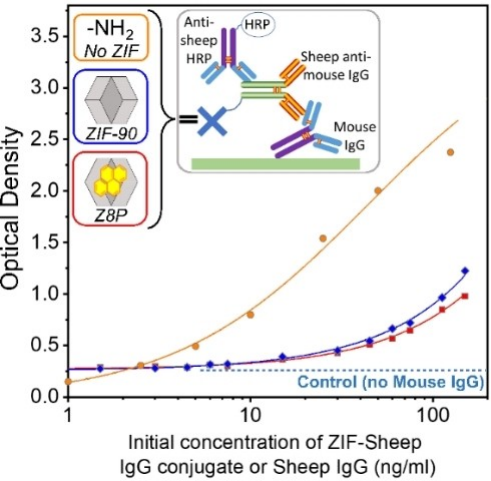
Proving the integrity of the ZIF‐IgG conjugate: Results from a noncompetitive ELISA. Unmodified anti‐mouse IgG (from sheep) used as reference (•), Z8P‐IgG (▪) and Z90‐IgG (⧫). HRP: horseradish peroxidase.

In a conventional indirect non‐competitive ELISA, the binding of the antibody is quantified by adding an enzyme‐labelled secondary antibody that turns over a colorimetric substrate. Employing nZIF‐90‐IgG and nZ8P‐IgG in this model, ELISA resulted in the expected sigmoidal curves (Figure 6). This proves that the antibodies in nZ90‐IgG and nZ8P‐IgG retained their binding properties. The successful application of nZIF‐90‐IgG to ELISA is also a strong pointer towards their mobility; the reproducibility on each replicate requires to be low for good mobility (Figure S20 in the Supporting Information, error bars for Z8P‐IgG and Z90‐IgG).

An important feature to be retained in nZ8P‐IgG, is that its fluorescence must be conserved in the presence of the conjugate antibody, so that these biomaterials could be employed in fluorescence immunoassays and/or fluorescence quenching assays. We tested the response of nZ8P‐IgG against various concentrations of dimethyl phthalate (DMP) in order to check if fluorescence quenching was conserved in comparison to the unconjugated Z8P (Section 5.1 in the Supporting Information). The presence of antibodies around the Z8P nanoparticles proved not to interfere with this mechanism, nZ8P‐IgG was still sensitive to quenching by short‐chained phthalates; the fluorescence intensity as a function of the concentration of dimethyl phthalate was determined. A response comparable to the one reported previously by us for this system was recorded (Figure S10 in the Supporting Information).^[15]^

To turn the enzyme immunoassay into a fluorescence immunoassay based on the inherent fluorescent properties of Z8P, the model non‐competitive indirect ELISA was performed at higher concentrations (Sections 7.4.1 and 7.4.2 in the Supporting Information).

**Figure 7:**
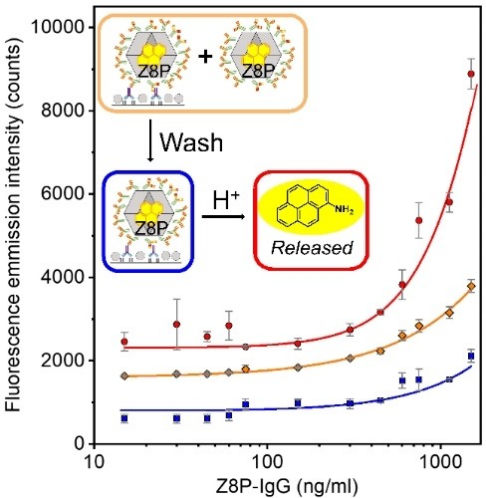
Fluorescence emission for the noncompetitive indirect assay employing Z8P‐IgG (*λ*_ex_=335 nm). Prewash fluorescence with excess nZ8P‐IgG (⧫), post wash (▪), and released aminopyrene fluorophore (•) from acid hydrolysis of the imine.

Fluorescence measurements (*λ*_ex_=335 nm) on this system resulted in low fluorescence emission intensities. After a washing step to remove loosely bound Z8P particles, the assay gave even lower intensities (Figure 7). This drop in fluorescence intensity is partly due to all the non‐conjugated Z8P particles being removed. Only those Z8P which were conjugated to antibodies remained retained on the surface of the microtiter plate (MTP). After the addition of an acidic buffer, that protonates the imidazolate buinding blocks and breaks down the ZIFs, a significant increase in fluorescence can be seen. We believe this to be evidence of the inner‐filter quenching^[21]^ that is happening on the Z8P ZIF nanoparticles being eliminated once the ZIF particles were broken dowin to its constituents. This inner filter effect has also been reported for other nanocarrier systems such as liposomes.^[22]^

As for the nature of the quenching itself, when considering the characteristics of the absorption spectra for DMPs^[23]^ and for the imidazolate species forming the modified ZIF (UV absorption spectra, Figure S11 in the Supporting Information) suggests that they do not absorb at the wavelengths involved in the fluorescence spectra (excitation at 288 nm and emission of the imine within the Z8P ZIF between 400 and 540 nm (Figure S10a in the Supporting Information)). Therefore, it must be assumed that the quenching is not due to quenching effects by absorption of the emitted radiation by the imine. Instead, we believe a π‐ π stacking that leads to the formation of an exciton state, does no longer absorb radiation. This excimer could be formed in the bulk of the Z8P particles, since DMP could diffuse through the pores of the ZIF and become trapped in the cavity with the pyrene moiety part of the imines.

The effect that the antibodies exert on the Z8P particles’ surface is more difficult for us to say. It is likely that the antibodies can absorb part of the incident excitation radiation, as well as quenching the fluorophores on the surface via excimer formation (some amino acids have aromatic moieties). A more detailed explanation can be found in Section 5.1 in the Supporting Information.

We appreciate that aminopyrene is not optimal for fluorescence detection because the MTP material used in ELISA also absorbs light in the UV range. As Z8P‐IgG was only investigated as a proof of concept, we did not search for an improved fluorescence tag, as the optimisation of this fluorophore was outside our objectives.

Z8P‐IgG was tested for its suitability for applications in LFIA. To facilitate this LFIA test and to eliminate the need of one additional antibody carrying the HRP probe, another Z8P‐IgG conjugate, nZ8P‐IgG‐HRP was synthesised. Details for the synthesis can be found in the Experimental Section. We employed a commercially available anti‐sheep IgG‐HRP conjugate. The synthesis of nZ8P‐IgG‐HRP is analogous to nZ8P‐IgG (Section 10.1 in the Supporting Information).

**Figure 8:**
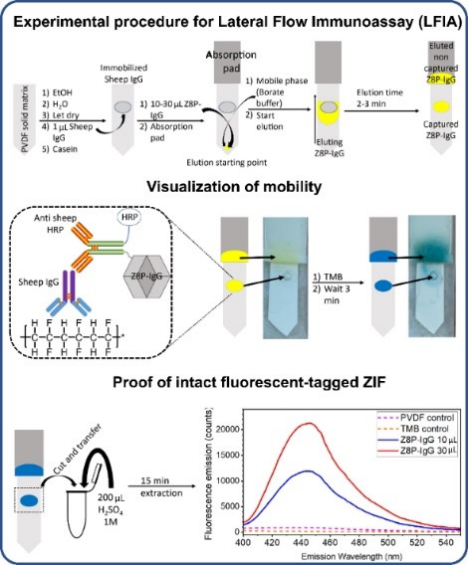
Experimental setup to demonstrate the aptitude of Z8P‐IgG for application in lateral flow immunoassays (LFIA). Top row: Assay protocol. Middle row: Visualization of the mobility of the ZIF composite and retention of binding specificity of the antibody: Bottom row: Proof of intact ZIF antibody conjugate. TMB: 3,3’,5,5’‐tetramethylbenzidine, HRP: horseradish peroxidase, PVDF: polyvinylidene difluoride.

The only significant advantage of using Z8P‐IgG over Z90‐IgG is for the validation of the LFIA bioanalytical assay. The emission of the fluorescence pyrene tag at the antibody capture zone demonstrates that IgG must travel as a conjugate with the ZIF. To prove so, this zone was cut and digested with sulphuric acid, to release the fluorophore 1‐aminopyrene. Dependent on the amount of Z8P‐IgG employed (and thus captured), fluorescence was observed (Figure 8).

Aggregation as demonstrated by DLS is also not detrimental to the mobility of nZ8P‐IgG across the PVDF solid matrix at high particle concentrations (750 μg/mL borate buffer). The mobility of nZ8P‐IgG is only stopped at the antibody capture zone. During the LFIA elution of the Z8P‐IgG dispersion, the presence of the antibody conjugate in the capture zone is detected by the HRP colour change as well as the presence of the nZ8P is proven by the fluorescence of its pyrene conjugate.

## Conclusion

This is the first time that the formation of a stable covalent linkage of an antibody conjugated to a zeolite imidazolate framework (ZIF) has been demonstrated. These ZIF‐antibody conjugates have been extensively characterised, demonstrating that all the properties of its constituent parts, ZIFs and immunoglobulins, are retained. In addition to preserving the mobility of the nanoparticulate entity, the conjugation also prevents protein aggregation of the antibodies at low aqueous concentrations and, like an exoskeleton, might elicit protection against degradation. These ZIF antibody conjugates with tailored mobility drastically expand the potential of ZIFs to be applied as highly selective bioprobes in a variety of analytical and diagnostic applications, such as enzyme‐linked immunosorbent assay (ELISA) and lateral flow immunoassay (LFIA). Our findings should be of interest in the development of better portable formats, a key aspect gaining importance in the scientific confrontation with global diseases and pandemics.

## Experimental Section

**Preparation of the 1‐aminopyrene‐functionalized nanoparticulated ZIF‐8/ZIF‐90 hybrid (Z8P)**: 16.08 mg (0.075 mmol) of 1‐aminopyrene (1‐AP) and 47.28 mg (0.493 mmol) of imidazolate‐2‐carboxaldehyde (i2ca) were added to a sealable glass flask, 5 mL of methanol was added, the glass flask was sealed with an aluminium cap and heated at 80 °C for 30 min with stirring. After cooling to ambient temperature, 40.4 mg (0.493 mmol) of 2‐methylimidazole (2‐mim) and 250 μL of a nearly saturated solution of MeONa in methanol (0.25 g/mL) were added. 5 ml of a Zn(NO_3_)_2_ ⋅ 6⋅H_2_O solution (146 mg of Zn(NO_3_)_2_ ⋅ 6⋅H_2_O (0.494 mmol) in 10 mL of methanol) was added. A yellow precipitate appeared which was left for 4 h undisturbed, and later taken through an extensive washing procedure to remove unbound compounds forming ZIF‐8/ZIF‐90 hybrid (Z8P). For more in‐depth information see Section 4.1 in the Supporting Information.

**Preparation of nanoparticulated nZIF‐90‐IgG**: 1.5 mL of a borate buffer solution was added to 1.5 mg of the ZIF‐90, the vial was sealed and sonicated. 50 μL of a 2 mg/mL solution of sheep anti‐mouse IgG was added. 50 μL of Na[BH_3_(CN)] (5 M in 1 M NaOH) was added and allowed to react for 4 h at RT. For more in‐depth information see Section 6.1 in the Supporting Information. ZIF‐8P‐IgG is used for ELISA bioanalysis.

***CAUTION***! Na[BH_3_(CN)] is volatile and extremely toxic when inhaled or in contact with the skin.

**Preparation of nanoparticulated Z8P‐IgG‐HRP**: 1.5 mL of a borate buffer solution was added to 1.5 mg of the Z8P, the vial was sealed and sonicate. 50 μL of a 1.8 mg/mL solution of anti‐sheep IgG‐HRP was added. 50 μL of Na[BH_3_(CN)] (5 M in 1 M NaOH) was added and allowed to react for 4 h at RT. For more in‐depth information see Section 10.1 in the Supporting Information. Z8P‐IgG‐HRP is used for LFIA bioanalysis.

## Conflict of interest

The authors declare no conflict of interest.

## Supporting information

As a service to our authors and readers, this journal provides supporting information supplied by the authors. Such materials are peer reviewed and may be re‐organized for online delivery, but are not copy‐edited or typeset. Technical support issues arising from supporting information (other than missing files) should be addressed to the authors.

Supplementary
