# Supplementary material for: Tailored Mobility in a Zeolite Imidazolate Framework (ZIF) Antibody Conjugate
Source: Chemistry. 2021 May 21;27(36):9414–21. doi: 10.1002/chem.202100803 (PMC8362128; doi:10.1002/chem.202100803)
Supplement: Supplementary file 1 — Supplementary [file CHEM-27-9414-s001.pdf]

Chemistry–A European Journal

Supporting Information

Tailored Mobility in a Zeolite Imidazolate Framework (ZIF) Antibody Conjugate**

Ander Chapartegui-Arias+, Anna Raysyan+, Ana M. Belenguer,* Carsten Jaeger, Teodor Tchipilov, Carsten Prinz, Carlos Abad, Sebastian Beyer, Rudolf J. Schneider,* and Franziska Emmerling*

Nomenclature and abbreviations used

Abbreviation	Meaning
1-AP	1-Aminopyrene
2-mim	2-Methylimidazolate
CBZ	Carbamazepine
DMP	Dimethyl phthalate
DMSO	Dimethylsulphoxide
EDS	Energy-dispersive x-ray spectroscopy
ELISA	enzyme-linked immunosorbent assay
FTIR	Fourier-transform infrared spectroscopy
HPLC	High-performance liquid chromatography
HRP	Horseradish peroxidase
DLS	Dynamic Lase Scattering
NTA	Nanoparticle Tracking
i2ca	Imidazol-2-carboxaldehyde when referring to neutral species or [illegible]
ICP-OES	Inductively coupled plasma atomic emission spectroscopy
IgG	Immunoglobulin type G
MeONa	Sodium methoxide
Na_2HPO_4	Disodium hydrogen phosphate
$NaBH_3CN$	Sodium cyanoborohydride
NaH_2PO_4	Sodium phosphate monobasic
OD	Optical density
PBS (buffer)	Phosphate-buffered saline solution
PFA	Perfluoroalkoxy alkane
PVDF	Polyvinylidene fluoride
PXRD	Powder x-ray diffractometry
Sheep IgG	Immunoglobulin type G from sheep
S-TEM	Scanning transmission electron microscopy
TEM	Transmission electron microscopy
TMB	3,3’,5,5’-tetramethylbenzidine
TRIS (buffer)	tris(hydroxymethyl)aminomethane
Z8P	Pyrene-modified ZIF-8/ZIF-90 hybrid
Z8P–IgG	Z8P IgG antibody conjugate
Z90–IgG	ZIF-90 IgG antibody conjugate

Table of Contents

1 Chemical and Reagents 5
1.1 Reagents for ZIF synthesis 5
1.2 Reagents for HPLC 5
1.3 Reagents for ICP-OES 5
1.4 Reagents for LC/MS-MS 5
1.5 Reagents for buffers 5
1.6 Antibodies 6
1.7 Solvent for NMR 6
2 Preparation of buffers 6
2.1 Preparation of the phosphate buffer for HPLC (pH 8.8) 6
2.2 Preparation of the buffers for immunoanalysis 6
2.2.1 Borate buffer 6
2.2.2 Phosphate buffered saline (PBS) 6
2.2.3 TRIS buffer 7
2.2.4 Citrate buffer 7
2.2.5 Wash buffer 7
2.2.6 TMB solution 7
3 Analytical Investigations: Instruments and Methods 7
3.1 FTIR: 7
3.2 TEM: 7
3.3 NMR: 8
3.4 Bragg-Brentano diffractometry: 8
3.5 Fluorescence spectrometry: 8
3.6 HPLC: 8
3.6.1 HPLC method 8
3.7 ICP-OES: 9
3.8 Absorbance spectrometry: 9
3.9 LC-MS: 9
3.9.1 LCMS method 9
3.10 DLS measurements: 9
3.11 Nanoparticle tracking: 9
4 Synthesis and characterisation of ZIFs 10

4.1 Preparation of the 1-aminopyrene-functionalized nanoparticulated ZIF-8/ZIF-90 hybrid (Z8P) 10

4.2 Preparation of the nanoparticulated ZIF-90 11

4.3 Preparation of ZIF-8 11

4.4 Structural characterization of ZIFs 12

4.4.1 FTIR characterization of the synthesised ZIFs 12

4.4.2 Characterization of the ZIF particles and conjugate particles via TEM......... 13

4.5 Synthesis of the imine formed by Schiff condensation of 1-AP and i2ca 14

4.6 Characterisation of 1-AP, i2ca and the imine formed by Schiff condensation of 1-AP and i2ca 15

4.6.1 Characterization by 1H NMR 15

4.6.2 Characterization of imine by PXRD 19

5 Additional analysis and characterisation of ZIF and ZIF conjugates 20

5.1 Fluorescence characterization the conjugated Z8P particles versus DMP 20

5.2 HPLC procedure to obtain the chemical composition of ligands in Z8P 22

5.2.1 Preparation of Standard solutions 25

5.2.2 Validation of the HPLC method: Linearity 26

5.2.3 Preparation of Z8P acid digestion sample solutions 27

5.3 Quantification of Zn^{2+} content on Z8P by ICP-OES 31

5.3.1 Suitable disintegration procedure for Z8P analyses by ICP-OES................. 31

5.3.2 ICP-OES parameter for analysis of disintegrated Z8P 31

5.3.3 ICP Determination of Zn^{2+}; Results... 32

5.3.4 Summary of HPLC and ICP-OES results.. 32

5.4 Powder X-Ray diffractograms for the ZIFs, both conjugated and unconjugated 34

5.4.1 Preparation of PXRD sample of non-conjugated ZIFs................................. 34

5.4.2 Preparation of PXRD sample of conjugated ZIFs.. 34

6 Conjugation of ZIFs with sheep IgG 36

6.1 General procedure scheme.. 36

7 Studies on conjugated antibody activity 38

7.1 Model non-competitive indirect ELISA 38

7.2 Protocol of the non-competitive indirect ELISA for the anti-sheep IgG primary . 39

7.2.1 Preparation of Capture antibody solution 40

7.2.2 Preparation of Casein blocking solution 40

7.2.3 Preparation of sheep anti-mouse IgG solutions in borate buffer................... 41

7.2.4 Preparation of the solution of HRP-labelled detection antibody 42

7.2.5 Preparation of the solution of TMB-based substrate 42

7.3 Protocol of the non-competitive indirect ELISA for the ZIF-IgG conjugates 44
7.3.1 Preparation of ZIF−IgG solutions in borate buffer: 45
7.4 Protocol of the non-competitive ELISA for the antibody conjugates Z8P-IgG / non-competitive fluorescent ELISA tandem 49
7.4.1 Protocol of the non-competitive ELISA for the antibody conjugates Z8P-IgG 49
7.4.2 Protocol of the non-competitive ELISA for the antibody conjugates Z8P-IgG via non-competitive fluorescence immunoassay 52
8 Studies on the decomposition of the ZIF conjugates 54
8.1 PXRD measurements of the Z90-IgG and Z8P-IgG conjugates 54
8.2 Dynamic laser scattering (DLS) of the Z90-IgG conjugates 58
8.2.1 Dispersant phase preparation 58
8.2.2 DLS sample preparation and results 58
8.2.3 Nanoparticle tracking measurements for Z90-IgG. 61
8.2.4 NTA sample preparation and results 61
9 Studies on the ZIF-antibody bond 62
9.1 TEM images of the ZIF-IgG conjugates 62
9.2 Study of the nature of the bond on the ZIF-IgG conjugates via HRMS 63
9.2.1 Digestion of the conjugate 64
9.2.2 High-resolution mass spectrometry 65
10 Studies on the mobility of the ZIF-antibody conjugate 72
10.1 Conjugation reaction of Z8P particles to IgG−HRP 72
10.2 Mobility studies of ZIF conjugates on a PVDF membrane 73
11 References 76

1 Chemical and Reagents

1.1 Reagents for ZIF synthesis

Zinc nitrate hexahydrate (CAS: 10196-18-6) was purchased from Roth (100% purity); 2-methylimidazole (CAS: 693-98-1) was purchased from Acros Organics (100% purity); 1-Aminopyrene (CAS: 1606-67-3; 95% purity) and Imidazolate-2-carboxaldehyde (CAS: 10111-08-7; 97% purity) were purchased from Sigma-Aldrich. Sodium methoxide (CAS: 124-41-4; 95% purity) was purchased from Merck. Sodium cyanoborohydride solution (CAS: 25895-60-7) 5.0 M in 1 M NaOH was purchased from Sigma-Aldrich. Methanol and dimethylformamide HPLC grade where purchased at CHEMSOLUTE.

1.2 Reagents for HPLC

All HPLC solvents used were of high purity HPLC grade. Water was acquired from a Milli-Q® Direct Water Purification System [18.2 MΩ•cm (25°C)]. Methanol for HPLC was purchased from CHEMSOLUTE as HPLC grade and TFA was purchased from Sigma Aldrich as Reagent-Plus 99 %.

- Na_2HPO_4 (Reagent grade, CAS: 10028-24-7)
- NaH_2PO_4 (Reagent grade, CAS: 10049-21-5)

1.3 Reagents for ICP-OES

HNO_3 65% (grade for analysis EMSURE® Reag. PhEur, ISO, Merck)

Ultrapure deionized water (18.2MΩ cm−1, 25°C) was used for dilutions (Milli-Q Gradient, Merck Millipore).

The external standard solution of a Zn is directly traceable to the NIST SRM 3168a (Certipur® Certified Reference Material, $Zn(NO_3)_2$ in HNO_3 2-3% Suprapur® Merck, Germany).

1.4 Reagents for LC/MS-MS

All solvents and additives (acetonitrile, ammonium formate, formic acid) were of LC-MS grade (CHEMSOLUTE,).

Water was obtained from an ultrapure water purification system (Elga).

1.5 Reagents for buffers

All reagents for buffer preparation, and casein, were purchased from Merck.

- Di-sodium tetraborate decahydrate (Ph Eur,BP,JP,NF grade, CAS: 1303-96-4)
- Sodium chloride (Reagent grade, CAS: 7647-14-5)
- Sodium azide solution (10% w/v in water, CAS: 26628-22-8)
- $NaH_2PO_4 \cdot 2H_2O$ (Reag. Ph Eur grade, CAS: 10049-21-5)
- $Na_2HPO_4 \cdot 2H_2O$ (Ph Eur,BP,USP grade, CAS: 10028-24-7)
- $C_4H_{11}NO_3$ (TRIS, Ph Eur grade, CAS: 77-86-1)
- Sodium dihydrogen citrate (Reagent grade, CAS: 18996-35-5)
- KH_2PO_4 (molecular biology grade, CAS: 7778-77-0)
- K_2HPO_4 (Analysis grade, CAS: 7758-11-4)
- $C_6H_7KO_2$ (potassium sorbate, Ph Eur,BP,NF,FCC,E 202 grade, CAS: 24634-61-5)
- 0.05% (v/v) Tween™ 20 solution (CAS: 9005-64-5)

- Tetrabutylammonium borohydride (synthesis grade, CAS: 33725-74-5)
- 3,3′,5,5′-tetramethylbenzidine (TMB, analysis grade, CAS: 54827-17-7)
- Casein (technical grade, CAS: 9000-71-9)

1.6 Antibodies

HRP-conjugated donkey anti-sheep-IgG (A3415), 1.8 mg/mL, in buffer (50 mM Na_2HPO_4, 20 mM Na_2HPO_4, TRIS HCl 0.1 M, pH 7.4, azide 0.02% w/v), was purchased from Sigma-Aldrich.

Anti-mouse IgG from sheep (R1256P), 2 mg/ml, in PBS buffer, was purchased from Acris Antibody GmbH (Herford, Germany).

Mouse anti-CBZ IgG (AK2512/01), 1.75 mg/mL, in PBS buffer, ordering code BAM-mab 01 (CBZ) was obtained from BAM.[1]

1.7 Solvent for NMR

d6-DMSO (CAS: 2206-27-1, 99.9%) in 0.75 ml ampules was purchased from Sigma-Aldrich

2 Preparation of buffers

2.1 Preparation of the phosphate buffer for HPLC (pH 8.8)

1.75 g of Na_2HPO_4 (9.83 mmol) and 16.29 mg of NaH_2PO_4 (0.12 mmol) were weighed using a 5-figure balance, transferred to a 1 L HPLC glass solvent bottle and added 1 L of water obtained from a Milli-Q® Direct Water Purification System [18.2 MΩ•cm (25°C)], measuring the added volume with a 1 L graduated glass cylinder. After complete dissolution, the buffer was filtered through a 0.45 µm nylon membrane (47 mm diameter) using a 1 L solvent vacuum microfiltration apparatus to remove any undissolved crystal. The pH was determined to be 8.60 using a pH meter, and the buffer was ready to use.

2.2 Preparation of the buffers for immunoanalysis

2.2.1 Borate buffer

9.64 g (48 mmol) of di-sodium tetraborate decahydrate and 8.29 g (142 mmol) of sodium chloride were weighed and transferred to a 1 L HPLC glass solvent bottle. Using a 1L graduated glass cylinder 1 L of ultrapure water, obtained from a Milli-Q® Direct Water Purification System [18.2 MΩ•cm (25°C)], was added to the bottle. After full dissolution 1 mL of a sodium azide solution (10% w/v in water) was added and mixed. The final pH of the buffer was measured and determined to be pH 9.05.

2.2.2 Phosphate buffered saline (PBS)

1.56 g (10 mmol) of $NaH_2PO_4{\cdot}2H_2O$, 12.46 g of $Na_2HPO_4{\cdot}2H_2O$ (70 mmol) and 8.47 g of NaCl (142 mmol) were weighed, transferred to a 1 L HPLC glass solvent bottle, 1 L of ultrapure water was added and the pH determined to be pH 7.60.

2.2.3 TRIS buffer

1.21 g of $C_4H_{11}NO_3$ (TRIS, 10 mmol) and 8.77 g of NaCl (150 mmol) were weighed and, as described in SI-2.2.2, used to prepare 1 L of buffer, pH 8.50.

2.2.4 Citrate buffer

47.10 g of sodium dihydrogen citrate (220 mmol) were weighed and, as described in SI-2.2.2, used to prepare 1 L of buffer, pH 4.00. For reasons of stability, the buffer was kept in the fridge at 4°C.

2.2.5 Wash buffer

6.124 g of KH_2PO_4 (45 mmol), 65.32 g of K_2HPO_4 (375 mmol), 0.225 g of $C_6H_7KO_2$ (potassium sorbate, 1.5 mmol) were weighed and, as described in SI-2.2.2, used to prepare 1 L of buffer. 30 ml of a 0.05% (v/v) Tween™ 20 solution using a 100 mL graduated glass cylinder was added. The final pH of the buffer was measured and determined to be pH 7.60.

2.2.6 TMB solution

102.93 mg of tetrabutylammonium borohydride (8 mmol) and 480.7 mg of 3,3′,5,5′-tetramethylbenzidine (TMB, 40 mmol) were weighed using a 5-figure analytical balance, transferred to a 200 mL new glass bottle and 50 mL of Milli-Q water added. For stability, the buffer was kept in the fridge at 4°C.

3 Analytical Investigations: Instruments and Methods

3.1 FTIR:

Fourier-transform infrared spectra were recorded on a Nicolet FT-IR NEXUS instrument with a Golden Gate-Diamond-ATR Unit and a DTGS KBr detector. For each measurement 32 sample scans and 32 background scans were recorded. The spectral range was from 200 cm^{-1} to 4000 cm^{-1} with a resolution of 4 cm^{-1}.

3.2 TEM:

Transmission electron microscopy (TEM) images were obtained in a Talos F200S Microscope (Thermo Fisher Scientific) by using a 200 kV microscopy technique in which a beam of electrons is transmitted through a specimen to form an image. A Ceta 16M camera (TEM mode) and a HAADF detector (STEM mode) were used to capture the images.

Additionally, a side-entry retractable Energy Dispersive X-ray Spectroscopy (EDS) detector were also used in the STEM mode to collect detailed elemental information.

The specimens were prepared by dropping sample solutions onto a 3 mm copper grid (Lacey Carbon, 400 mesh) and leaving them to air-dry at room temperature.

3.3 NMR:

Nuclear magnetic resonance measurements were performed on a 400 MHz NMR spectrometer (Mercury-400 BB) at 29.0 °C, with a relaxation delay of 2.000 sec, a pulse of 45.0°, an acquisition time of 2.274 sec and 16 repetitions. Solvent was deuterated DMSO.

3.4 Bragg-Brentano diffractometry:

Powder X-ray diffractometry (PXRD) patterns were collected with Cu Kα radiation (λ = 1.50406 nm) on a D8 Advanced diffractometer (Bruker AXS, Germany) equipped with a LYNXEYE detector. Samples were measured in reflection geometry in a 2Θ range from 4° to 50° with a step size of 0.009° with spinning setup.

3.5 Fluorescence spectrometry:

Fluorescence spectra were obtained on an all-in-one microplate reader Synergy H1 with dispenser (BioTek). The software used was Gen5. The spectra was acquired from 450 to 600 nm, with a step of 2 nm, measurement time of 10 ms for the particle characterization, and of 50 ms for the fluorescence ELISA, with delay between measurements of 100 ms. The excitation wavelength was 335 nm. The samples were handled in non-binding or high-binding polypropylene 96-well black microtiter plates from Greiner Bio-One.

3.6 HPLC:

High performance liquid chromatography analysis of the Z8P samples were performed using a modular Agilent 1100 Series HPLC system composed of a high pressure binary pump, auto-sampler with injector programming capabilities, Peltier type column oven with 6 µL heat exchanger and a Diode Array Detector with a micro flow cell (1.6uL, 6mm pathlength) to reduce peak dispersion when using short columns as in this case. The flow-path was connected using 0.12 mm ID stainless steel tubing to minimize peak dispersion. ChemStation software was used to process the HPLC data.

3.6.1 HPLC method

HPLC column was Excel C18-PFP with 2 µm particle size (4.6 mm internal diameter × 50 mm length) manufactured by ACE. The C18-PFP stationary phase was prepared by covalently bonding a pentafluorophenyl octadecylsilane to pure silica. The HPLC column is manufactured to be stable up to pH 10 and 1000 bar back pressure.

HPLC conditions:

Solvent A = Phosphate buffer 10 mmol, pH=8.8
Solvent B = MeOH
0-4 min; 10-95 % B; run time of 7 min; post time of 2 min.
Flow rate was 2 mL/min; column temperature of 45 °C; 1 µL injection.

The chosen wavelength for 2-methylimidazole was 210 nm (8 nm bandwidth), for imidazolate-2-carboxaldehyde 280 nm (8 nm bandwidth) and for 1-aminopyrene 338 nm (8 nm bandwidth), all having a reference of 550 nm (100 nm bandwidth, Fig. S11).

3.7 ICP-OES:

For inductively coupled plasma optical emission spectrometry an Agilent 5110 VDV ICP-OES instrument was employed (Agilent Technologies, USA). The emission line of zinc at wavelength 202.548 nm at radial mode was used. Detailed operating conditions are listed in Table S8.

3.8 Absorbance spectrometry:

Absorbance, with measurements in microtiter plates usually designated as optical density (OD), was measured on a SpectraMax Plus384 microplate reader (Molecular Devices, Ismaning, Germany) at 450 nm, referenced to 620 nm. All calibration standard solutions were determined in triplicate. SoftMax®Pro Software (v.5.3, Molecular Devices) was used for data acquisition

3.9 LC-MS:

Liquid chromatography-mass spectrometry analysis was performed on a 1290 UHPLC system (Agilent, Germany) coupled to a 6600 TripleTOF mass spectrometer (SCIEX, Germany).

3.9.1 LCMS method

Samples were separated on a 2.1 mm × 75 mm × 1.8 µm BEH amide column (Waters, Germany) using a hydrophilic liquid interaction chromatography (HILIC) mode.

Solvent A: acetonitrile/water 50:50, 10 mM ammonium formate, 0.125% formic acid.

Solvent B: acetonitrile/water 95:5, 10 mM ammonium formate, 0.125% formic acid.

Solvent gradient (%B): 0 min 100%, 1 min 100%, 10 min 0%, 12 min 0%, 12.1 min 100%, 15 min 100%.

Flow rate: 0.5 mL/min. Column temperature: 35 °C.

Positive and negative electrospray ionization (ESI+/-) was carried out at 320 °C using capillary voltages of 5500 V and 4500 V, respectively. Mass spectrometric detection was performed in full-scan mode (m/z 50-600) at a scan rate of 4 s^{-1} using data-dependent MS/MS acquisition (DDA Top-4). For data analysis, raw data files were converted to mzML format and imported into an R workspace. Peak pre-processing was performed using package xcms[2]. For MS/MS fragment substructure annotation, competitive fragmentation modelling was used[3] (CFM-ID).

3.10 DLS measurements:

The dynamic light scattering measurements were performed on a Zetasizer Nano ZS, Malvern Instruments, UK) equipped with a He-Ne laser (λ = 632.8 nm). Scattering data were recorded in backscattering mode at a scattering angle of 2θ = 173°, which corresponded to a scattering vector of $q = 4\pi\, n/\lambda \sin\theta$ (0.02636 nm^{-1}).

3.11 Nanoparticle tracking:

Nanoparticle tracking analysis was performed on a Malvern Panalytical NanoSight LM14.
NTA Version: NTA 3.2 Dev Build 3.2.16
Script Used: SOP Standard Measurement 11-48-23AM 25J~
Capture Settings: Camera Type: sCMOS; Laser Type: Blue405; Camera Level: 11; Slider Shutter: 890; Slider Gain: 146; FPS 25.0; Number of Frames: 1498; Temperature: 22.8 - 23.0°C; Viscosity: (Water) 0.931 - 0.934 cP;

Analysis Settings: Detect Threshold: 15; Blur Size: Auto; Max Jump Distance: Auto: 7.5 - 14.3 pix.

4 Synthesis and characterisation of ZIFs

4.1 Preparation of the 1-aminopyrene-functionalized nanoparticulated ZIF-8/ZIF-90 hybrid (Z8P)

16.08 mg (0.075 mmol) of **1-aminopyrene** and 47.28 mg (0.493 mmol) of **Imidazolate-2-carboxaldehyde** were accurately weighed and transferred into a sealable glass flask. 5 mL of methanol was added using a 10 mL pipette, the glass flask was sealed with an aluminium cap and afterwards heated at 80°C for 30 min with stirring. After cooling the solution to ambient temperature, 40.4 mg (0.493 mmol) of **2-methylimidazole** was added to the solution as well as 250 µL of a nearly saturated solution of **MeONa** in methanol (0.25 g/mL) using a 1 mL pipette. Obtaining a well dissolved solution of **MeONa** requires heavy stirring or ensuring that the solution is being stirred while pipetting.

After the addition of MeONa, the solution was stirred for 1 minute. Finally, 5 ml of a **$Zn(NO_3)_2·6·H_2O$** solution was added using a 10 ml micropipette. This $Zn(NO_3)_2$ solution was prepared by weighting 146 mg of $Zn(NO_3)_2·6·H_2O$ (0.494 mmol) and transferring it to a glass vial, then adding 10 ml of methanol with a 10 ml pipette and mixing by shaking gently.

Almost immediately after the addition of the Zn salt solution, a yellow solid appeared dispersed in the solution. Stirring was stopped. The mixture was left undisturbed for 4 hours. A yellow solid sedimented to the bottom of the vial.

The sedimented mixture was redispersed in the solvent with a short burst of vigorous agitation and aliquots were quantitatively transferred into a 2 mL Eppendorf tube. The suspended solid was then sedimented via centrifugation (15000 rpm, 3 mins), the supernatant removed with a pipette and discarded. The process was repeated until all the reaction volume had been centrifuged. All the solid residue remained at the bottom of the Eppendorf.

Fresh methanol was added to fill up the Eppendorf tube, the solid residue was then redispersed by agitation, the suspensed solid was then sedimented via centrifugation (15000 rpm, 3 mins) and the coloured supernatant methanolic solution was removed. This procedure was repeated 5 additional times, until the methanolic solution became colourless. The solid residue was finally dried overnight at 5 mbar and 75 ^{o}C and let to cool down under vacuum.

For the three synthesises performed the final masses of **Z8P** obtained were: S1= 31.24 mg; S2= 30.02 mg; S3= 29.54 mg. When compared with the <u>initial 119.8 mg of total mass</u> weighted for synthesis (16.08 mg of **1-aminopyrene,** 47.28 mg of **Imidazolate-2-carboxaldehyde,** 40.4 mg of **2-methylimidazole** and 16.04 mg of **Zn^{2+}** [amount of Zinc coming from 73 mg of **$Zn(NO_3)_2·6·H_2O$**] it can be estimated that the yield of the synthesis, expressed in %mass, is of around **25% of reacted mass**.

WARNING: When handling Z8P solid to weight for HPLC analysis it must be kept away from humidity as much as possible.

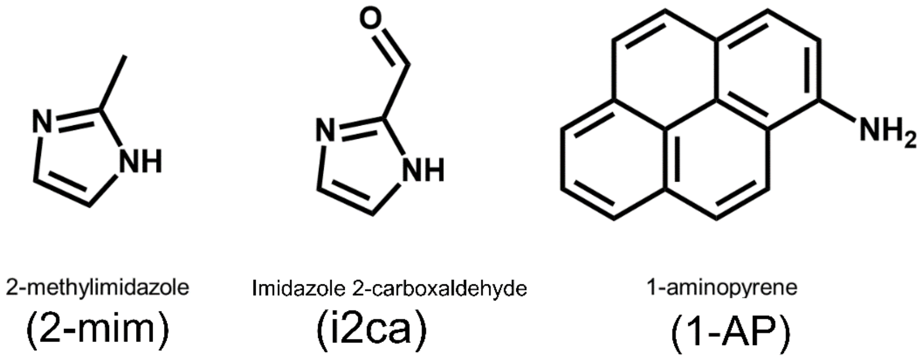

Fig. S1 *Chemical structures of the constituents to form Z8P, and their proposed abbreviations.*

4.2 Preparation of the nanoparticulated ZIF-90

94.56 mg (0.984 mmol) of **Imidazolate-2-carboxaldehyde** was weighed, transferred to a sealable glass flask with an aluminium cap. 5 mL of methanol was added, using a 10 mL pipette. The flask was sealed and then heated to 80°C until total dissolution (5 min) was achieved.

After the addition of MeONa, the solution was stirred for 1 minute. Finally, 5 ml of a **$Zn(NO_3)_2·6·H_2O$** solution was added using a 10 ml micropipette. This $Zn(NO_3)_2$ solution was prepared by weighting 146 mg of $Zn(NO_3)_2·6·H_2O$ (0.494 mmol) and transferring it to a glass vial, then adding 10 ml of methanol with a 10 ml pipette and mixing by shaking gently.

Almost immediately after the addition of the Zn salt solution, a white solid precipitated. The suspension was left undisturbed for 4 hours without stirring. The white solid sedimented to the bottom of the vial.

The mixture was redispersed with a short vigorous agitation and aliquots were transferred into a 2 mL Eppendorf tube. The suspended solid was then sedimented via centrifugation (15000 rpm, 3 mins), the supernatant removed with a pipette and discarded. The process was repeated until all the reaction volume had been centrifuged; all the solid residue remained at the bottom of the Eppendorf.

Then fresh methanol was added to fill the Eppendorf tube, the solid residue was then redispersed by vigorous shaking, the suspended solid was then sedimented via centrifugation (15000 rpm, 3 mins) and the methanolic supernatant removed. This procedure was repeated 5 additional times. The solid residue was finally dried overnight at 5 mbar and 75 ^{o}C, let to cool down under vacuum.

WARNING: When handling ZIF-90 solid to weight for HPLC it must be kept away from humidity as much as possible.

4.3 Preparation of ZIF-8

This synthetic procedure was the same as in SI-4.2, with the following difference:

82.20 mg (0.984 mmol) of **2-methylimidazole** (2-mim) was used instead of imidazolate-2-carboxaldehyde. No heating is required to dissolve 2-mim. It dissolves almost immediately.

4.4 Structural characterization of ZIFs

4.4.1 FTIR characterization of the synthesised ZIFs

We performed FTIR measurements, in order to verify that the aldehyde functionality is incorporated in the **Z8P**. We used **ZIF-8** as a negative control and **ZIF-90** as positive control for comparison. For the measurements of each sample, a small amount of the solid powder was deposited on the diamond accessory sample holder.

All the characteristic peaks for each of the samples are consistent with the published spectra[4-5]. The band corresponding to the aldehyde functionality on the Imidazolate-2-carboxaldehyde moiety (broad band at 1670 nm[6]) can only be observed on the **ZIF-90** and the modified ZIF-8, **Z8P** and is not present in the unmodified **ZIF-8** (Fig. S2).

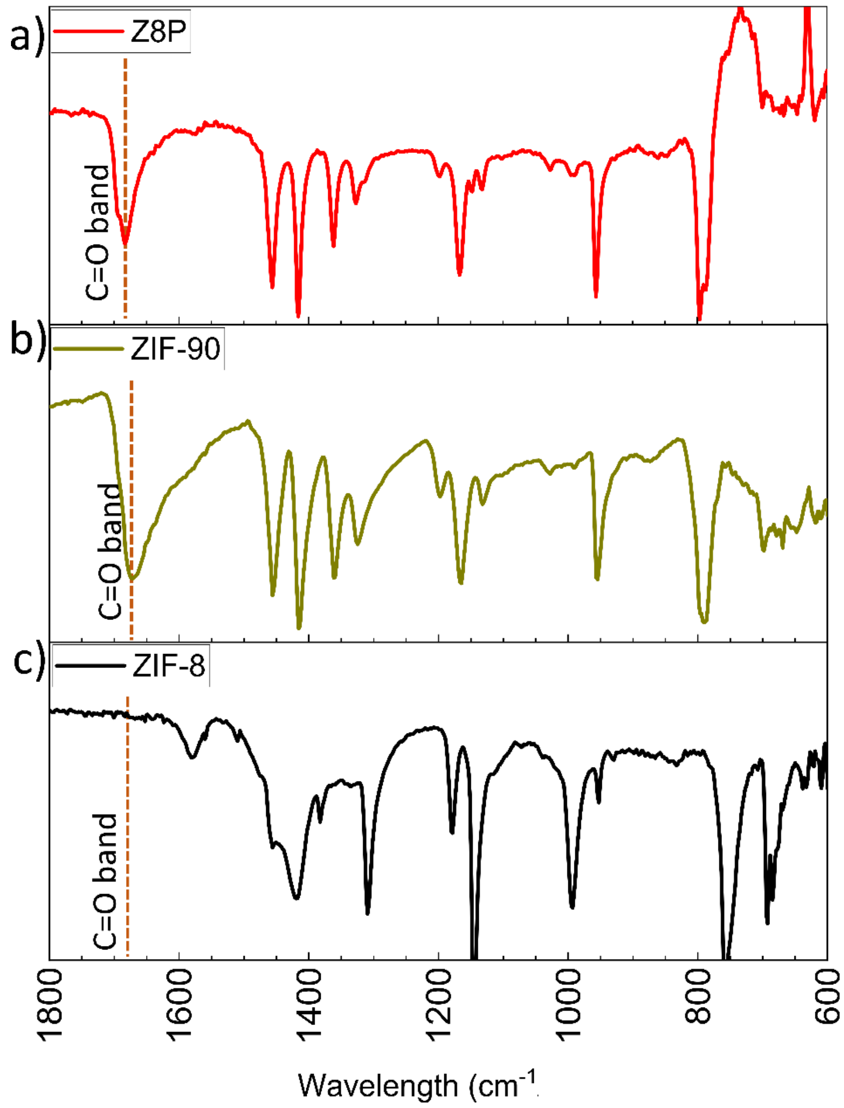

Fig. S2 FTIR spectra for a) Z8P, b) ZIF-90 and c) Z8P. The broad band at 1670 nm corresponds to the free aldehyde functionality of the Imidazolate-2-carboxaldehyde on the ZIF structure. The ZIF-90 has all the imidazole constituents with the aldehyde functionality, thus having the most intense band.

Table S1 For the ZIF-8 particles, there are four characteristic peaks at 1600, 1145, and 995 cm^{-1}, assigned to vibrations of C=N, and C−N in the imidazole ring, respectively. The intense peaks at 1457 and 1382 cm^{-1} correspond to the entire ring stretching. Several spectral bands seen in the range 1350 to 900 cm^{-1} can be ascribed to the in-plane bending of the ring and the peaks at 760 and 690 cm^{-1} are associated with aromatic sp^2 C-H bending. Finally, a strong band at 421 cm^{-1} was observed that corresponds to the Zn-N stretching as the zinc atoms in the ZIF-8 structure connect to nitrogen atoms of the 2-mim linker during the formation of ZIF-8.

Peak position (cm^{-1})	Vibration assigned to
1670	free aldehyde on i2ca
1600	C=N imidazole ring
1457	imidazole ring stretching
1382	imidazole ring stretching
1145	C-N imidazole ring
995	C-N imidazole ring
760	aromatic sp^2 CH bending
690	aromatic sp^2 CH bending
421	Zn-N stretching

4.4.2 Characterization of the ZIF particles and conjugate particles via TEM

Size and shape of the nanoparticles were analysed via TEM with respect to reported ZIFs[7] The TEM analysis were complemented with EDS.[8]

To prepare the **Z8P** sample, a dispersion of the **Z8P** ZIF in methanol (1 mg/mL) was prepared by weighing 4.97 mg of **Z8P** in a glass vial and adding 5 ml of methanol with a 10 ml pipette. After dispersion by ultrasonicating for 10 min, 10 µL aliquot of the methanol solution of the **Z8P** dispersion was deposited on a copper ultrathin C film on Lacey Carbon support film with a 400 mesh that was carefully deposited on top of a small glass crystallizer. The excess of solvent was left to evaporate open to the atmosphere, and the sample was then ready to be analysed.

To prepare the **ZIF-90** sample, a dispersion of the ZIF-90 in methanol (1mg/mL) was prepared by weighting 5.24 mg of **ZIF-90** glass vial and adding 5 ml of methanol with a 10 ml pipette; the rest of the process mirrors the TEM sample preparation described above for Z8P.

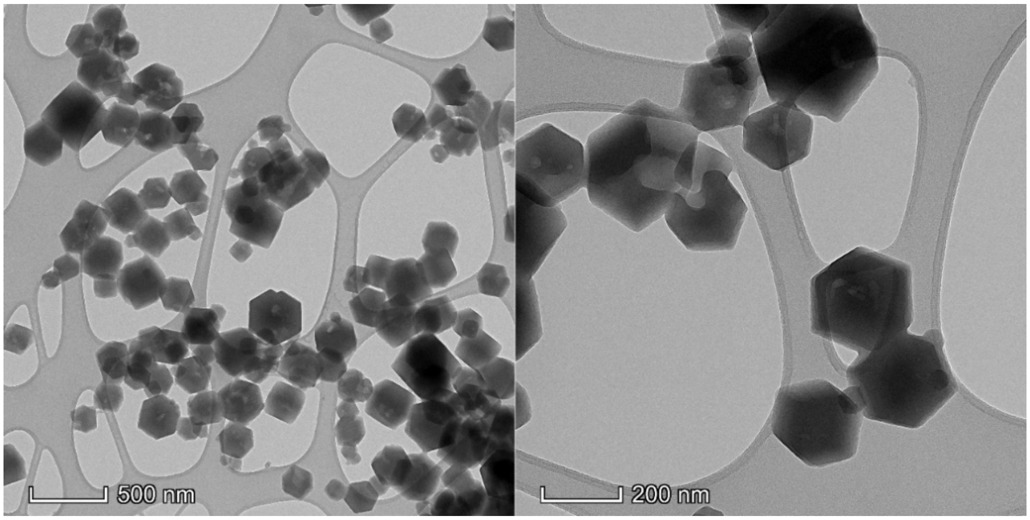

Fig. S3 TEM pictures for ZIF-90. The rhombic dodecahedral shape for ZIF particles[9] can be seen, with some polydispersity in size, between 30 to 350 nm and averaging 150 nm. These sizes are determined using only TEM results and are not absolute values.

The **ZIF-antibody conjugates** were also analysed by TEM measurements, complemented with S-TEM and EDS (Fig. S29) in Section SI-9. To prepare the sample, 100 µL of a borate buffer dispersion of **Z8P-IgG** (0.75 mg/mL, SI-6.1) was transferred to a 2 mL Eppendorf tube with a 200 µL pipette and centrifuged at 15000 rpm for 3 min. The supernatant was carefully removed and the sedimented solid was then redispersed in fresh methanol (200 µL). A 10 µL aliquot was deposited on a copper ultrathin C film on Lacey Carbon support film with a 400 mesh. This film was carefully deposited on top of a small glass crystallizer. The excess of solvent was left to evaporate, and the sample was then ready to be analysed by TEM.

4.5 Synthesis of the imine formed by Schiff condensation of 1-AP and i2ca

To study the imine formation, 200 mg of **1-AP** (0.8755 mmol) and 86.06 mg of **i2ca** (0.875 mmol) were transferred into an aluminium-capped sealable glass flask. 10 mL methanol was added. The solution was then heated at 80 °C for 30 min with slow stirring (300 rpm) on a magnetic stirrer. The **imine** precipitated as an intense yellow-mustard solid. After the reaction was completed, the suspension was allowed to cool down to room temperature while stirring. The suspension was transferred quantitatively to a 15 mL Falcon tube and the solid sedimented via centrifugation (4000 rpm, 5 min). The supernatant was carefully removed and discarded. At this stage there maybe residual starting materials.

The wash workup is as follows. 5 mL of a fresh methanol was added to the sedimented residue and redispersed by strong agitation; the suspension was the centrifuged to sediment the solid. After removing the supernatant, the open Falcon tube was subjected to vacuum for 3 hours at 5 mbar and 60°C in a vacuum oven. Some impurities can be observed on the NMR measurements (Fig S6) which may have resulted from the preparation method to obtain imines

Caution: Perform the wash procedure with methanol only once to prevent the imine from dissolving in methanol, hence reducing the final yield of the imine.

4.6 Characterisation of 1-AP, i2ca and the imine formed by Schiff condensation of 1-AP and i2ca

4.6.1 Characterization by 1H NMR

^{1}H NMR was used to confirm the structures of **1-AP** and **i2ca** and to characterise the structure of the **imine** and assess its purity.

All samples were prepared using the same protocol: a small amount of each analyte (12.54 mg for **i2ca**, 13.54 mg for **1-AP** and 11, 54 mg for the **imine**) was transferred into a 5 mL glass vial, then the contents of a 0.75 mL d_6-DMSO ampule was quantitatively transferred into the glass vial, and after achieving complete dissolution, the solution was transferred using a Pasteur pipette into an NMR tube.

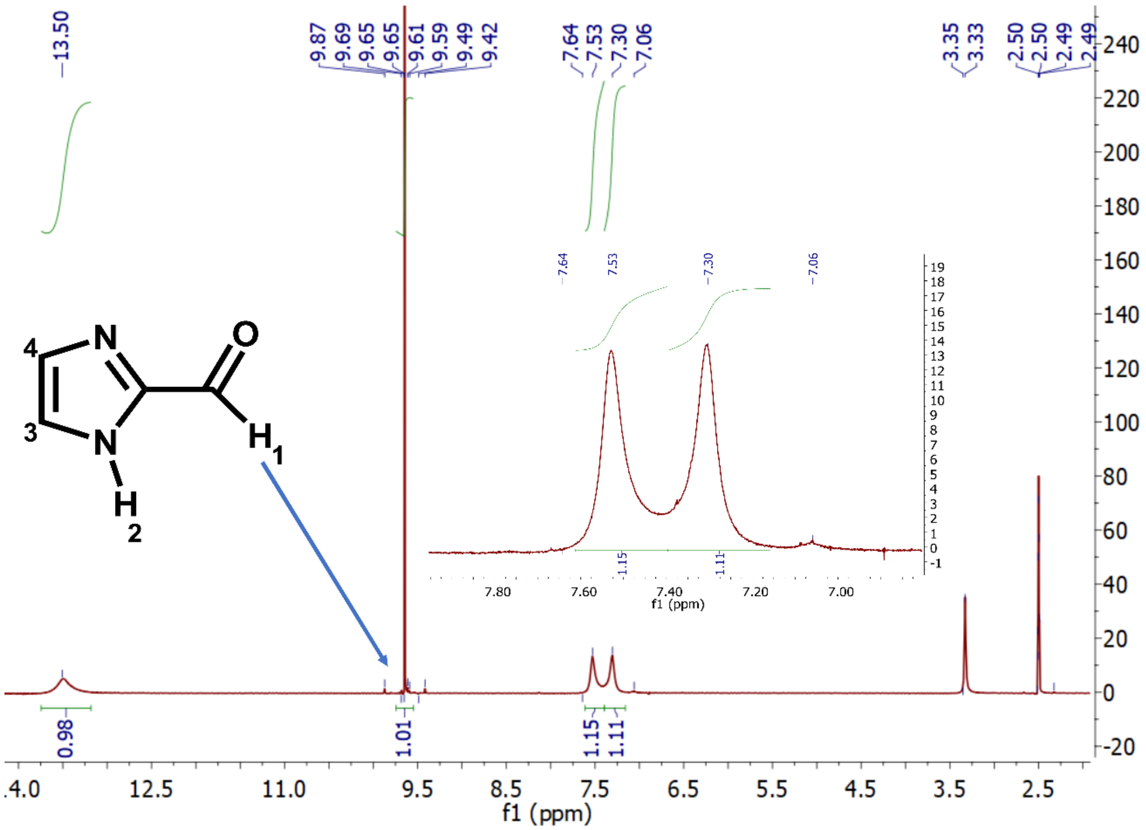

Fig. S4 ^{1}*H NMR spectra for Imidazolate-2-carboxaldehyde (i2ca)*

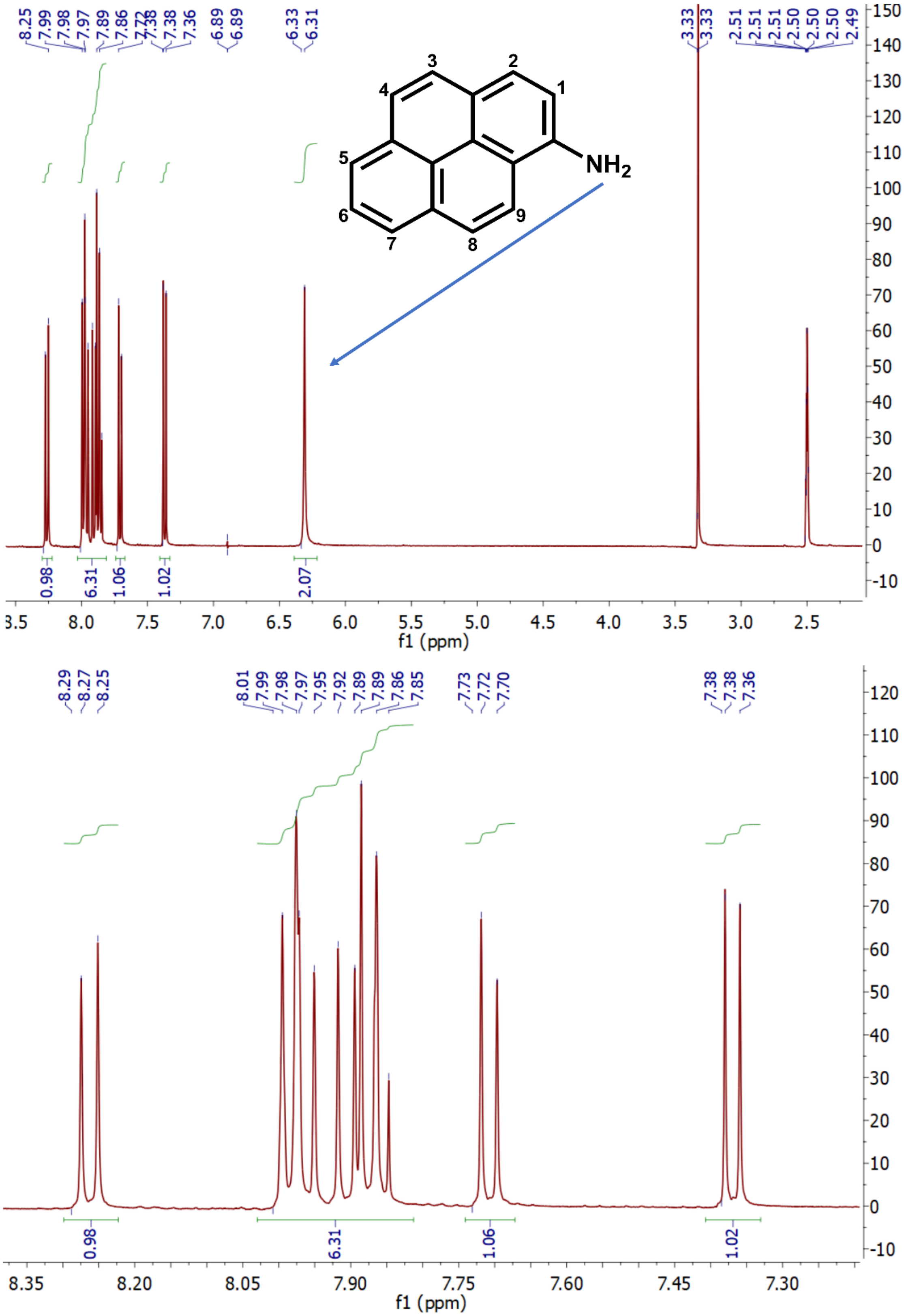

Fig. S5 *^{1}H NMR spectra for 1-aminopyrene (1-AP)*

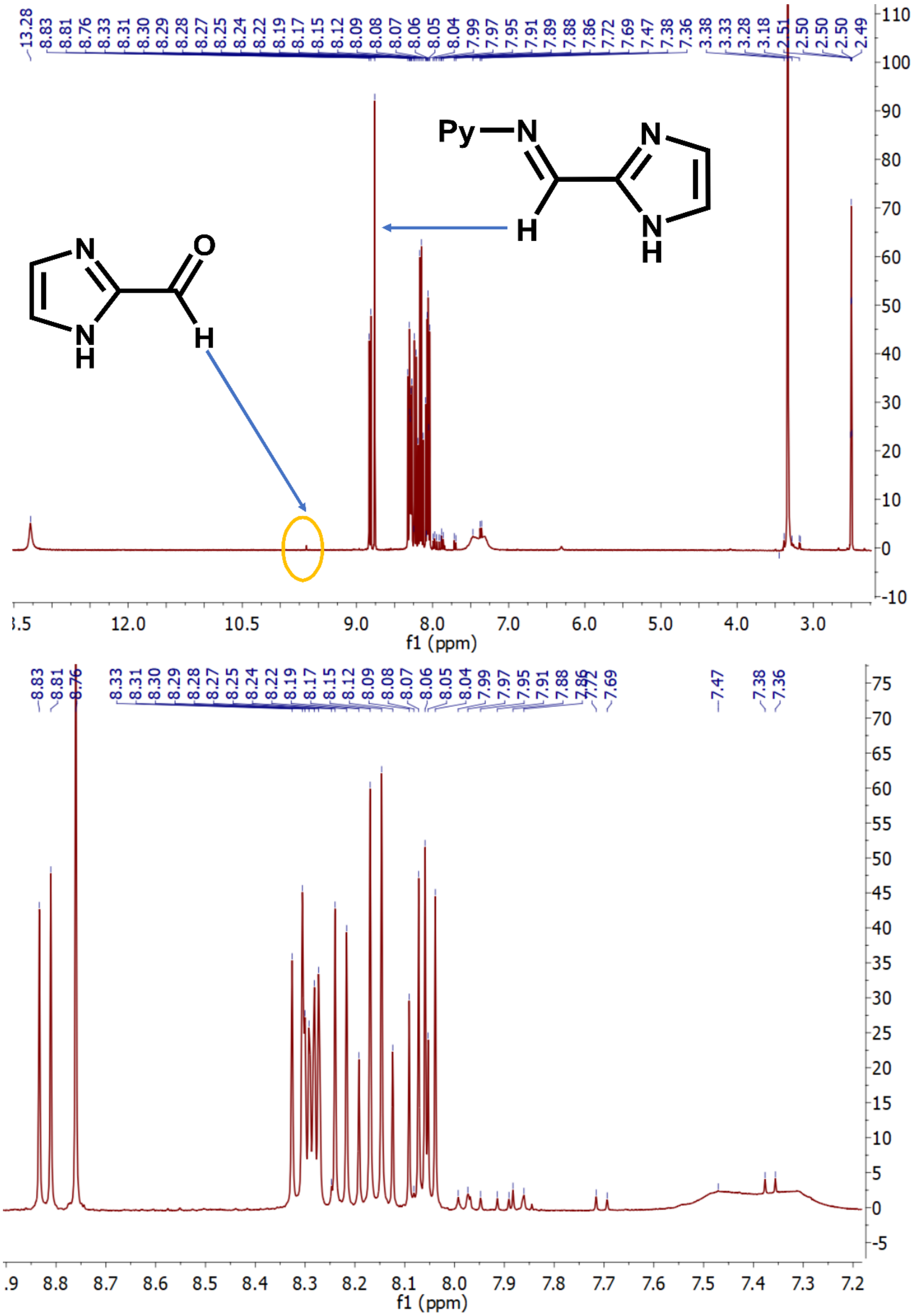

Fig. S6 [1]*H NMR spectra for the imine formed between i2ca and 1-AP*

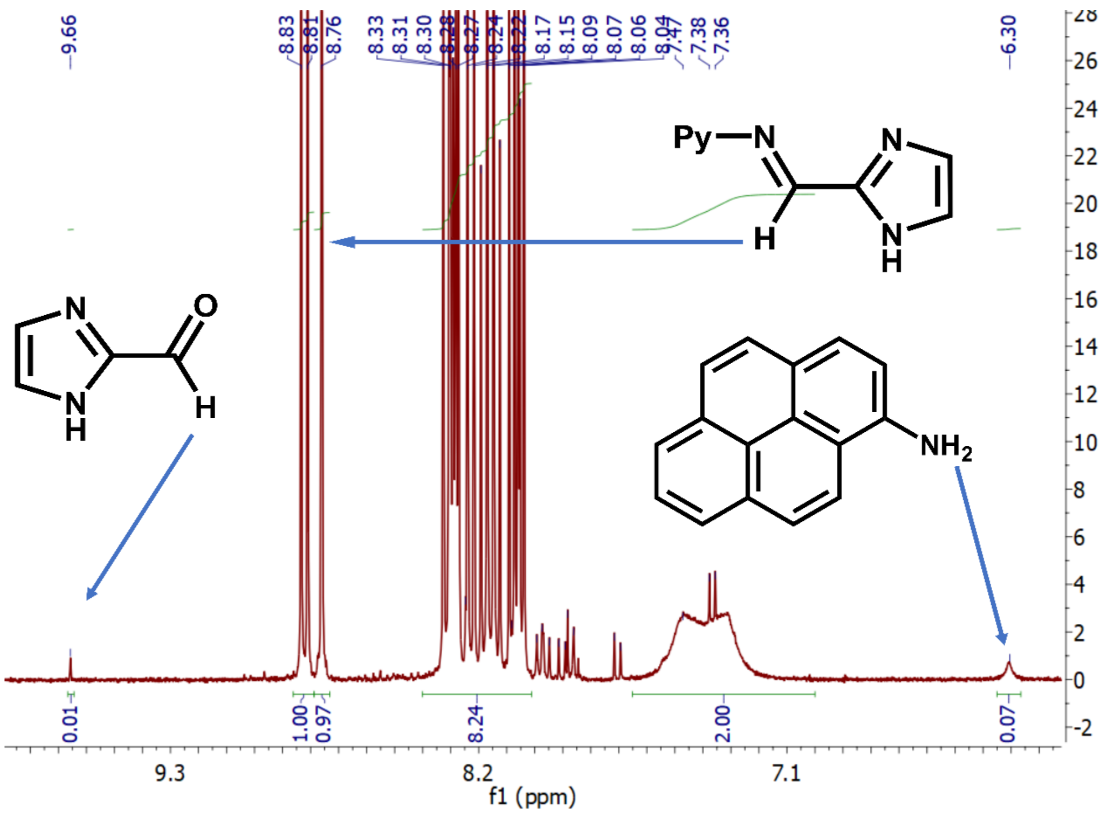

Fig. S7 ^{1}*H NMR spectra for the imine, focused on a range of ppms that allows for an estimation of the impurity of the imine.*

<u>Interpretation of NMR characterisation of the imine:</u>

i2ca: ^{1}H NMR (DMSO, 400 MHz): δ 7.308 and 7.531 (2 d, 2H, H3 & H4 respectively; <u>H3 and H4 are two unresolved doublets, theoretical calculations set the J value at approximately 4 Hz</u>), **9.647 (s, 1H, H1)**, 13.501 (s br, 1H, H2; <u>interchangeable proton</u>)

1-AP: ^{1}H NMR (DMSO, 400 MHz): δ **6.31 (s, 2H, NH2)**, 7.38 (d, J = 8.3 Hz, 1H, H2), 7.72 (d, J = 8.8 Hz, 1H, H4), 7.85 (t, J = 7.7 Hz, 1H, H6), 7.89 (d, J = 8.8 Hz, 1H, H3), 7.90 (d, J = 9.3 Hz, 1H, H9), 7.96 (d, J = 8.4 Hz, 1H, H2), 8.00 (d, 1H, H5), 8.27 (d, 1H, J = 9.2, H9)

Imine: ^{1}H NMR (DMSO, 400 MHz): δ 7.2-7.6 (2 d, 2H, H3 &H4 i2ca*), 8.038-8.326 (m, 8H), **8.760 (s, 1H, Imine H1)**, 8.823 (d, 1H, J = 9.2, H9 1-AP), 13.283 (s br, 1H, H2 i2ca)

In the ^{1}H NMR spectrum for **1-AP** matches the one reported on the literature[10] (See Fig. S5), and it has a strong singlet that can be seen at 6.311 ppm, corresponding to the 2 protons of the amino group of aminopyrene.

In the ^{1}H NMR spectrum of the **imine** (Fig. S6) this peak at 6.311 ppm is almost completely gone (a very small peak can be seen), thus indicating the significant reduction of the amino group. When comparing the spectra of **i2ca** and the **imine**, the peak corresponding to the proton on the aldehyde functionality on **i2ca** can be seen at 9.647 ppm as a singlet. This singlet is also almost completely gone in the imine, instead a new singlet appears at 8.760 ppm, corresponding to the proton on the carbon of the C=N bond of the **imine**.

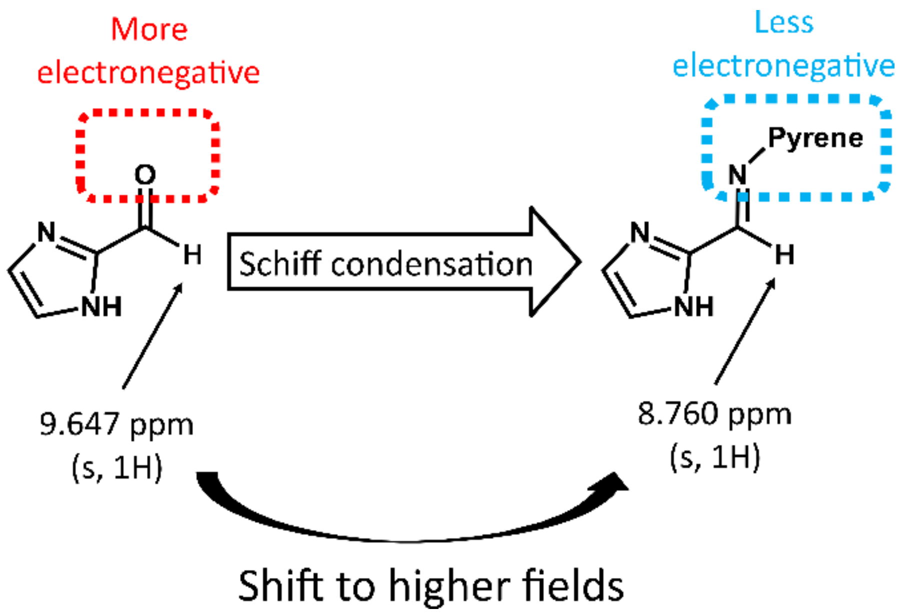

Fig. S8 Scheme representing the chemical shift on ^{1}H NMR that the aldehyde proton on the imidazole moiety suffers during the Schiff base condensation. The replacement of the aldehyde for an imine functionality reduces the electronegativity of the carbon, shifting the peak to higher fields

We can also see that the imine is not completely pure (Fig. S7), since there is around 1% of i2ca and around 7.5% of 1-AP present on the mixture. This is most likely indicative of the imine not fully washed with the methanol, as well as maybe suffering small amounts of hydrolysis with the water molecules present on the system.

4.6.2 Characterization of imine by PXRD

Other evidence of the formation of the imine is supported by comparing the PXRD analysis of **1-AP**, **i2ca** and the **Imine** (Fig. S9). The new imine has a different crystallographic structure to that of the precursors.

To prepare the samples for PXRD analysis, each powder was softly ground with an agar pestle on an agate mortar, the homogeneous fine powder was then placed on a Bragg-Brentano PVC sample holder with an opening of 0.8 cm in diameter, the cavity was filled with the powder and the top surface of the sample was compacted flat and flash with the top surface of the holder with the help of a glass slide.

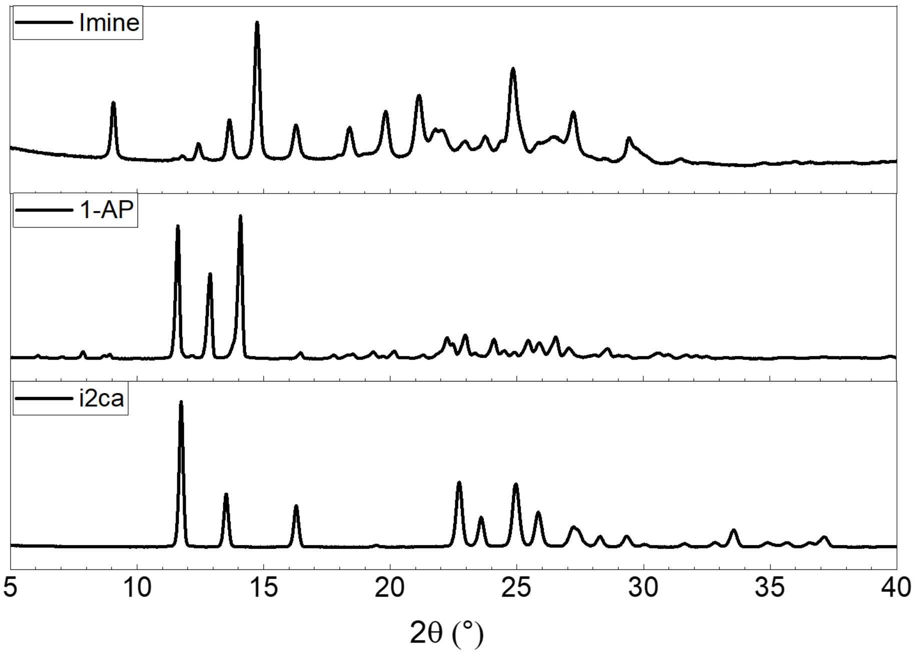

Fig. S9 Powder diffractograms for Imidazolate-2-carboxaldehyde (i2ca, bottom), 1-aminopyrene (1-AP, middle) and the imine formed between i2ca and 1-AP (imine, top). The new crystalline phase obtained for the imine proves the formation of a new compound.

5 Additional analysis and characterisation of ZIF and ZIF conjugates

We complement the studies and characterization for our **ZIFs** and **ZIF-IgGs** with more relevant analytical techniques: fluorescence of the Z8P-IgG conjugate particles (SI-5.1), organic composition of the ZIF-90 and Z8P particles (SI-5.2), metallic composition of the Z8P particles (SI-5.3), crystal structure of the ZIFs and ZIF conjugates (SI-5.4).

5.1 Fluorescence characterization the conjugated Z8P particles versus DMP

An experiment was designed using the analytical conditions developed in previous work.[11] to assess the changes in fluorescence quenching of the conjugate, **Z8P-IgG**, when exposed to a model contaminant, dimethyl phthalate, DMP.

In each well of a black non-binding polypropylene 96-well black microtiter plate, 10 µL of a **Z8P-IgG** dispersion in borate buffer (0.75 mg/mL, SI-6.1), was pipetted. The respective aliquots of a **DMP stock solution** listed in Table S2 are added to each well and then brought to a final volume of 210 µL with borate buffer. Each well had a final concentration of 47 µg/mL of Z8P-IgG. The excitation wavelength was 335 nm, emission wavelength was observed at 445 nm. The gain was set to 100%.

2 mg/mL DMP stock solution preparation: 16.8 µL of the commercial **DMP** solution (d = 1.19 g/mL) was added to a sample glass vial containing 10 ml of borate buffer and homogenized with stirring.

CAUTION: DMP is an endocrine disrupting chemical, avoid skin contact and handle with care.

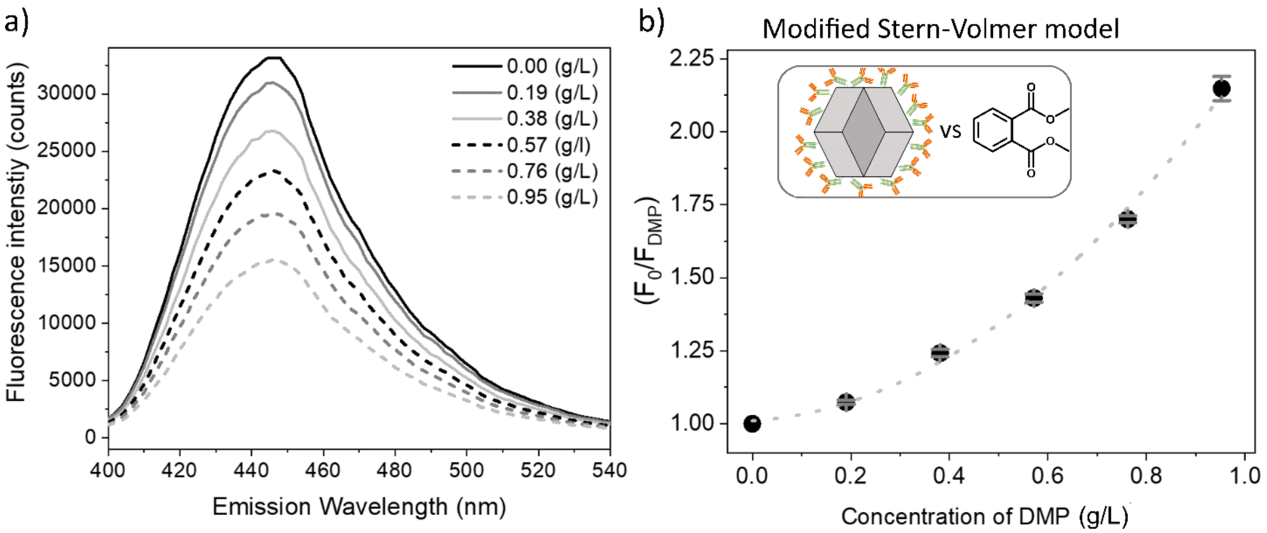

Fig. S10 a): Fluorescence spectra for Z8P−IgG (with a constant concentration of 47 µg/mL) when dispersed in borate buffer with increasing concentrations of dimethyl phthalate (DMP, 0.0-1.0 g/mL). b): Modified Stern-Volmer fitting curve for the fluorescence quenching of Z8P−IgG versus the DMP concentrations expressed as g/L. The excitation wavelength was 335 nm, and the emission wavelength maximum was recorded at 445 nm. Each data point represents the mean of duplicate measurements.

Table S2 Fluorescence Intensity of Z8P-IgG and relative changes (F_o/F_{DMP}) observed to different concentrations of dimethyl phthalate. Final concentration for Z8P-IgG in borate buffer was 47 µg/mL in all wells. The fluorescence intensity was read at 445 nm and converted to the necessary mathematical form ("F_o/F_{DMP}"), where "F_0" is the intensity of fluorescence without the quencher (DMP) and "F_{DMP}" is the intensity of fluorescence with a specific concentration of the quencher, in order to apply the modified Stern-Volmer equation (F_o/F_{DMP}). Duplicates for each system were measured and the standard deviation (S.D.) calculated.

DMP sample preparation		Fluorescence determination		
DMP conc. in borate buffer. (g/l)	DMP stock in borate buffer; Aliquots taken (µL)	Fluorescence intensity (F_{DMP}) at 445 nm (counts)	F_o/F_{DMP}	S.D.
0	0	33114 (F_0)	1.00	0.000
0.19	20	30838	1.07	0.007
0.38	40	26660	1.24	0.013
0.57	60	23149	1.43	0.015
0.76	80	19471	1.70	0.013
0.95	100	15414	2.15	0.041

The fact that the fluorescence emission for the Z8P-IgG vs DMP fits a modified Stern-Volmer model that static and dynamic quenching are happening[12-13]. This could indicate that the sensing is happening on two different areas of the particle: on the surface, the exposed pyrene cores suffer collisional quenching, this being the dominant contribution, and in the bulk, the phthalates can diffuse through and be trapped with the pyrenes. However, when considering that the absorption spectra for the DMP[14] and for the imidazolate species forming the modified ZIF (Figure S11) showed they do not absorb at the bands for excitation and emission of the imine within the Z8P ZIF, it must be assumed that the quenching is not due to quenching effects by absorption of the emitted radiation by the imine. Instead, we believe a π- π stacking that leads to the formation of an exciton state that no longer absorbs radiation. The formation of a π- π stacking exciplex between the imine and the DMP is driven by aggregation often due to hydrophobic effects—the dye molecules stack together to minimize contact with water. Planar aromatic molecules that are matched for association through hydrophobic forces can enhance static quenching. The formation of an exciplex via π- π stacking of the DMP and the imine needs further studies to be confirmed.

We also postulate that an “inner-filter quenching”[15] is happening on the Z8P ZIF nanoparticles. The fluorescence of the aminopyrene moieties in the Z8P particles are quenched as a result of the tight packing of the dye inside the ZIF, where it leads to an inner filter effect. Part of the filter effect is also caused by absorbing moieties in the antibodies surrounding the particles. This inner filter effect has also been reported for other nanocarrier systems such as liposomes[16]. More evidence supporting this inner filter effect will be shown later, when the breakdown of the Z8P in Z8P-iGg, and liberation of the dye, leads to an increase of the fluorescence emission (Figure S22).

The effect the antibodies exert on the Z8P particles surface is more difficult for us say, since it would be likely that the antibodies can absorb part of the incident excitation radiation, as well as quenching the fluorophores on the surface via excimer formation (some amino acids have aromatic moieties).

5.2 HPLC procedure to obtain the chemical composition of ligands in Z8P

To quantify the concentration of the organic ligands in Z8P, we selected phosphate buffer as the aqueous solvent (solvent A) for HPLC because it is the only buffer that is completely transparent at 210 nm, which was the wavelength required to detect the presence of **i2ca** by HPLC.

The pH was chosen considering the pK_a values of the imidazole species (7.46 for **2-mim** and for **i2ca**) to make them less-dissociated and therefore more lipophilic, increasing their retention time on reverse phase HPLC. It was possible to achieve baseline separation for both species at the selected pH of 8.8. (Fig. S12).

HPLC column: Excel C18-PFP with 2 µm particle size (4.6 mm internal diameter × 50 mm length) manufactured by ACE (SI-3.6).

Caution: This Excel C18-PFP column has been manufactured to be stable up to pH 10. However, to avoid unnecessary damage to the column and the HPLC system, both should be flushed after use for at least 20 minutes at a low flowrate with water/methanol to remove damaging residues from buffer which could crystallise in the HPLC equipment and column and also to avoid subjecting the silica-based column to damaging alkaline conditions.

The HPLC method is explained in SI-3.6.1.

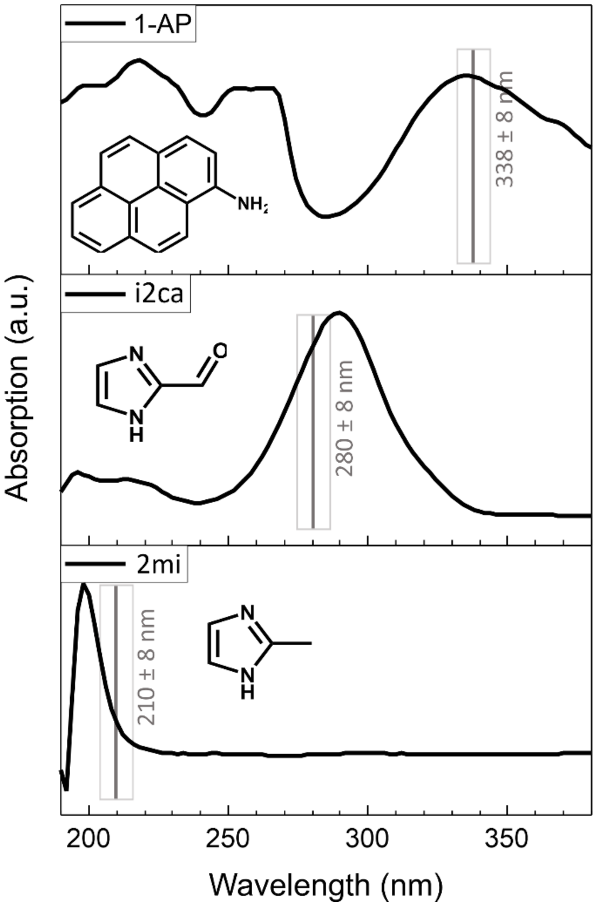

Fig. S11. DAD absorption spectra for the analytes constituting the ZIFs of interest highlighting the wavelengths and wavelength range at which each ligand was determined

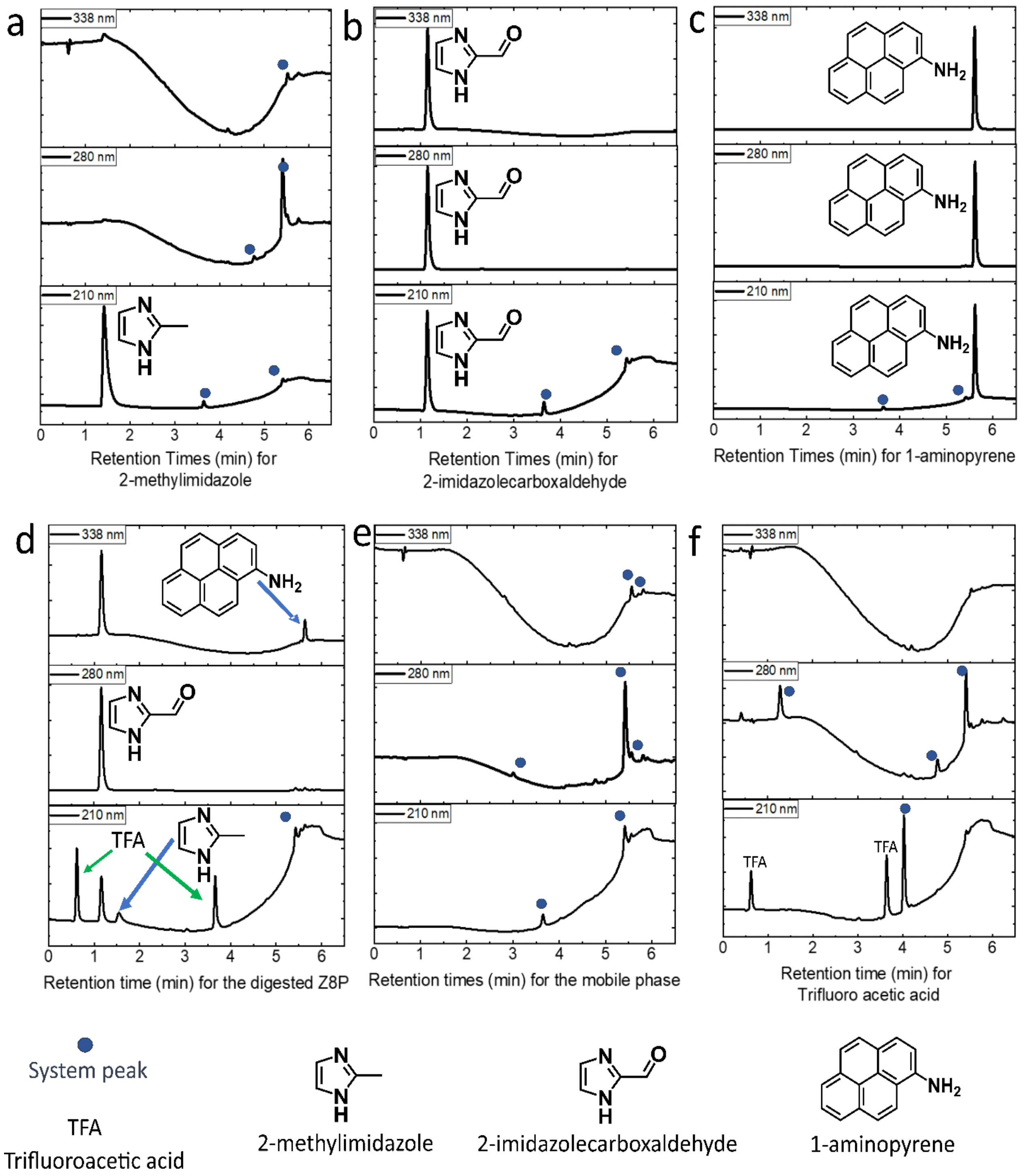

Fig. S12. *Chromatograms of all the species of interest (2-mim [a], i2ca [b], 1-AP [c], the digested Z8P [d], the mobile phase [e], and the mobile phase with TFA [f]). For all species all three chosen wavelengths were shown (from top to bottom; 338 nm, 280 nm and 210 nm). In Figure (d) the positions of all peaks on the digested ZIF was reported; the retention time for 2-mim was 1.53 min, for i2ca was 1.16 min, and for 1-AP was 5.63 min. TFA has two signals at 210 nm, one at 0.65 min and another one at 3.71 min.*

5.2.1 Preparation of Standard solutions

Standards stock solutions for the quantification of imidazolate-2-carboxaldehyde (**i2ca**) and 1-aminopyrene (**1-AP**) by HPLC were prepared as follows.

Weigh 25 mg of the commercially available starting materials (97% purity for **i2ca** and 95% for **1-AP**) and transfer each to a separate glass flask. Dissolve each in 8 mL of methanol. Full dissolution is only achieved by heating each of them to 60 °C in the sealed glass flask. Once dissolved, allow the solution to cool down to room temperature. Quantitatively transfer the content of the flask to a 10 mL graduated flask. Use 1 mL methanol to clean the glass flask and transfer this rinse solution to the 10 mL graduated flask. Make the solution up to 10 mL volume with methanol. One additional standard solution for each species was independently prepared to confirm that the working standard can be reproducibly prepared.

The stock standard (Stock 1) which will be later used to prepare the set of calibration standard solutions and the duplicate independent standard (Stock 2) were prepared as follows:

For **i2ca**: **Stock 1:** 24.20 mg (0.249 mmol) in 10 mL methanol. This stock was used in the preparation of the linearity standard solutions. **Stock 2**: 25.87 mg (0.261 mmol) in 10 mL methanol was independently prepared, and used to prepare Std 5bis in Table S3.

For **1-AP**: **Stock 1:** 25.70 mg (0.112 mmol) in 10 mL methanol. This stock was used in the preparation of the linearity standard solutions. **Stock 2**: 25.03 mg (0.109 mmol) in 10 mL methanol was independently prepared, and used to prepare Std 5bis in Table S3.

Table S3 Procedure to prepare the Standard solutions in methanol (mg/mL) used for the calibration curve. The calibration standard solutions Std 1 to Std 8 were prepared by dilution from Stock 1 of 2-mim, i2ca or 1-AP while Std 5b were prepared in duplicate from the independent Stock 2

	Concentration of analyte **[2-mim, i2ca or 1-AP]** (mg/mL)	Stock Solution (2.5 mg/mL) **[2-mim, i2ca or 1-AP]** aliquot added of each analyte (µL)	Aliquot added of **methanol** to bring solution to 1000 µL (µL)
Blank	0.0	0	1000
Blank	0.0	0	1000
Std 1	0.4	160	840
Std 2	0.7	280	720
Std 3	1.0	400	600
Std 4	1.3	520	480
Std 5	1.6	640	360.
Std 5 Bis	1.6	640	360
Std 5 Bis	1.6	640	360
Std 6	1.9	760	240
Std 7	2.2	880	120
Std 8	2.5	1000	0

5.2.2 Validation of the HPLC method: Linearity

Linearity tests for each compound were carried out using a series of 8 calibration standard solutions (Std 1 to Std 8) ranging from 0.4 to 2.5 mg/mL. Each calibration standard solution was prepared by pipetting the indicated volumes on Table S3 from a 2.5mg/mL (stock 1), then adding the corresponding methanol aliquot to accurately make a total volume of 1.000 µL.

The concentration of each working standard solution was calculated from the known concentration of Stock 1 or Stock 2. The peak area obtained by HPLC for each working standard solution was plotted against the known concentration of the calibration standard solution used. The concentrations range chosen demonstrates good linearity for all the analytes (Fig. S13).

Once linearity has been demonstrated as part of the validation of the HPLC method, all subsequent analyses can be performed by using just one working standard solution prepared at the expected concentration ensuring that the concentration of the working standard and sample solutions are within the demonstrated linearity range. It is desirable to independently weigh 2 working standards to demonstrate that they have been accurately prepared. It is best to prepare the working standard directly and not by dilution of a stock solution. It is recommended to freshly prepare the working standard every time.

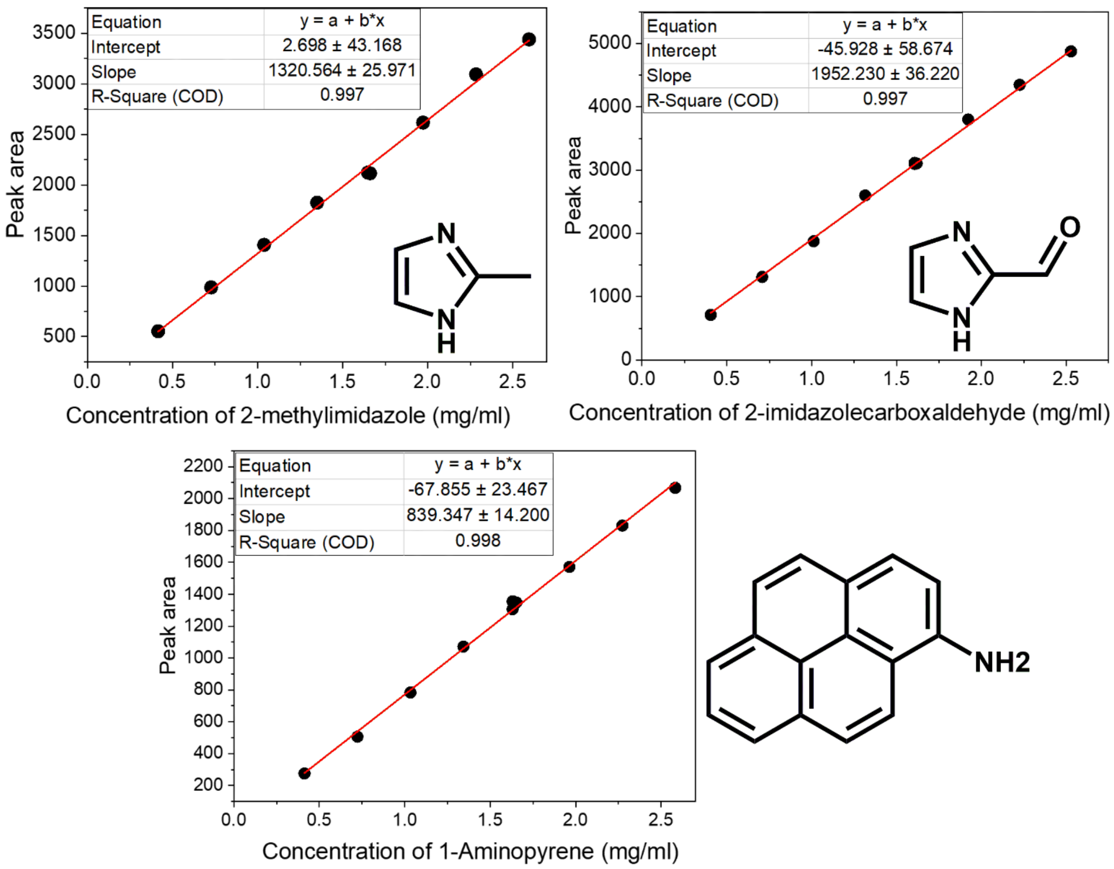

Fig. S13. Linear calibration curves for each of the analytes of interest (2-mim, top left; i2ca, top right; 1-AP, bottom).

5.2.3 Preparation of Z8P acid digestion sample solutions

This protocol was done for each **Z8P** batch in triplicate. Approximately 4 mg of **Z8P** were weighed, transferred to a glass vial and dispersed in 1.5 ml of MeOH. Then 150 µL of concentrated trifluoroacetic acid (TFA) measured with a 500 µL glass syringe with metallic needle was added.

Note: after adding the TFA to the methanolic dispersion of the ZIF, the solution will turn to an intense yellow-mustard.

After complete dissolution of the ZIF, an additional 1.3 ml of MeOH is added, then 1.8 mL of a 1 M NaOH solution in water was added using a 2.5 mL automatic pipette, to neutralize the solution. We made sure, using a laser pointer, that there were no colloids present in the solution. We also made sure that the pH of the solution remained slightly acidic (around pH 3-5) using pH paper to avoid any reprecipitation. After cooling, the solution was transferred to a 5 mL volumetric flask and the volume was adjusted with a Pasteur pipette using deionized water as solvent.

Note: after 2-3 min of time the colour of the solution will turn from an intense yellow-mustard to almost completely transparent and colourless.

1 mL volume was transferred to a 2 mL HPLC glass vial using a 1 mL pipette. Each sample was injected on to the HPLC column in duplicate.

The results of the HPLC quantification are shown on Table S6, using the peak areas obtained with the HPLC method previously described to calculate their concentration in relation to the peaks areas obtained from the working standard solutions of known concentration. We decided to calculate the final composition of the Z8P ZIF in relation to Zn^{2+} ions. We believe that this assist in a clearer understanding of the relative composition.

Table S4 Peak areas determined by HPLC for each of the working standard solutions for each of the 3 compounds. Two different solutions from two independent weights where used.

Compound	Mass (mg) (mg)	Volume (ml)	Concentration ($mg*mL^{-1}$)	Peak area (counts)	wavelength
2-mim_inj.1	19.67	20	0.9835	2319	**210 nm**
2-mim_inj. 2	19.67	20	0.9835	2299	**210 nm**
Average			**0.9835**	**2309**	
% Rel.St.Dev.				*0.6*	
i2ca_inj. 1	12.15	10	1.215	4010	**280 nm**
i2ca_inj. 2	12.15	10	1.215	4089	**280 nm**
Average			**1.215**	**4049.5**	
% Rel.St.Dev.				*1.4*	
1-AP_inj. 1	11.05	10	1.105	1334	**338 nm**
1-AP_inj. 2	11.05	10	1.105	1312	**338 nm**
Average			**1.105**	**1323**	
% Rel.St.Dev.				*1.2*	

Table S5. Peak areas obtained by HPLC for the sample solution of 2-minm, i2ca and 1-AP from the digestion of Z8P. Sample solution were injected in duplicate.

Sample	Mass	Volume	Concent.	2-mim Peak Area	i2ca Peak Area	1-AP Peak Area
	(mg)	(ml)	mg/mL	210 nm	280 nm	338 nm
Z8P S1_1_inj.1	3.20	5.00	0.64	205.00	1214.00	35.83
Z8P S1_1_inj.2	3.20	5.00	0.64	207.00	1225.00	36.64
Average				**206.00**	**1219.50**	**36.24**
% Rel.St.Dev.				*0.69*	*0.64*	*1.58*
Z8P S1_2_inj.1	3.22	5.00	0.644	202.00	1197.00	34.62
Z8P S1_2_inj.2	3.22	5.00	0.644	206.00	1228.00	36.46
Average				**204.00**	**1212.50**	**35.54**
% Rel.St.Dev.				*1.39*	*1.81*	*3.66*
Z8P S1_3_inj.1	3.61	5.00	0.722	226.00	1340.00	40.46
Z8P S1_3_inj.1	3.61	5.00	0.722	232.00	1377.00	41.39
Average				**229.00**	**1358.50**	**40.93**
% Rel.St.Dev.				*1.85*	*1.93*	*1.61*
Average: 6 values				**213.00**	**1263.50**	**37.57**
% Rel.St.Dev.				*5.9*	*6.0*	*7.2*
Z8P S2_1_inj.1	4.23	5.00	0.846	296.00	1526.00	46.92
Z8P S2_1_inj.2	4.23	5.00	0.846	308.00	1593.00	48.22
Average				**302.00**	**1559.50**	**47.57**
% Rel.St.Dev.				*2.81*	*3.04*	*1.93*
Z8P S2_2_inj.1	3.23	5.00	0.646	245.00	1185.00	30.82
Z8P S2_2_inj.2	3.23	5.00	0.646	247.00	1196.00	32.27
Average				**246.00**	**1190.50**	**31.55**
% Rel.St.Dev.				*0.57*	*0.65*	*3.25*
Z8P S2_3_inj.1	3.79	5.00	0.758	264.00	1380.00	42.00
Z8P S2_3_inj.1	3.79	5.00	0.758	271.00	1413.00	43.26
Average				**267.50**	**1396.50**	**42.63**
St.Dev.				*1.85*	*1.67*	*2.09*
Average: 6 values	3.34	5.00		**271.83**	**1382.17**	**40.58**
% Rel.St.Dev.	0.21	0.00		*9.4*	*12.1*	*18.2*
Z8P S3_1_inj.1	3.78	5.00	0.756	297.00	1331.00	42.78
Z8P S3_1_inj.2	3.78	5.00	0.756	301.00	1349.00	43.60
Average				**299.00**	**1340.00**	**43.19**
% Rel.St.Dev.				*0.95*	*0.95*	*1.34*
Z8P S3_2_inj.1	4.13	5.00	0.826	319.00	1431.00	47.41
Z8P S3_2_inj.2	4.13	5.00	0.826	337.00	1524.00	48.85
Average				**328.00**	**1477.50**	**48.13**
% Rel.St.Dev.				*3.88*	*4.45*	*2.12*
Z8P S3_3_inj.1	3.95	5.00	0.79	309.00	1397.00	46.94
Z8P S3_3_inj.1	3.95	5.00	0.79	317.00	1437.00	48.44
Average				**313.00**	**1417.00**	**47.69**
% Rel.St.Dev.				*1.81*	*2.00*	*2.22*
Average: 6 values				**313.33**	**1411.50**	**46.34**
% Rel.St.Dev.				*4.6*	*4.9*	*5.5*

Table S6 Calculation of the concentration of 2-min, i2ac & 1-AP as % g reactant per g Z8P. Zn2+ content is estimated as 100-concentration of 2-min, i2ac & 1-AP

Sample	2-mim	i2ca	1-AP	Estimated Zn^{2+} (100 -% ligands)
	%g/g Z8P	%g/g Z8P	%g/g Z8P	%g/g Z8P
Z8P S1_1_inj.1	13.6	56.9	4.7	24.8
Z8P S1_1_inj.2	13.8	57.4	0.0	28.8
Average	**13.7**	**57.2**	**2.3**	**26.8**
St.Dev.	*0.7*	*0.6*	*141.4*	*10.6*
Z8P S1_2_inj.1	13.4	55.8	4.5	26.4
Z8P S1_2_inj.2	13.6	57.2	0.0	29.2
Average	**13.5**	**56.5**	**2.2**	**27.8**
St.Dev.	*1.4*	*1.8*	*141.4*	*7.1*
Z8P S1_3_inj.1	13.3	55.7	4.7	26.3
Z8P S1_3_inj.2	13.7	57.2	0.0	29.1
Average	**13.5**	**56.5**	**2.3**	**27.7**
St.Dev.	*1.9*	*1.9*	*141.4*	*7.1*
Average: 6 values	**13.6**	**56.7**	**2.3**	**13.8**
% Rel.St.Dev.	*0.9*	*0.7*	*2.4*	96.0
Z8P S2_1_inj.1	14.9	54.1	4.6	26.3
Z8P S2_1_inj.2	15.5	56.5	0.0	28.0
Average	**15.2**	**55.3**	**2.3**	**27.2**
% Rel.St.Dev.	*2.8*	*3.0*	*141.4*	*4.3*
Z8P S2_2_inj.1	16.2	55.0	4.0	24.8
Z8P S2_2_inj.2	16.3	55.5	0.0	28.2
Average	**16.2**	**55.3**	**2.0**	**26.5**
% Rel.St.Dev.	*0.6*	*0.7*	*141.4*	*8.9*
Z8P S2_3_inj.1	14.8	54.6	4.6	25.9
Z8P S2_3_inj.2	15.2	55.9	0.0	28.8
Average	**15.0**	**55.3**	**2.3**	**27.4**
% Rel.St.Dev.	*1.9*	*1.7*	*141.4*	*7.6*
Average: 6 values	**15.5**	**55.3**	**2.2**	27.0
% Rel.St.Dev.	*4.1*	*0.0*	*8.4*	**17.3**
Z8P S3_1_inj.1	16.7	52.8	4.7	25.7
Z8P S3_1_inj.2	17.0	53.5	0.0	29.5
Average	**16.8**	**53.2**	**2.4**	**27.6**
% Rel.St.Dev.	*0.9*	*0.9*	*141.4*	*9.7*
Z8P S3_2_inj.1	16.4	52.0	4.8	26.8
Z8P S3_2_inj.2	17.4	55.4	0.0	27.3
Average	**16.9**	**53.7**	**2.4**	**27.0**
% Rel.St.Dev.	*3.9*	*4.5*	*141.4*	*1.3*
Z8P S3_3_inj.1	16.7	53.1	5.0	25.3
Z8P S3_3_inj.2	17.1	54.6	0.0	28.3
Average	**16.9**	**53.8**	**2.5**	**26.8**
% Rel.St.Dev.	*1.8*	*2.0*	*141.4*	*7.9*
Average: 6 values	**16.9**	**53.6**	**2.4**	27.2
% Rel.St.Dev.	*0.2*	*0.6*	*2.5*	**17.5**
Ave. 3 Z8Psamples	**15.31**	**55.18**	**2.31**	**22.67**
% Rel.St.Dev.	*10.8*	*2.9*	*4.5*	*33.7*

$$\% \text{ Analyte g/g Z8P} = 100 * \frac{PA_{\text{Analyte in Z8P sol.}} * \text{conc. Std sol. (mg}_{\text{Analyte}}\text{/mL)} * 1000 \text{ mg/g (Z8P)}}{PA_{\text{Analyte in Std sol.}} * \text{conc Std solution (mg}_{\text{Z8P}}\text{/mL)} * 1000 \text{ mg/g (Analyte)}}$$

PA stand for Peak Area

Table S7 *Calculation of the concentration of 2-min, i2ac, 1-AP and Zn^{2+} as mmol reactant /g Z8P.*

MW (g/mol)	82.1	96.09	217.27	65.38	
**Sample**	2-mim	i2ca	1-AP	Estimated Zn^{2+}	Imidazole/ Zn^{2+} ratio
	mmol/g Z8P	mmol/g Z8P	mmol/g Z8P	mmol/g Z8P	expected=2.0
Z8P S1_1_inj.1	1.66	5.92	0.22	3.79	2.00
Z8P S1_1_inj.2	1.68	5.98	0.22	3.67	2.08
Average	**1.67**	**5.95**	**0.22**	**3.73**	**2.04**
% Rel.St.Dev.	*0.7*	*0.6*	*1.6*	*2.2*	*2.8*
Z8P S1_2_inj.1	1.63	5.80	0.21	4.04	1.84
Z8P S1_2_inj.2	1.66	5.95	0.22	3.74	2.04
Average	**1.64**	**5.88**	**0.21**	**3.89**	**1.94**
% Rel.St.Dev.	*1.4*	*1.8*	*3.7*	*5.4*	*7.1*
Z8P S1_3_inj.1	1.62	5.80	0.22	4.02	1.84
Z8P S1_3_inj.1	1.67	5.96	0.22	3.72	2.05
Average	**1.65**	**5.88**	**0.22**	**3.87**	**1.95**
% Rel.St.Dev.	*1.9*	*1.9*	*1.6*	*5.6*	*7.5*
Average: 6 values	**1.65**	**5.90**	**0.22**	**3.83**	**1.98**
St.Dev.	*1.3*	*1.4*	*2.3*	*4.2*	*5.4*
Z8P S2_1_inj.1	1.82	5.63	0.21	4.03	1.85
Z8P S2_1_inj.2	1.89	5.88	0.22	3.55	2.19
Average	**1.85**	**5.76**	**0.22**	**3.79**	**2.02**
% Rel.St.Dev.	*2.8*	*3.0*	*1.9*	*8.9*	*11.8*
Z8P S2_2_inj.1	1.97	5.73	0.18	3.80	2.03
Z8P S2_2_inj.2	1.98	5.78	0.19	3.67	2.12
Average	**1.98**	**5.75**	**0.19**	**3.73**	**2.07**
% Rel.St.Dev.	*0.6*	*0.7*	*3.3*	*2.4*	*3.0*
Z8P S2_3_inj.1	1.81	5.68	0.21	3.96	1.89
Z8P S2_3_inj.1	1.85	5.82	0.22	3.68	2.08
Average	**1.83**	**5.75**	**0.22**	**3.82**	**1.99**
% Rel.St.Dev.	*1.9*	*1.7*	*2.1*	*5.2*	*6.9*
Average: 6 values	**1.89**	**5.75**	**0.21**	**3.78**	**2.03**
% Rel.St.Dev.	*4.0*	*1.6*	*7.4*	*4.9*	*6.5*
Z8P S3_1_inj.1	2.04	5.50	0.22	3.93	1.92
Z8P S3_1_inj.2	2.07	5.57	0.22	3.78	2.02
Average	**2.05**	**5.53**	**0.22**	**3.85**	**1.97**
St.Dev.	*0.9*	*0.9*	*1.3*	*2.9*	*3.8*
Z8P S3_2_inj.1	2.00	5.41	0.22	4.10	1.81
Z8P S3_2_inj.2	2.12	5.76	0.23	3.41	2.31
Average	**2.06**	**5.59**	**0.22**	**3.76**	**2.06**
% Rel.St.Dev.	*3.9*	*4.5*	*2.1*	*12.8*	*17.1*
Z8P S3_3_inj.1	2.03	5.52	0.23	3.87	1.95
Z8P S3_3_inj.1	2.08	5.68	0.24	3.55	2.19
Average	**2.06**	**5.60**	**0.23**	**3.71**	**2.07**
% Rel.St.Dev.	*1.8*	*2.0*	*2.2*	*6.1*	*8.1*
Average of 6	**2.06**	**5.57**	**0.23**	**3.77**	**2.03**
% Rel.St.Dev.	*2.0*	*2.3*	*2.9*	*6.7*	*9.1*
Ave.3 Z8P samples	**1.87**	**5.74**	**0.22**	**3.80**	**2.01**
% Rel.St.Dev.	*10.8*	*2.9*	*4.3*	*0.8*	*1.5*

$$\text{Analyte mmol/g Z8P} = \frac{PA_{\text{Analyte in Z8P sol.}} * \text{conc. Std sol. (mg}_{\text{Analyte}}\text{/mL)} * 1000 \text{ mg/g (Z8P)} * 1000 \text{ mmol/mol (Analyte)}}{PA_{\text{Analyte in Std sol.}} * \text{conc Std solution (mg}_{\text{Z8P}}\text{/mL)} * 1000 \text{ mg/g (Analyte)} * \text{MW g/mol (Analyte)}}$$

PA stand for Peak Area

5.3 Quantification of Zn^{2+} content on Z8P by ICP-OES

ICP-OES (inductively coupled plasma atomic emission spectroscopy) was used in order to obtain the quantitative content of Zn^{2+} in ZIF samples. ZIF must undergo a wet digestion procedure before the samples can be analysed by ICP-OES.

5.3.1 Suitable disintegration procedure for Z8P analyses by ICP-OES

The disintegration procedure is performed as follows. The samples were divided into four synthetic batches. The solution for each batch was independently prepared in duplicate and each solution was measured five times by ICP-OES.

1. Around 1 to 3 mg of each sample of Z8P was weighted
2. The sample was dissolved in 2 mL of HNO_3 30 %v/v. Concentrated HNO_3, previously purified by sub-boiling in a PFA device.
3. The disintegrated ZIF sample in concentrated HNO_3 was diluted with ultrapure deionized water to a volume of 10 mL and mixed well.

Note: When weighting the samples for ICP quantification, the weighted amounts were very small (1,115 mg and 1,118mg for sample 1; 2.863 mg and 2,865 for sample 2; 0.76 mg and 0.78 mg sample 3) and they were dissolved it to 10 ml as final volume. This dilution magnifies any error that is carried from the whole process.

ICP determination of a Zn^{2+} in the samples was performed against an external standard solution of a Zn^2 which is directly traceable to the NIST SRM 3169 (Certipur© Certified Reference Material, Merck, Germany).

5.3.2 ICP-OES parameter for analysis of disintegrated Z8P

The operating conditions for the ICP-OES (Agilent 5110 VDV) are listed on Table S8. The quantitation of Zn^{2+} in samples are summarized in Table S9.

Table S8 Operational conditions for the ICP OES analysis

Parameter	Operating condition
RF power (kW)	1.2
Auxiliary gas flow rate ($L\cdot min^{-1}$)	1.00
Plasma gas flow rate ($L\cdot min^{-1}$)	12.00
Nebulizer gas flow rate ($L\cdot min^{-1}$)	0.70
Viewing height (mm)	8
Pump speed (rpm)	15
Stabilization time (s)	30
Integration time (s)	5
Replicates	5
Viewing mode	Radial
Wavelength (nm)	202.548 (Zn II)

5.3.3 ICP Determination of Zn^{2+}; Results

The results of the ICP determination of the Zn content are as shown on Table S9. It reinforces the results of the composition obtained via HPLC on Fig. S14.

Table S9 Results for zinc determination in Z8P by an ICP-OES method. Uncertainties were reported as the combined standard deviation from two weighing and five repetitions

Z8P Sample	Zn	RSD *	Zn^{2+} (g/g ZIF)		Zn (µmol/g ZIF)	
	w/w %	%	(gZn^{2+}/g ZIF)	St.Dev	(µmol/g ZIF)	St.Dev
A	20.8	0.9	0.208	0.009	3.181	0.138
B	18.2	0.98	0.182	0.0098	2.784	0.150
C	19.5	0.69	0.195	0.0069	2.983	0.106
D	16.6	3.59	0.166	0.0359	2.539	0.549

5.3.4 Summary of HPLC and ICP-OES results

Table S10 Tabulation of reactants added to form target Z8P versus concentration of bound reactants in target Z8P determined by HPLC, both referred to g target Z8P. This table is illustrated on Fig. S14

**Z8P S1**	**MW**	**Added reactants to synthesise target Z8P**		
**Reagent**	**(g/mol)**	**mg added**	mmols Reactant per g target Z8P	% mol/mol Reactants per g target Z8P
**2-mim**	82.1	40.400	16.26	**37.8**
**i2ca**	96.09	47.280	16.26	**37.7**
**1ap**	217.27	16.080	2.45	**5.7**
**Zn^{2+}**	65.38	16.043	8.11	**18.8**
**Total amount reactants added (mg)**		119.803	43.068	**100.0**
**Weight of target Z8P after wash(mg)**		30.267		
Zn^{2+} = 100-%ligands				

**Z8P S1**	**MW**	**Bound reactants in target Z8P** Composition obtained by HPLC		
	**(g/mol)**	g Reactant per g target Z8P	mmols Reactant per g target Z8P	% mol/mol Reactants per g target Z8P
**2-mim**	82.1	15.31	1.87	**16.05**
**$i2ca_{free}$**	96.09	55.18	5.74	**49.43**
**Imine**	217.27	4.69	0.22	**1.86**
**Zn^{2+}**	65.38	24.81	3.80	**32.66**
**Total**			11.619	100.00
[imine] = [1-AP] = [$i2ca_{bound}$]		[$i2ca_{free}$] = [$i2ca_{total}$] - [1-AP]		

.

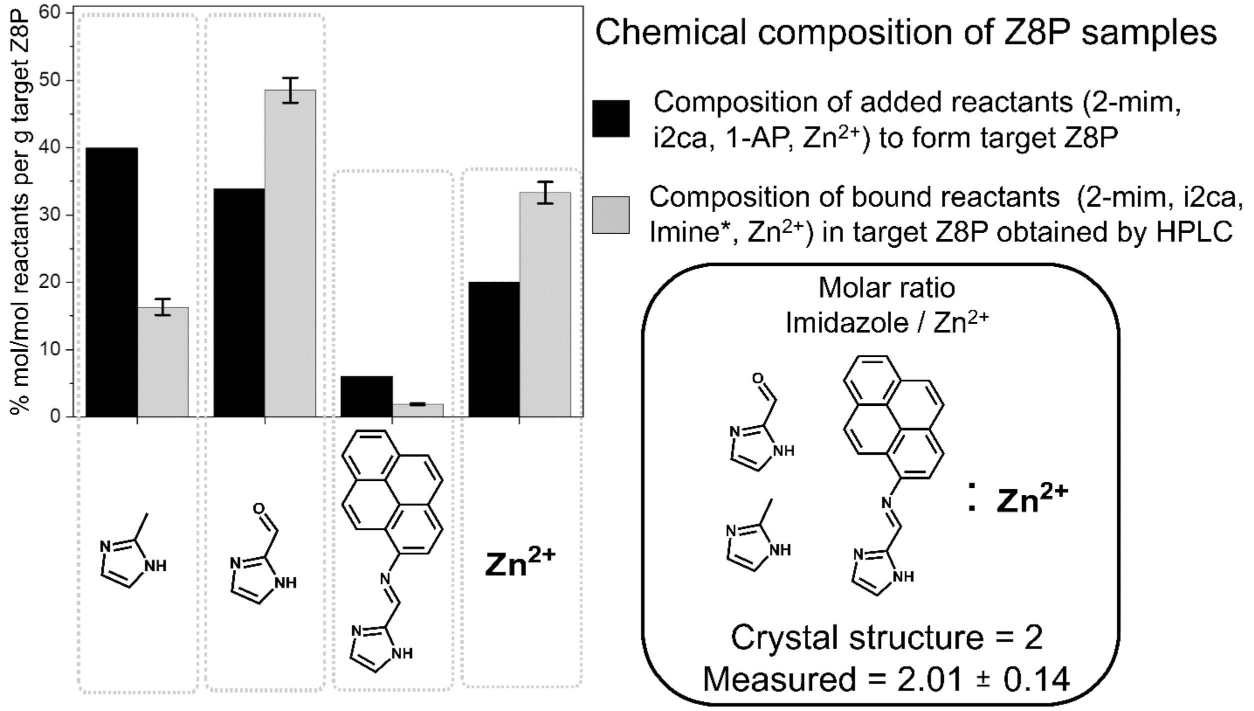

* **Note 1**: We assume that 1 eq. of the imine is formed quantitatively from 1 eq. of 1-ap added, reacting with 1 eq. of added i2ca

Note 2: 2-mim needs to be added in excess to form controlled nanosized Z8P (50-200 nm)

Fig. S14 Graphical representation of the results of the chemical composition of Z8P determined by HPLC expressed in %mol/mol reactants per g of target Z8P as calculated on Table S10. After reaction of 120 mg of reactants (2-mem, i2ac, 1-AP and Zn2+) and extensive washing of the excess of reactants and intensive drying (5 mbar, 70 °C for 2 days), an average of 30 mg of target Z8P is obtained, resulting on an approximate yield of 25% in mass. Zn^{2+} values calculated from the HPLC data was obtained by deducting the total percentage (%g/g Z8P) for 2-mim, i2ca and 1-AP from the total (100%), assuming all the non-accounted for mass on the Z8P comes from Zn^{2+}. The calculated ratio of the structural constituents for the ZIF is compared to the expected levels based on: the crystal structure of the corresponding Z8P which are stoichiometrically formed by 2 imidazole bridging species per one Zn^{2+}metallic core. Using the concentration values from Table S7 and dividing the total mols for i2ca and 2mim by the mols of Zn^{2+}, we obtain a value of 2.01 very close to the stoichiometric composition of the crystal.

5.4 Powder X-Ray diffractograms for the ZIFs, both conjugated and unconjugated

Both conjugated and unconjugated ZIFs were analysed by X-ray powder diffraction with Bragg-Brentano symmetry to determine the crystal structure of the obtained ZIFs. The parameters for all diffractograms are: 2θ 5-50°; step size 0.02°; integration time 6 sec.

These measurements demonstrate that the ZIFs conserve its characteristic SOD (sodalite) crystalline structure even after the conjugation process has taken place.

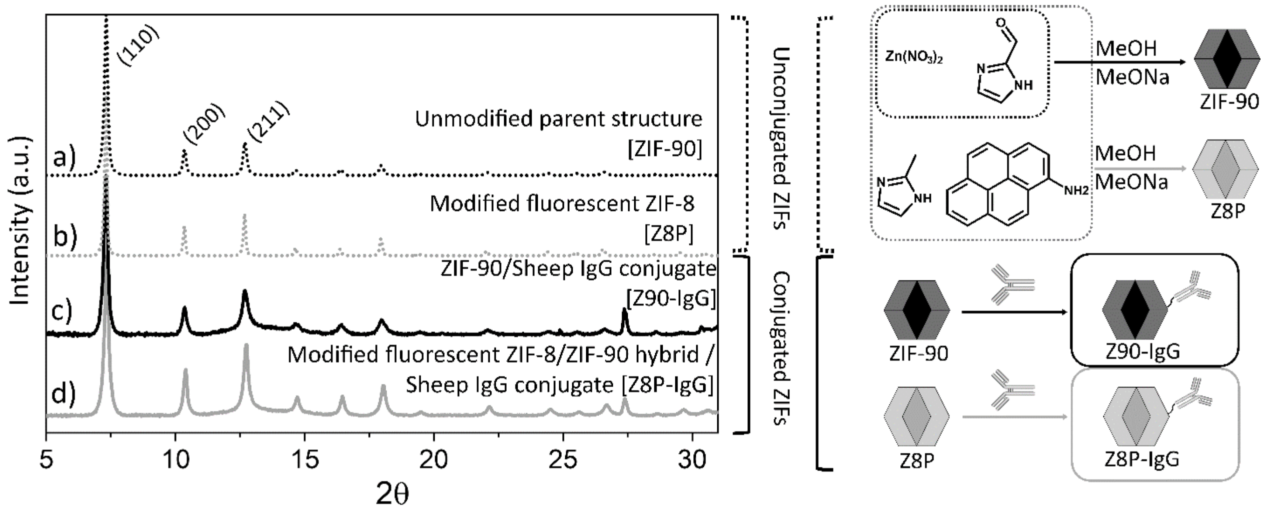

Fig. S15 Powder diffractograms for ZIF-90 (a), the modified fluorescent ZIF-8/ZIF-90 hybrid [Z8P] (b), the ZIF-90−IgG conjugate [Z90−IgG] (c) and the modified fluorescent ZIF-8/ZIF-90 hybrid-IgG conjugate [Z8P−IgG] (d), all measured in Bragg-Brentano symmetry. It can be seen how all ZIFs have the reported sodalite structure of ZIF-8, and how the conjugation process does not affect the crystalline integrity of the ZIFs.

5.4.1 Preparation of PXRD sample of non-conjugated ZIFs

To prepare the non-conjugated ZIF samples, each ZIF powder was softly ground in an agate mortar with an agate pestle, then placed on a Bragg-Brentano PVC sample holder with an opening of 0.8 cm in diameter. The cavity of the sample holder was filled with the powder and with the help of a glass slide, the top surface of the sample was compacted flat and flash with the top surface of the holder.

5.4.2 Preparation of PXRD sample of conjugated ZIFs

For the conjugated ZIFs, 400 µL of a freshly prepared ZIF solution (SI-6.1) from each conjugate (a total mass of 0.3 mg for each measurement), was transferred to an Eppendorf tube, centrifuged and all excess supernatant to 50 µL carefully removed with a pipette. This remaining 50 µL suspension was placed on a zero-background silicon monocrystalline (SIOV) sample holder using a 100 µL pipette. The measurement was started immediately, with the sample still wet.

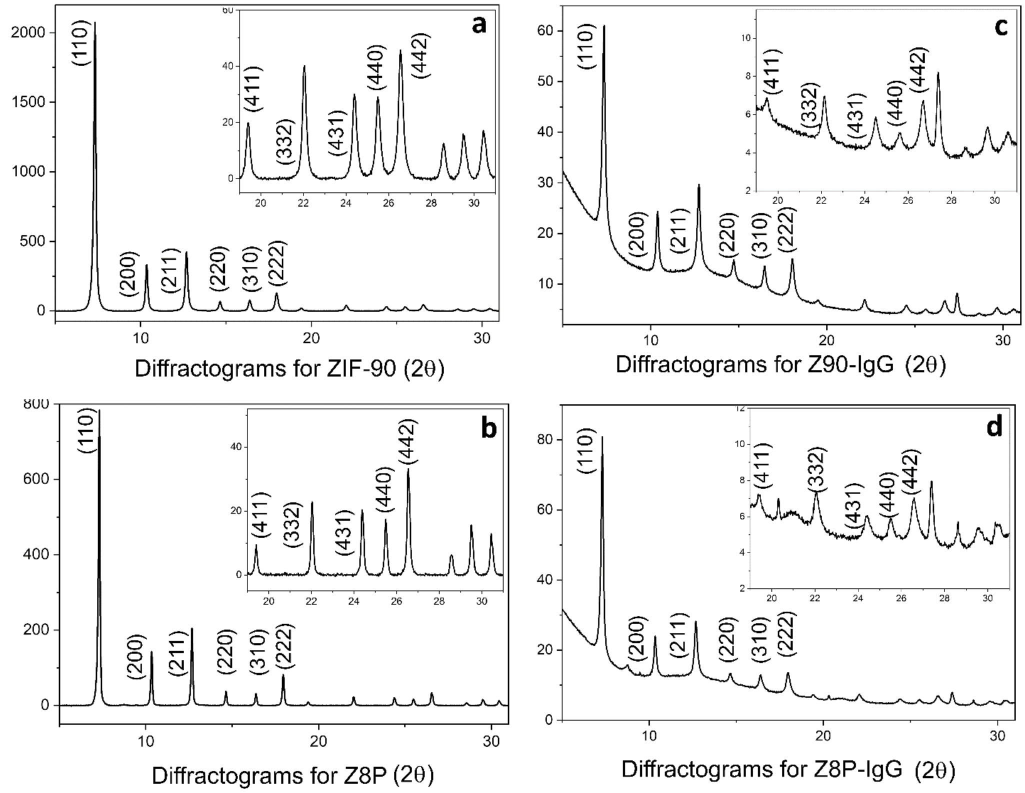

Fig. S16 Powder diffractograms for ZIF-90 (a), Z8P (b), Z90-IgG conjugate (c) and Z8P-IgG conjugate (d). All samples were measured in Bragg-Brentano symmetry. The position of all diffractions, as well as the relative intensity between a), b), c) and d) match the expected sodalite structure, showing that they all were isostructural. The most relevant Miller indices are shown[17].

6 Conjugation of ZIFs with sheep IgG

In this section we present the two-step protocol to covalently bind the free amino functionality of the lysine residue (-(CH_2)$_4$-NH_2 of the antibody tested to the free aldehyde functionality part of the imidazole 2-carboxaldehyde, included on the ZIFs of interest (ZIF-90 and the fluorescently modified ZIF-8/ZIF-90 hybrid, Z8P).

In SI-9 we discuss how we demonstrate that this binding has been successfully achieved.

6.1 General procedure scheme

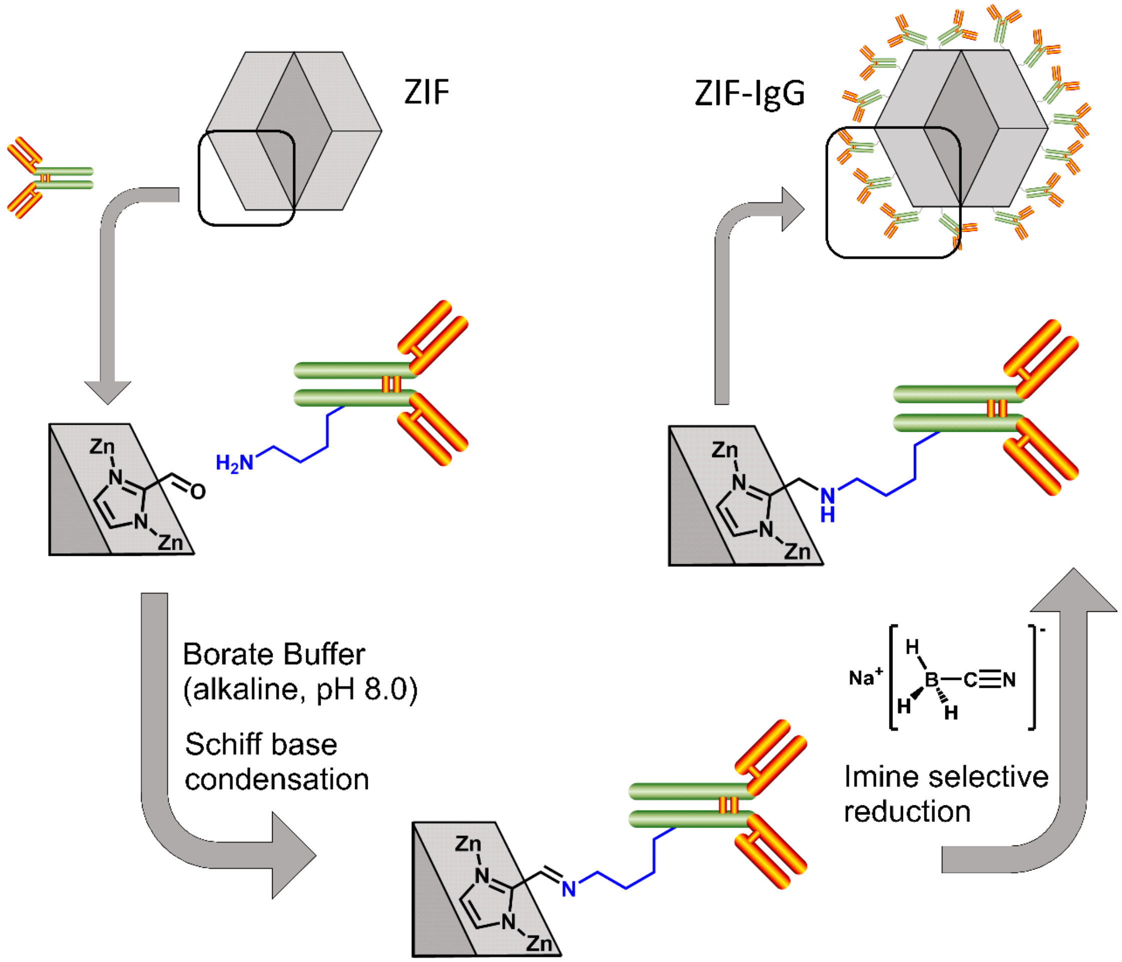

Fig. S17 Schematic representation of the conjugation reaction between the ZIFs and the antibodies. In the first step the dispersed particles were mixed in solution with the antibodies. The free amino functionalities from lysine residues side chains in of the antibodies can react with the aldehyde functionality on the ZIF to form an imine bond. Sodium cyanoborohydride was added in the second step to selectively reduce the imine bond to an amine, rendering the conjugation irreversible under assay conditions.

6.1.1.1 Conjugation of ZIF-90 and Z8P with anti-mouse IgG protocol

1) Weight 1.5 mg of the ZIF (**ZIF-90** or **Z8P**) on a 5-figure balance and transfer it to a small glass vial with a plastic cap.
2) Add 1.5 mL of a borate buffer solution (see SI-2.2.1), measured with a 2.5 mL pipette.
3) Seal the glass vial with the cap, and sonicate for 15 min.

4) Add 50 µL of a 2 mg/mL solution of **sheep anti-mouse IgG** (see SI-1.6) to this dispersion without any previous purification step using a 100 µL pipette.
5) Add a magnetic stirrer bar, seal the glass vial, and stir the mixture for 15 min at room temperature.
6) Add 50 µL of a commercially prepared aqueous solution of **Na[BH_3(CN)]** (5 M) in 1 M NaOH using a 200 µL pipette and allow to react for an additional 4 hours at room temperature.

Warning: Exercise extreme care when handling Cyanoborohydride. Always wear gloves, glasses and work in a properly enclosed fume hood.

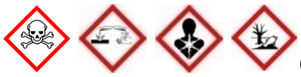
GHS05, GHS06, GHS08, GHS09

6.1.1.2 Protocol for washing the freshly prepared ZIF-90-IgG or Z8P-IgG

The next steps were designed to remove any anti-mouse IgG not covalently bonded to ZIF-90 or Z8P. The washing protocol of this conjugation reaction is as follows:

1) Transfer the 1.5 mL suspension to a 2 mL Eppendorf tube with the help of a 1 mL pipette and centrifuge it at 15000 rpm for 5 min.
2) Carefully remove the supernatant taking care not to disturb the solid residue and transfer it to a common container labelled as “Wash 1”. This wash is reserved for later use, in case the ELISA experiment fails suggesting unsuccessful binding between the IgG and the ZIF. “Wash 1” will help to investigate if the free IgG in the wash was active or inactive. “Wash 1” is disposed of, after successful completion of ELISA experiments

Warning: The very first supernatant of “Wash 1” contain cyanide derivatives. Extreme care must be exercised when dealing with and disposing of “Wash 1”.

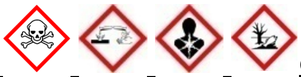
GHS05, GHS06, GHS08, GHS09

3) Add 1 mL of borate buffer to the Eppendorf tube to wash the solid residue.
4) Sonicate the suspension for 2 min to ensure full dispersion of the solid particles
5) Centrifuge the dispersion at 15000 rpm for 5 min
6) Remove the supernatant carefully avoiding contact with the solid residue and transfer to a separately container labelled as “Wash 2”. Wash-2. is also reserved for later use, in case the ELISA experiment fails suggesting unsuccessful binding between the IgG and the ZIF. “Wash 2” will further help to investigate if the free IgG in the wash was active or inactive. “Wash 2” is disposed of, after successful completion of ELISA experiments
7) Add 1 mL of TRIS buffer (see SI-2.2.3) to the residue
8) Redisperse the solid residue by vigorous agitation, and centrifuge at 15000 rpm for 5 min
9) Carefully remove this TRIS buffer supernatant and discard it

10) Repeat **steps 7-9** three more times to wash out any IgG not properly conjugated to the ZIF
11) Perform **steps 7-9** two additional times, this time with borate buffer
12) Disperse the properly washed solid residue containing the ZIF conjugate in 2 mL of borate buffer. This is the **ZIF-IgG stock solution** (0.75 mg/ml) used in all the ELISA, LFIA, LC/MS and PXRD characterization.

Addendum: The amount of washing steps was developed so that only the covalently bound antibody remains on the ZIF particles.

7 Studies on conjugated antibody activity

In this section we study the residual activity of the antibodies bound to the nanoparticulate ZIFs. We show the viability of the ZIFs as fluorescent bio labels on a series of non-competitive ELISA experiments.

7.1 Model non-competitive indirect ELISA

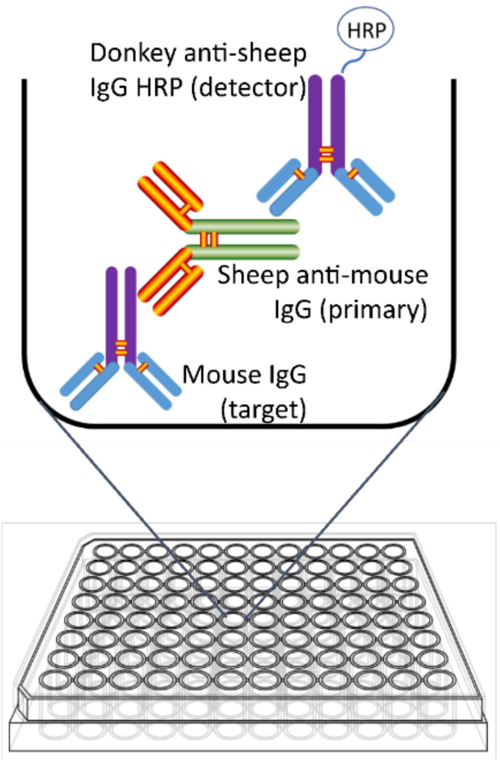

Fig. S18 Schematic representation of the general non-competitive indirect ELISA procedure developed to study the antibody activity of the conjugate. The electrostatically charged surface of the high-binding microtiter plates (MTP) was coated with the mouse IgG and incubated on a plate shaker. After washing, the remaining binding sites on the MTP were blocked with casein to avoid any passive adsorption of the conjugates to the surface of the MTP ("non-specific binding"), which would lead to false positive results. After another incubation, the plates were washed again and dilutions of an anti-mouse IgG antibody (or a modified system including anti-mouse IgG) at various concentrations were added and the MTP incubated for 18 hours on a plate shaker. After an additional washing step, a solution with the HRP-labelled detection antibody (anti-sheep IgG) was added and incubated for 1 hour on a microplate shaker. After a final washing step, the substrate 3,3',5,5'-tetramethylbenzidine (TMB) was added to each well and incubated for 15 min on a microplate shaker, then 100 µL of 1 M sulphuric acid was added to each well and the optical density (OD) was measured. The response that should be obtained, should the system response correctly, would be a characteristic sigmoidal calibration curves, obtained by fitting a four-parameter logistic function to the data points.

The basic model to assess antibody activity by a non-competitive ELISA methodology is depicted in Fig. S18. It is a layered sandwich system which has a base formed by:

1. Target: **mouse IgG**
2. Primary [selective antibody]: **sheep anti-mouse IgG**
3. Detector: **HRP labelled donkey anti-sheep IgG**

This sandwich system is used to characterize the activity response of the primary, analyte-selective antibody. This system will be later modified by switching the primary antibody for our synthesised **ZIF-IgG**s.

The system will be quantified via HRP (Horse Radish Peroxidase) redox reaction driven TMB colour development, in which HRP triggers the conversion of TMB (transparent) to its oxidized coloured product (first intense blue, then bright yellow after the addition of 1M sulfuric acid)[12]; the coloration is proportional to the amount of HRP present on the system which, in turn, is proportional to the amount of primary present on the system.

The calibrating solutions were prepared by making serial dilutions from a stock solution of the antibody species to be assessed (**anti-mouse IgG** or **ZIF-IgG conjugate**). The rest of the species taking part on the experiment should be maintained at constant concentrations. In order to compare accurately the amounts of soluble antibodies, each well was processed under the identical conditions, and 4 replicates of each measurement was performed.

7.2 Protocol of the non-competitive indirect ELISA for the anti-sheep IgG primary

In this version of the protocol we assess the activity of our base ELISA system [**mouse IgG** (target) **/ sheep anti-mouse IgG** (from sheep, primary, selective antibody) **/ anti-sheep IgG-HRP** (from donkey, enzyme-labelled detector antibody)].

The standard curve was prepared by making serial dilutions of one known concentration of the primary (**sheep anti-mouse IgG**) across a range of concentrations (0.25-750 ng/ml), while keeping every other parameter of the system constant. Each well was treated under the same conditions, and each measurement was done in quadruplicate.

For details on the antibodies see SI-1.6.

The protocol is performed at follows:

1) Coat a 96 well transparent high-binding microtiter plate (flat-bottom, Nunc MaxiSorp™) with the target mouse IgG (**Capture antibody solution,** SI-7.2.1) by transferring 200 µL of the capture antibody solution into each well using a 200 µL multichannel electronic pipette
2) Seal the plates with Parafilm® to prevent evaporation
3) Incubate for 4 h on a microplate shaker
4) Wash the wells of the microtiter plates with a 3-repetition cycle automatized microtiter washer using a PBS-based washing buffer (SI-2.2.6).
5) Transfer 200 µL of the casein solution (**Casein blocking solution,** SI-7.2.2) into each well using a 200 µL multichannel electronic pipette. This will ensure that the remaining binding sites on the MTP are blocked
6) Seal the plates with Parafilm® to prevent evaporation

7) Incubate for 1 h on a microplate shaker
8) Wash the plates again after this incubation, as in point 4
9) Add 200 µL of the sheep anti-mouse IgG solutions (**sheep anti-mouse IgG solutions in borate buffer,** SI-7.2.3) to the corresponding wells using a 200 µL pipette
10) Seal the plates with Parafilm® to prevent evaporation
11) Incubate for 18 hours on a microplate shaker
12) Wash the plates again after this incubation, as in point 4
13) Add 200 µL of the anti-sheep HRP-labelled IgG antibody (**HRP-labelled detection antibody,** SI-7.2.4) to each well using a 200 µL multichannel electronic pipette
14) Seal the plates with Parafilm® to prevent evaporation
15) Incubate for 1 hour on a microplate shaker
16) Wash the plates again after this incubation, as in point 4
17) Add 200 µL of the TMB solution (**TMB-based substrate solution,** SI-7.2.5) to each well using a 200 µL multichannel electronic pipette and incubate for 15 min on a microplate shaker. Each wells solution will turn from transparent to blue in colour
18) Add 100 µL of the stop solution (1 M sulphuric acid) to each well. The colour will change from blue to yellow
19) Measure the optical density (OD) of the wells (SI-3.8)

Characteristic sigmoidal calibration curves were obtained by fitting a four-parameter logistic function in Origin to the data points obtained from OD measurements. The sigmoidal shape of Standard calibration curves is expected when the binding occurs via intact antibody binding sites and not, e.g., due to non-specific binding. The calibration standard solutions were prepared by making serial dilutions from a stock solution of the antibody species to be assessed (**anti-mouse IgG**), while keeping the rest of the solution constituents at constant concentrations. In order to compare the amounts of soluble antibodies, each well was processed under the same conditions, and 4 replicates of each measurement were performed in.

7.2.1 Preparation of Capture antibody solution

1) Transfer 20 mL of borate buffer (see SI-2.2.1) to a long glass vial with a plastic cap using a 10 mL pipette
2) Transfer 1.5 µL of **mouse IgG** using a 5 µL pipette. Wash the tip by aspirating/dispensing the solution several times
3) Seal the glass vial and homogenize the mixture using a mechanical shaker

7.2.2 Preparation of Casein blocking solution

1) Weigh 20 mg of **casein** and transfer to a long glass vial with a plastic cap
2) Add 20 mL of PBS buffer (see SI-2.2.2) using a 10 mL pipette
3) Seal the glass vial and homogenize the mixture using a mechanical shaker

7.2.3 Preparation of sheep anti-mouse IgG solutions in borate buffer

7.2.3.1 Preparation of 3 stock solutions

Prepare in total 3 stock solutions, each to a total volume of 1 mL. Stock solution **MS1** was prepared from the original stock solution of the **sheep anti-mouse IgG** (2 mg/mL). Stock solution **MS2** was prepared by dilution from **MS1**; Stock solution **MS3** was prepared by dilution from **MS2.**

MS1 was prepared as follows:

1) Transfer 1 mL of borate buffer inside a 1.5 mL Eppendorf tube
2) Remove 25 µL of the buffer using a 100 µL pipette
3) Transfer 25 µL of the original stock solution of the **sheep anti-mouse IgG** with a new tip on the micropipette. Wash the tip by aspirating/dispensing the solution several times
4) Seal the Eppendorf tube and homogenise the solution using a mechanical shaker

MS2 was prepared as follows:

1) Transfer 1 mL of borate buffer inside a new 1.5 mL Eppendorf tube
2) Remove 100 µL of borate buffer with a 100 µL pipette
3) Transfer 100 µL of the **MS1** stock solution with a new tip on the micropipette. Wash the tip by aspirating/dispensing the solution several times
4) Seal the Eppendorf tube and homogenise the solution using a mechanical shaker

MS3 was prepared as described for **MS2** but using **MS2** as the stock solution

The concentration for the MS solutions will be:

MS1 = 50 µg/mL;
MS2 = 5 µg/mL;
MS3 = 0.5 µg/mL;

7.2.3.2 Preparation of the dilution series for assessing antibody activity of anti-mouse IgG

We prepare 11 new solutions from these four stock solutions (**MS1, MS2** and **MS3**) to varying concentrations for **sheep anti-mouse IgG**.

The procedure **for each dilution series stock (number #)** is performed as follows:

1) Transfer 1 mL of borate buffer into a new Eppendorf tube.
2) Using the suitable micropipette, remove from the Eppendorf tube a volume of borate buffer equivalent to the corresponding cell on the column named “aliquot volume from MS stock solution” as listed on Table S11.
3) Using the same micropipette, transfer the aliquot volume corresponding to the exact same cell as on step 3 from Table S11, with a new tip on the micropipette. Wash the tip by aspirating/dispensing the solution several times
4) Seal the Eppendorf tube and homogenise the solution using a mechanical shaker

WARNING; In order to keep the antibodies stable during the whole process all solutions are kept on ice.

*Table S11 Table for the preparation of the solutions for the sheep anti-mouse IgG solutions in borate buffer. The * represent the volume of buffer to be replaced by the stock solutions (MS1, MS2, MS3 and MS4)*

Dilution series stock #	MS Stock solution used	aliquot volume from MS stock solution* (µL)	Concentration in stock solution (ng/mL)
1	MS1	15	750.00
2	MS1	10	500.00
3	MS1	5	250.00
4	MS1	2.5	125.00
5	MS2	10	50.00
6	MS2	5	25.00
7	MS2	2	10.00
8	MS3	10	5.00
9	MS3	5	2.50
10	MS3	2	1.00
11	MS3	0.5	0.25

7.2.4 Preparation of the solution of HRP-labelled detection antibody

1) Transfer 10 mL of PBS buffer (see SI-2.2.2) to a long glass vial with a plastic cap using a 10 mL pipette
2) Transfer 1.5 µL of **anti-sheep IgG HRP labelled** using a 5 µL pipette. Wash the tip by aspirating/dispensing the solution several times.
3) Close the glass vial, and homogenize the mixture using a mechanical shaker

7.2.5 Preparation of the solution of TMB-based substrate

1) Transfer 22 mL of citrate buffer (see SI-2.2.4) to a long glass vial with a plastic cap using a 10 mL pipette

WARNING: each pipette tip was used only once, since the citrate buffer was cold after being kept in the fridge for stability purposes. Using the same pipette tip for transferring all 22 ml can confer significant error, as the shape of the plastic tip becomes deformed.

2) Add 8.5 µL of H_2O_2 (30% v/v) using a 10 µL pipette
3) Add 550 µL of the **TMB** solution (SI-2.2.7) using a 1 mL pipette
4) Close the glass vial, and homogenize the mixture using a mechanical shaker

Table S12 Optical density (OD) values obtained in the non-competitive indirect ELISA of the anti-mouse IgG with the associated standard deviation. These values are displayed graphically in Fig. S19.

Initial concentration for sheep IgG in Borate Buffer with NaCl (ng/mL)	Optical Density (OD)	Standard Deviation (S.D.)
750	3.400	0.013
500	3.203	0.066
250	2.866	0.159
125	2.374	0.049
50	2.002	0.029
25	1.539	0.030
10	0.798	0.031
5	0.494	0.017
2.5	0.307	0.014
1	0.150	0.011
0.25	0.049	0.040

Table S13 Controls performed to check any cross reactivity in the indirect ELISA. All controls have been done with a very high concentration for all the compounds that could show cross-reactivity with the primary (anti-mouse IgG). Anti-sheep-HRP has some cross-reactivity against the mouse IgG deposited on the surface of the plate but this was not so strong as to interfere with the signal of the indirect ELISA. The ZIF-IgG conjugates, tested at very high concentrations, show a residual signal when no mouse IgG was present, thus showing non-interaction and potential false positives.

**Controls**					
	Concentration of Z8P−IgG conjugate (ng/mL)	Concentration of anti-sheep−HRP (ng/mL)	Concentration of Casein (ng/mL)	OD	S.D.
Mouse IgG + casein + anti-sheep IgG−HRP	0	200	1000	0.191	0.002
Casein + ZIF−IgG conjugate + anti-sheep IgG−HRP	1500	200	1000	0.085	0.007
	1125	200	1000	0.079	0.008
	750	200	1000	0.067	0.004
Casein + anti-sheep IgG−HRP	0	200	1000	0.057	0.003

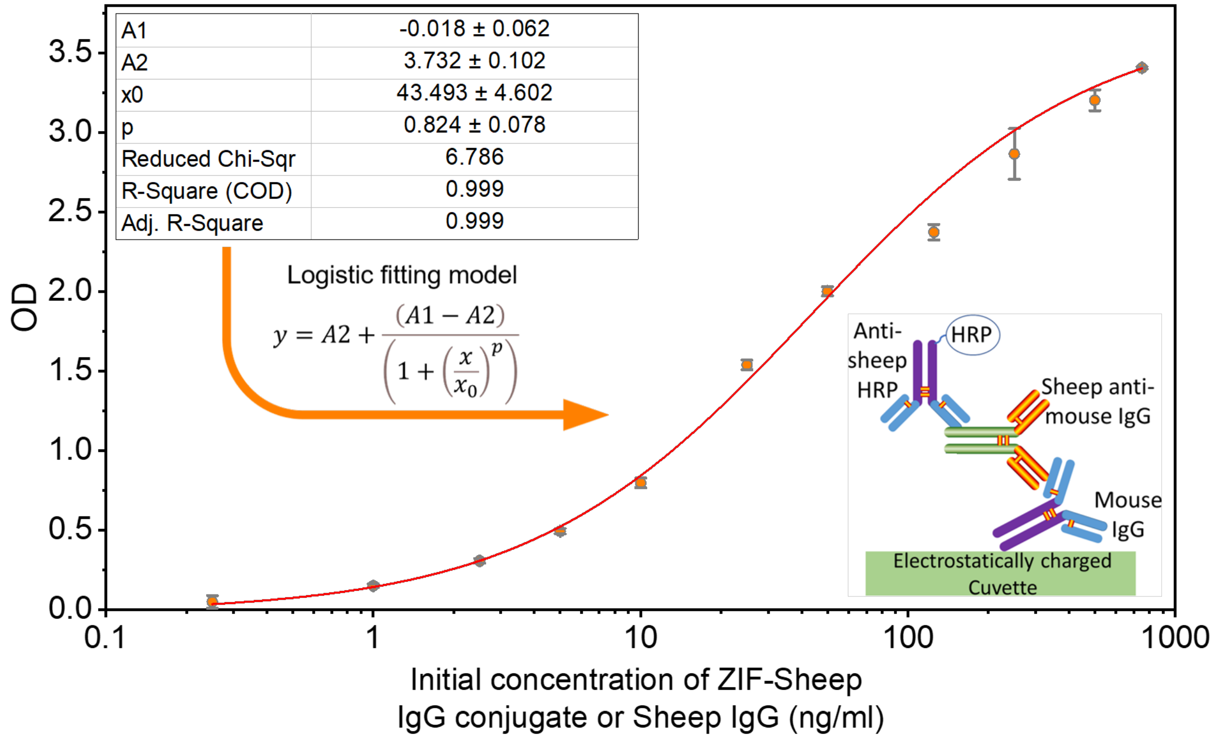

Fig. S19 Non-competitive indirect ELISA calibration curves for the system of interest. The optical density (OD) was plotted against the initial concentration for the sheep anti-mouse−IgG in ng/mL. Controls were performed to check if there was any cross-reactivity in the indirect ELISA (Table S13). The logistic function (4-parameter function) has been calculated which fits perfectly the binding events in immunoanalytical methods. Rodbard discovered around 1985 that these functions perfectly fit immunoassay data. The parameters are here named A1, A2, Xo and p. x is the analyte concentration, A1 is the maximum signal (upper asymptote of the curve), A2 is the signal background (lower asymptote of the curve, lowest point on "y-axis", Xo is the x coordinate of the inflection point ("test mid point") of the curve, in concentration units and p is a parameter that reflects the slope of the curve. HRP is Horseradish peroxidase.

***Conclusion**: The activity of our primary (sheep anti-mouse IgG) is tested and proven. This is a suitable base system to modify it including our ZIF-IgG conjugates s primaries.*

7.3 Protocol of the non-competitive indirect ELISA for the ZIF-IgG conjugates

In this version of the protocol we assess the residual activity of the antibodies bound to both our ZIF-IgG conjugates by a modified ELISA system [**mouse IgG** (target) **/ ZIF-IgG** (**Z8P-IgG** and **Z90-IgG**, primary, selective antibody) **/ anti-sheep IgG−HRP** (from donkey, enzyme-labelled detector antibody)].

The standard curve was prepared by making serial dilutions of one known concentration of the analyte (ZIF−IgG conjugates, both **Z90-IgG** and **Z8P-IgG**) across a range of concentrations (0-150 ng/ml), while keeping every other parameter of the system constant. Each well was treated under the same conditions, and each measurement was done in quadruplicate.

The protocol described in SI-7.2 was performed with the following modification:

Step 9 in SI-7.2 protocol: Add 200 µL of the ZIF-IgG conjugate solutions (**ZIF-IgG** solutions in borate buffer, SI-7.3.1) to the corresponding wells using a 200 µL pipette. Incubate for 18 hours.

7.3.1 Preparation of ZIF−IgG solutions in borate buffer:

*7.3.1.1 Preparation of 4 stock solutions of **ZIF-IgG***

Prepare in total 4 stock solutions, each to a total volume of 1 mL. Stock solution **MS1** was prepared from the initial stock solution of **Z8P-IgG** (0.75 mg/mL) and **Z90-IgG** (0.75 mg/mL); Stock solution **MS2** was prepared by dilution from **MS1**; Stock solution **MS3** was prepared by dilution from **MS2**; Stock solution **MS4** was prepared by dilution from **MS3.**

MS1 was prepared as follows:

1) Transfer 1 mL of borate buffer (see SI-2.2.1) inside a 1.5 mL Eppendorf tube
2) Remove 10 µL of the buffer using a 100 µL pipette
3) Transfer 10 µL of the original stock solution of the **ZIF-IgG** (**Z90-IgG** or **Z8P-IgG**) with a new tip on the micropipette. Wash the tip by aspirating/dispensing the solution several times
4) Seal the Eppendorf tube and homogenise the solution using a mechanical shaker

MS2 was prepared as follows:

1) Transfer 1 mL of borate buffer inside a new 1.5 mL Eppendorf tube
2) Remove 100 µL of borate buffer with a 100 µL pipette
3) Transfer 100 µL of the **MS1** stock solution with a new tip on the micropipette. Wash the tip by aspirating/dispensing the solution several times
4) Seal the Eppendorf tube and homogenise the solution using a mechanical shaker

MS3 was prepared as described for **MS2** but using **MS2** as the stock solution

MS4 was prepared as described for **MS2** but using **MS3** as the stock solution

The concentration for the MS solutions will be:

MS1 = 7.5 µg/mL;
MS2 = 0.75 µg/mL;
MS3 = 0.075 µg/mL;
MS4 = 0.0075 µg/mL.

7.3.1.2 Preparation of the dilution series for assessing antibody activity of ZIF-IgG

We prepare 23 new solutions from these four stock solutions (**MS1, MS2, MS3** and **MS4**) to varying concentrations for **ZIF-IgG** (both **Z90-IgG** and **Z8P-IgG**).

The procedure **for each dilution series stock (number #)** is performed as follows:

1) Transfer 1 mL of borate buffer into a new Eppendorf tube.
2) Using the suitable micropipette, remove from the Eppendorf tube a volume of borate buffer equivalent to the corresponding cell on the column named “aliquot volume from MS stock solution” as listed on Table S14.
3) Using the same micropipette, transfer the aliquot volume corresponding to the exact same cell as on step 3 from Table S14, with a new tip on the micropipette. Wash the tip by aspirating/dispensing the solution several times
4) Seal the Eppendorf tube and homogenise the solution using a mechanical shaker

WARNING; In order to keep the antibodies stable during the whole process all solutions are kept on ice.

*Table S14 Table for the preparation of the ZIF−IgG solutions in borate buffer. The * represent the volume of buffer to be replaced by the stock solutions (MS1, MS2, MS3 and MS4).*

Dilution series stock #	MS Stock solution used	aliquot volume from MS stock solution* (µL)	Concentration in stock solution (ng/mL)
1	MS1	20	150
2	MS1	15	112.5
3	MS1	10	75
4	MS1	8	60
5	MS1	6	45
6	MS1	4	30
7	MS1	2	15
8	MS2	10	7.5
9	MS2	8	6
10	MS2	6	4.5
11	MS2	4	3
12	MS2	2	1.5
13	MS3	10	0.75
14	MS3	9	0.675
15	MS3	6	0.45
16	MS3	4	0.3
17	MS3	2	0.15
18	MS4	10	0.075
19	MS4	8	0.06
20	MS4	6	0.045
21	MS4	4	0.03
22	MS4	2	0.015
23	MS4	0	0

Table S15 Optical Density (OD) values obtained on the non-competitive indirect ELISA for the ZIF conjugates.

**Z90-IgG**				**Z8P-IgG**		
Initial concentration for Z90−IgG in Borate Buffer with NaCl (ng/mL)	OD	S.D.		Initial concentration for Z8P−IgG in Borate Buffer with NaCl (ng/mL)	OD	S.D.
150.00	0.979	0.040		150.00	1.224	0.096
112.50	0.850	0.025		112.50	0.966	0.053
75.00	0.646	0.069		75.00	0.719	0.047
60.00	0.568	0.044		60.00	0.664	0.020
45.00	0.511	0.006		45.00	0.544	0.060
30.00	0.429	0.016		30.00	0.451	0.043
15.00	0.363	0.025		15.00	0.391	0.029
7.50	0.300	0.018		7.50	0.321	0.008
6.00	0.313	0.014		6.00	0.316	0.030
4.50	0.285	0.020		4.50	0.289	0.013
3.00	0.296	0.009		3.00	0.280	0.012
1.50	0.288	0.007		1.50	0.275	0.013
0.75	0.277	0.027		0.75	0.265	0.014
0.68	0.259	0.009		0.68	0.267	0.025
0.45	0.284	0.016		0.45	0.262	0.002
0.30	0.269	0.003		0.30	0.264	0.009
0.15	0.284	0.012		0.15	0.275	0.009
0.08	0.275	0.009		0.08	0.281	0.013
0.06	0.284	0.016		0.06	0.276	0.010
0.05	0.263	0.017		0.05	0.258	0.009
0.03	0.261	0.007		0.03	0.260	0.016
0.02	0.259	0.003		0.02	0.304	0.079
0.00	0.277	0.007		0.00	0.285	0.042

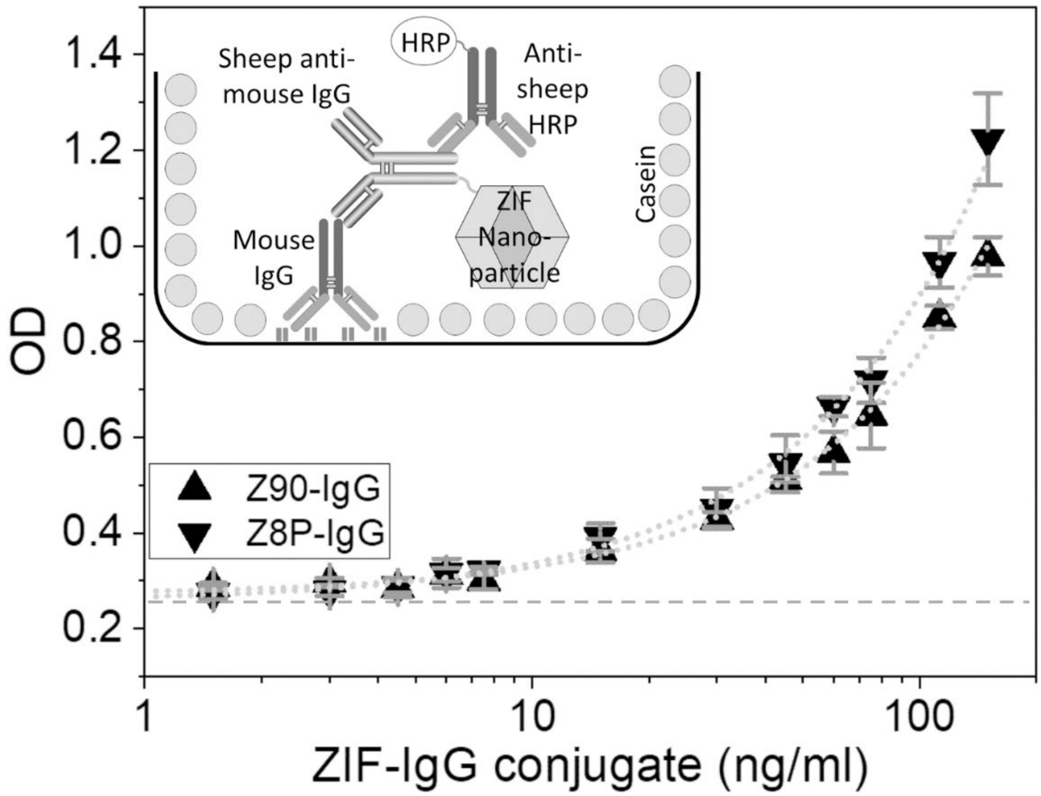

Fig. S20 Non-competitive indirect ELISA calibration curves for ZIF-IgG conjugates. The optical density (OD) was plotted against the initial concentration for the sheep anti-mouse-IgG in ng/mL (Table S15). Controls were performed to check if there was any cross-reactivity in the indirect ELISA. Binding with increasing concentrations is observed (no full sigmoidal curve recorded). OD for the blank (absence of conjugates) is indicated by the dotted horizontal line (OD = ca. 0.26). The LOD for Z90-IgG is 10 ng/ml and Z8P-IgG, 20 ng/ml. HRP is Horseradish peroxidase.

Conclusion: A similar response to the one we obtained on our previous ELISA system (SI-7.2) can be observe, proving that the antibodies bound to the ZIF conjugates retain their activity.

7.4 Protocol of the non-competitive ELISA for the antibody conjugates Z8P-IgG / non-competitive fluorescent ELISA tandem

To study the viability of our fluorescently modified ZIF-8/ZIF-90 hybrid as a fluorescent bio label in immunoassays, we design a new ELISA by modifying our previous methodology, comparing the response for **Z8P-IgG** between non-competitive ELISA and non-competitive fluorescent ELISA.

In both these ELISAs the standard curve was prepared by making serial dilutions of one known concentration of the primary, **Z8P-IgG**, across a range of concentrations (0-1500 ng/ml), while keeping every other parameter of the system constant. Each well was treated under the same conditions, and each measurement was done in quadruplicate. Both ELISA protocols took place using the exact same concentration for all species. Two measurements took place in parallel:

- **Non-competitive ELISA**: On a high-binding **transparent** microtiter plate (SI-7.4.1), where OD was measured as a final step
- **Non-competitive fluorescent ELISA:** On a high-binding **black** microtiter plate (SI 7.4.2), were fluorescence was measured as a final step

Note: the significant increase on the concentrations for our target and our primary is due to the fact that the fluorescence sensitivity is not high enough to work on the previously used range of concentrations (SI-7.3), thus we are forced to increase them to reasonably detect and quantify fluorescence.

The protocols area as follows:

7.4.1 Protocol of the non-competitive ELISA for the antibody conjugates Z8P-IgG

The protocol described in SI-7.2 was followed with the following modifications:

1) **Step 1 on SI-7.2 protocol:** Coat a 96 well transparent high-binding microtiter plates (flat-bottom, Nunc MaxiSorp™) with the target mouse IgG (**Capture antibody solution, SI-7.4.1.1**) by transferring 200 µL of the capture antibody solution into each well using a 200 µL multichannel electronic pipette and incubating for 4 h on a microplate shaker
2) **Step 9 in SI-7.2 protocol:** Add 200 µL of the ZIF-IgG conjugate solutions (**ZIF-IgG solutions in borate buffer, SI-7.4.1.2**) to the corresponding wells using a 200 µL pipette. Incubate for 18 hours.

7.4.1.1 Preparation of Capture antibody solution

1) Transfer 20 mL of borate buffer (see SI-2.2.1) to a long glass vial with a plastic cap using a 10 mL pipette
2) Transfer 5.0 µL of **mouse IgG** using a 5 µL pipette. Wash the tip by aspirating/dispensing the solution several times
3) Close the glass vial and homogenize the mixture d using a mechanical shaker

7.4.1.2 <u>Preparation of 4 stock solutions</u>

Prepare in total 4 stock solutions, each to a total volume of 1 mL. Stock solution **MS1** was prepared from the initial stock solution of **Z8P-IgG** (0.75 mg/mL); Stock solution **MS2** was prepared by dilution from **MS1**; Stock solution **MS3** was prepared by dilution from **MS2**; **MS4** was prepared by dilution from **MS3.**

MS1 was prepared as follows:

1) Transfer 1 mL of borate buffer inside a 1.5 mL Eppendorf tube
2) Remove 100 µL of the buffer using a 100 µL pipette
3) Transfer 100 µL of the original stock solution of the **Z8P-IgG** with a new tip on the micropipette. Wash the tip by aspirating/dispensing the solution several times
4) Seal the Eppendorf tube and homogenise the solution using a mechanical shaker

MS2 was prepared as follows:

1) Transfer 1 mL of borate buffer inside a new 1.5 mL Eppendorf tube
2) Remove 100 µL of borate buffer with a 100 µL pipette
3) Transfer 100 µL of the **MS1** stock solution with a new tip on the micropipette. Wash the tip by aspirating/dispensing the solution several times
4) Seal the Eppendorf tube and homogenise the solution using a mechanical shaker

MS3 was prepared as described for **MS2** but using **MS2** as the stock solution

MS4 was prepared as described for **MS2** but using **MS3** as the stock solution

<u>The concentration for the MS solutions will be:</u>

MS1 = 75 µg/mL;
MS2 = 7.5 µg/mL;
MS3 = 0.75 µg/mL;
MS4 = 0.075 µg/mL.

7.4.1.3 <u>Preparation of the dilution series for Z8P-IgG</u>

We prepare 23 new solutions from these four stock solutions (**MS1, MS2, MS3** and **MS4**) to varying concentrations for **Z8P IgG**.

The procedure **for each dilution series stock (number #)** is performed as follows:

1) Transfer 1 mL of borate buffer into a new Eppendorf tube.
2) Using the suitable micropipette, remove from the Eppendorf tube a volume of borate buffer equivalent to the corresponding cell on the column named “aliquot volume from MS stock solution” as listed on Table S16.
3) Using the same micropipette, transfer the aliquot volume corresponding to the exact same cell as on step 3 from Table S16, with a new tip on the micropipette. Wash the tip by aspirating/dispensing the solution several times
4) Seal the Eppendorf tube and homogenise the solution using a mechanical shaker

<u>**WARNING**</u>; In order to keep the antibodies stable during the whole process all solutions are kept on ice.

*Table S16 Table for the preparation of the ZIF−IgG solutions in borate buffer. The graphic representation of the results is in Fig. S21. The * represent the volume of buffer to be replaced by the stock solutions (MS1, MS2, MS3 and MS4).*

Dilution series stock<br>#	MS Stock solution used	aliquot volume from MS stock solution*<br>(µL)	Concentration in stock solution<br>(ng/mL)
1	MS1	20	1500
2	MS1	15	1125
3	MS1	10	750
4	MS1	8	600
5	MS1	6	450
6	MS1	4	300
7	MS1	2	150
8	MS2	10	75
9	MS2	8	60
10	MS2	6	45
11	MS2	4	30
12	MS2	2	15
13	MS3	10	7.5
14	MS3	9	6.75
15	MS3	6	4.5
16	MS3	4	3
17	MS3	2	1.5
18	MS4	10	0.75
19	MS4	8	0.6
20	MS4	6	0.45
21	MS4	4	0.03
22	MS4	2	0.015
23	MS4	0	0

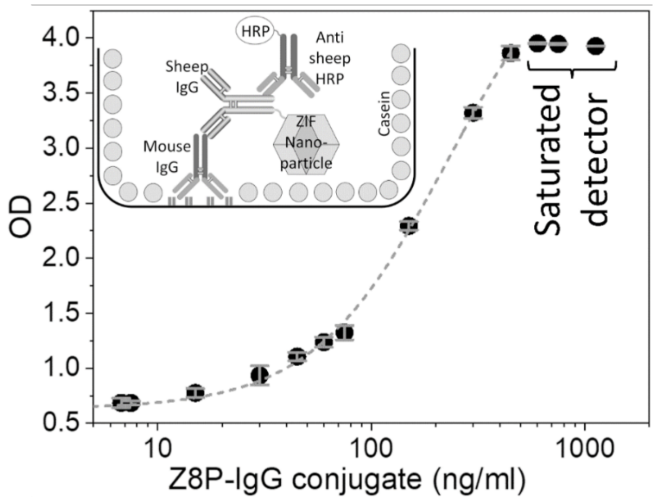

Fig. S21 Non-competitive indirect ELISA calibration curves for Z8P-IgG (anti-mouse, from sheep) using high concentrations (Table S16). The detector reaches saturation at concentrations above 350 ng/mL. The OD signal for the blank (coating antibody + blocking) is 0.72 ± 0.04.

7.4.2 Protocol of the non-competitive ELISA for the antibody conjugates Z8P-IgG via non-competitive fluorescence immunoassay

We developed a non-competitive indirect immunoassay for the assessment of the activity of the Z8P-IgG conjugate and its fluorescence capabilities.

The protocol described in SI-7.2 was followed with the following modifications:

1) **Step 1 in the SI-7.2 protocol:** This experiment was performed on a 96 well black high-binding plate for fluorescence measurements (flat-bottom, Nunc MaxiSorp™)
2) **Step 1 in the SI-7.2 protocol:** the target mouse IgG is prepared differently (**Capture antibody solution, SI-7.4.1.1**)
3) **Step 9 in SI-7.2 protocol:** Add 200 µL of the ZIF-IgG conjugate solutions (**ZIF-IgG solutions in borate buffer, SI-7.4.1.2**) to the corresponding wells using a 200 µL pipette. Incubate for 18 hours.
4) After the incubation in **step 11 in SI-7.2 protocol**, the fluorescence of each well was measured.
5) After the measurement, a three-cycle washing step takes place as described in **step 4 in SI-7.2 protocol** and each well was filled with 200 µL of borate buffer (see SI-2.2.1)
6) The fluorescence was then measured again
7) After this measurement, a three-cycle washing step took place as described in **step 4 in SI-7.2 protocol**
8) Each well was filled with 200 µL of citrate buffer (see SI-2.2.4) to disintegrate the ZIFs and liberate the fluorophores.
9) The plate was incubated in a plate shaker for 30 min
10) Fluorescence was then measured again

Table S17 Fluorescence results from the non-competitive fluorescent ELISA for Z8P-IgG. The graphic representation of the results is in Fig. S22.

Concentration (ng/ml)	Pre-Wash emission intensity (counts)	Post-Wash emission intensity (counts)	Post-wash + post citric acid emission intensity (counts)
1500	3793.50 ± 155.67	2115.67 ± 153.28	8887.25 ± 363.79
1125	3153.00 ± 141.77	1548.33 ± 23.12	5804.00 ± 233.98
750	2836.75 ± 146.33	1542.75 ± 259.12	5366.75 ± 423.67
600	2607.25 ± 116.64	1519.67 ± 180.78	3826.75 ± 357.38
450	2234.75 ± 73.25	1037.50 ± 46.51	3163.00 ± 62.23
300	2061.00 ± 19.88	969.75 ± 119.81	2740.50 ± 151.25
150	1833.75 ± 25.68	978.25 ± 99.68	2410.67 ± 135.61
75	1797.00 ± 96.09	950.24 ± 141.42	2332.00 ± 26.87
60	1719.00 ± 23.48	684.25 ± 126.27	2843.25 ± 344.27
45	1677.67 ± 13.65	612.67 ± 101.86	2577.00 ± 124.83
30	1677.33 ± 15.04	610.69 ± 105.51	2871.33 ± 602.85
15	1632.67 ± 9.19	609.26 ± 102.56	2457.00 ± 220.01
7.5	1623.50 ± 13.72		

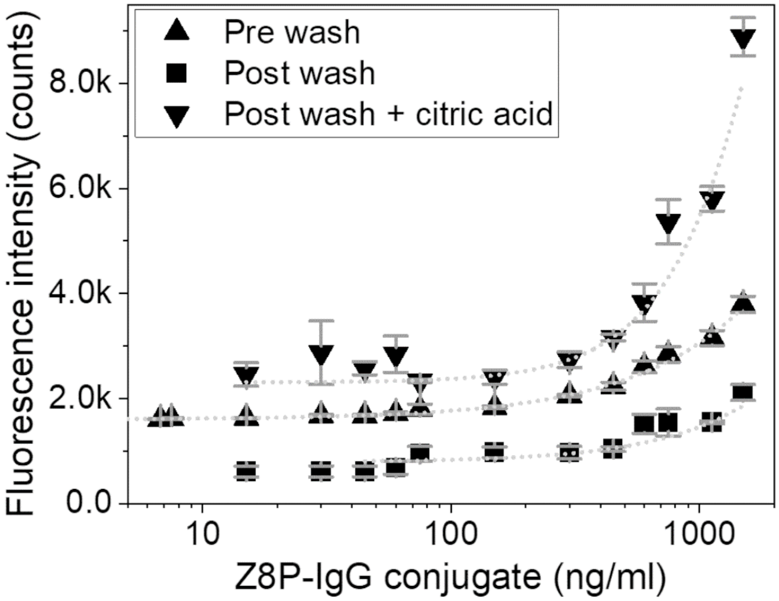

Fig. S22 Fluorescence emission for the non-competitive indirect assay employing Z8P-IgG obtained with an excitation wavelength of 335 nm. All the data points are blank-corrected (the mean intensity for the blank was subtracted from all points; the blanks' mean intensity was 168 ± 26 for pre wash fluorescence (blank = borate buffer), 143 ± 54 for post wash (blank = borate buffer) and 356 ± 5 after disintegration of the ZIF (by citric acid, blank = citric buffer).

Conclusion: Data suggests that the Z8P-IgG conjugate system can be used as a fluorescent bio label in immunoassays.

8 Studies on the decomposition of the ZIF conjugates

By determining the variation of the crystallite sizes over a specific window of time (5 days) keeping the conjugates in a borate dispersion (**0.75 mg ZIF-IgG/ml of Borate Buffer**) at 4°C and with stirring, it was possible to demonstrate that the crystallinity structure of the ZIFs after the conjugation reaction has not been heavily affected. The ZIF particle stability is dependent on the concentration of the dispersion in water.

8.1 PXRD measurements of the Z90-IgG and Z8P-IgG conjugates

To study how the crystallinity of the ZIFs (both **Z8P** and **ZIF-90**) could be degrading over time because of the action of the antibodies (antibody conjugated particles need to be immersed in aqueous buffer, which is known to slowly degrade ZIF-particles if not otherwise protected), we obtained X-ray diffractograms of a sample of the conjugates over time.

For each diffractogram, 400 µL aliquot (a total mass of 0.3 mg for each measurement) of the stock solution for each ZIF-IgG conjugate was freshly added to an Eppendorf tube, centrifuged (15000 rpm, 3 min) to reduce its volume to 50 µL by removing excess supernatant. The 50 µL dispersion was placed on a zero-background silicon monocrystalline (SIOV) sample holder. The measurement was started with the sample still wet. The following measurement conditions were used. 2θ 5-50°; step size 0.02°; integration time 6 sec.

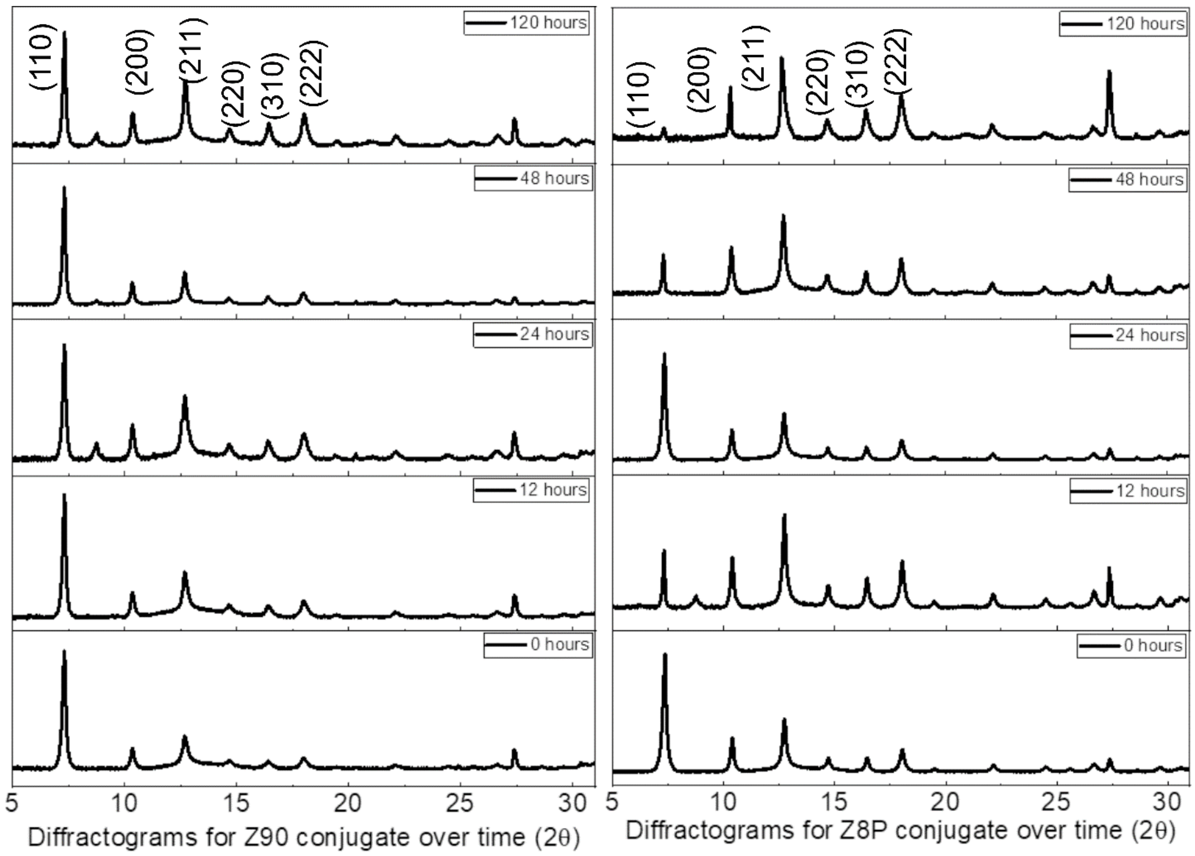

Fig. S23 Diffractograms taken at the times shown from the start of the stability experiments (0 to 120 hours) for both conjugate systems. Z90-IgG conjugate to the left and Z8P-IgG to the right. The background was subtracted from the diffractograms.

To asses that the crystallinity of the system was not severely damaged over time we measured the crystallite size for each system, applying the Scherrer equation to the first three reflections, (110), (200) and (211), for all diffractograms.

The Scherrer equation is a formula that relates the size of sub-micrometre crystallites in a solid to the broadening of a peak in a diffraction pattern and was used in the determination of size of crystals in the powder.[18] If the Scherrer value remains stable over time, this is an indication that there is no significant degradation of the ZIF particles in the conjugates.

$$\tau = \frac{K\lambda}{\beta cos\theta}$$

Equation S 1 . Scherrer equation for calculating the crystallite size, where "τ" is the mean size of the ordered (crystalline) domains, "K" is a dimensionless shape factor [we took 1], "λ" is the X-ray wavelength (1.50406 nm), "β" is the line broadening at half the maximum intensity (FWHM) in radians, "θ" the Bragg angle. We calculated all the FWHM for each diffraction peak after background subtraction and applying a Gaussian fitting model with Origin.

Table S18 FWHM for each of the first three diffractions for each conjugate (Z8P-IgG on top, Z90-IgG on bottom) for each of the degradation times studied. To calculate these values, we used the background subtracted diffractograms and we fitted a Gaussian model using Origin.

FWHM for each peak for Z8P−IgG			
Decomposition time (hours)	Diffraction [1,1,0]	Diffraction [2,0,0]	Diffraction [2,1,1]
0	0.222	0.192	0.247
12	0.119	0.192	0.241
24	0.220	0.198	0.243
48	0.121	0.192	0.248
120	0.157	0.153	0.247
FWHM for each peak for Z90−IgG			
Decomposition time (hours)	Diffraction [1,1,0]	Diffraction [2,0,0]	Diffraction [2,1,1]
0	0.201	0.209	0.291
12	0.192	0.204	0.288
24	0.195	0.210	0.316
48	0.187	0.190	0.258
120	0.174	0.197	0.251

Table S19 Values for the Scherrer crystallite sizes for all the diffractions chosen on the stability times chosen for our two ZIF−IgG systems. This data was illustrated in Fig. S24.

Scherrer crystallite size for Z8P−IgG			
Decomposition time (hours)	Diffraction [1,1,0]	Diffraction [2,0,0]	Diffraction [2,1,1]
0	13.602	15.773	12.264
12	25.394	15.724	12.585
24	13.732	15.225	12.476
48	24.890	15.748	12.195
120	19.187	19.716	12.264
**Scherrer crystallite size for Z90−IgG**			
Decomposition time (hours)	Diffraction [1,1,0]	Diffraction [2,0,0]	Diffraction [2,1,1]
0	15.019	14.487	10.405
12	15.707	14.828	10.517
24	15.466	14.390	9.590
48	16.145	15.940	11.754
120	17.313	15.317	12.049

We plot the crystallite sizes obtained of each diffraction peak versus decomposition time.

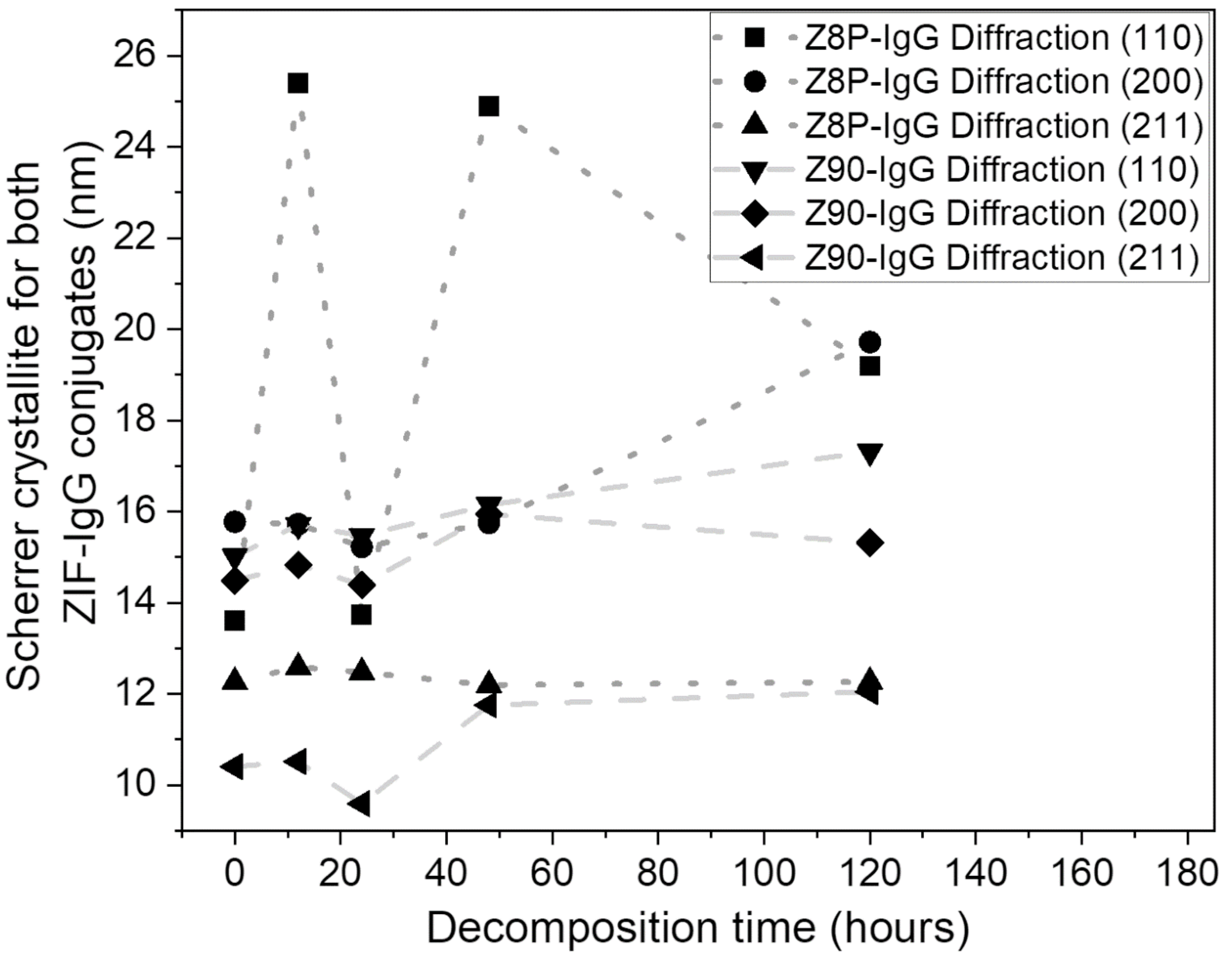

Fig. S24 Scherrer crystallite sizes for the first three reflections for the studied ZIF−IgG conjugates, Z8P−IgG and Z90−IgG. No marked changes in crystallite size were observed in the conjugated ZIFs under storage at 4°C for 120 hours using this crystallographic method. The pronounced variation on the (110) reflection for Z8P−IgG does not seem to be representative for the system.

A significant crystallinity degradation over time could not be observed in the studied time frame, since both systems have a similar range of crystallite sizes for all diffractions and over time. The pronounced variation on the diffraction [1,0,0] for Z8P-IgG does not seem to be representative of the system, and was probably caused by the sample not being completely immobilized on the sample holder (was deposited on a water dispersion and started measurement immediately), thus generating the disparity on the first 30 mins of measurement (corresponding to the first 8-9°, approximately).

8.2 Dynamic laser scattering (DLS) of the Z90-IgG conjugates

To study how the ZIF nanoparticles (both **ZIF-90** and **Z90-IgG)**) could be degrading over time on dispersion, DLS measurements were performed

8.2.1 Dispersant phase preparation

To measure on DLS it is critical that the dispersion has no dust particles present. To eliminate all non-desired particles from the dispersion, the dispersant (borate buffer) is double filtered before use. This is done my making the dispersant go through a double filter setup, using two different filters:

- Filter A: "*CHROMAFIL® Xtra H-PTFE-45/25*". A Hydrophobic PTFE/hydrophilic PTFE filter for polar and unpolar solvents, with a pore size of 0.45 μm and filter diameter of 25 mm.
- Filter B: "*WHATMAN® GD/X Sterile*". A PES filter media for polar and unipolar solvents, with a pore size of 0.2 μm and filter diameter of 25 mm.

8.2.2 DLS sample preparation and results

A dispersion on double filtered borate buffer of Z90-IgG with a concentration for approximately 0.1 mg/ml is prepared. Before the measurement on DLS is critical to sonicate the sample for at least 5 min, to break all the micro aggregates. This, however, must be performed carefully, since ultrasonicating can lead to fragmentations and exfoliation of the particles[19].

The systems behaviour on borate buffer shows two important points:

1- On the measured concentration, the particles tend to stay as microaggregates of around 1 μm, even though TEM shows the particles are 150-300 nm in diameter; this microaggregates cannot be broken down by ultrasonication alone, being necessary the introduction of stabilising agents, such as DMSO or other surfactants. This is likely a hydrophobicity issue, since ZIF-8 and -90 tends to stick together in very polar solvents due to the un-polarity of aged ZIF, which leads to forming colloidosomes.

2- Over time the microaggregates tend to increase in size and in size dispersity, hinting a tendency for the system to precipitate overtime. This phenomenon can be easily reverted by sonicating the sample for 3-5 minutes, unlike the microaggregates.

Table S20. Particle size distribution for Z90-IgG dispersed in borate buffer measured in DLS. Before the measurement of cycle 1, 5 min sonication for the sample took place. Cycle 2 happened immediately after cycle 1, with a 5 min sonication step in between cycles.

**Cycle 1**		
**Time from start**	**Particle size**	**St Dev**
(min)	(nm)	(nm)
0	1345	227.1
5	1773	373.3
10	1803	360.7
15	1944	391.5
20	2207	453
25	2196	453.1
**Cycle 2**		
**Time from start**	**Particle size**	**St Dev**
(min)	(nm)	(nm)
0	1340	233.1
5	1456	444.5
10	1606	460.2
15	1642	347.5
20	1694	361.5
25	1938	518.6

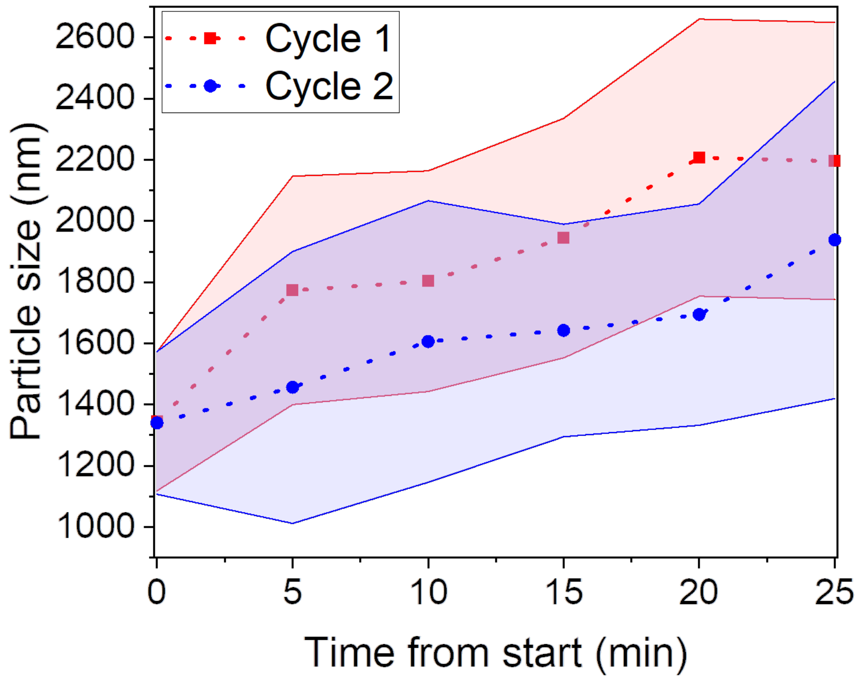

Fig. S25 Graphical representation of the results on Table S20. The coloured area around each line represents the standard deviation for the particle sizes as calculated by DLS (also in Table S20).

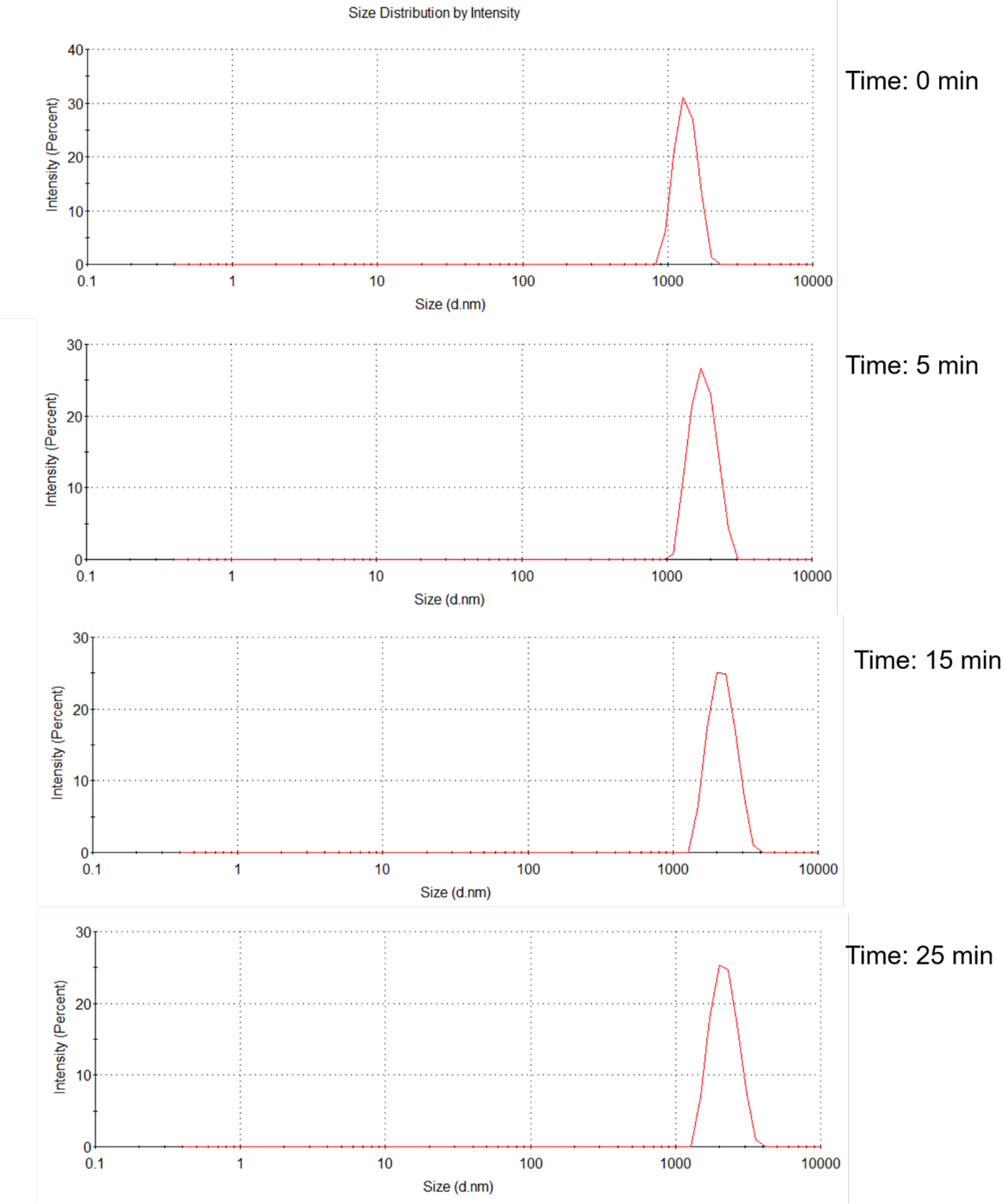

Fig. S26 Particle size distribution as a function of time determined by DLS for cycle 1. Same results are observed on cycle 2.

8.2.3 Nanoparticle tracking measurements for Z90-IgG.

To measure on NTA it is critical that the dispersion has no dust particles present. The big advantage over scattering methods NTA offers is that this is suitable for multi-disperse samples, as well as allowing small particles to be more visible than large particles of the same sample.

To eliminate all non-desired particles from the dispersion, the dispersant (borate buffer) is double filtered before use. This is done my making the dispersant go through a double filter setup, using two different filters:

- Filter A: "*CHROMAFIL® Xtra H-PTFE-45/25*". A Hydrophobic PTFE/hydrophilic PTFE filter for polar and unpolar solvents, with a pore size of 0.45 µm and filter diameter of 25 mm.
- Filter B: "*WHATMAN® GD/X Sterile*". A PES filter media for polar and unipolar solvents, with a pore size of 0.2 µm and filter diameter of 25 mm.

8.2.4 NTA sample preparation and results

A dispersion on double filtered borate buffer of Z90-IgG with a concentration of approximately 0.01 mg/ml is prepared. Before the measurement on NTA, it is critical to sonicate the sample for at least 5 min, to break all the micro aggregates.

In this technique is possible to see the nanoparticles individually on dispersion. A similar size dispersion can be seen (150-300 nm in diameter) as observed in TEM.

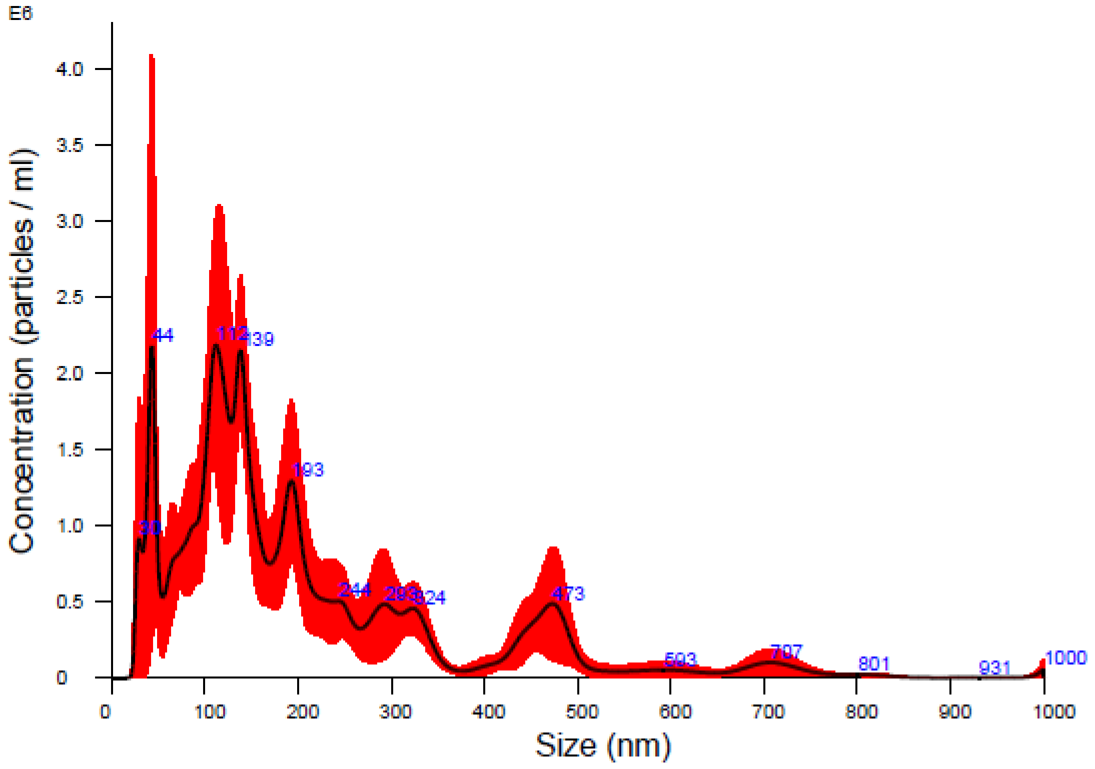

Fig. S27 Particle size distribution and standard deviation measured by NTA for the Z90-IgG dispersion in borate buffer.

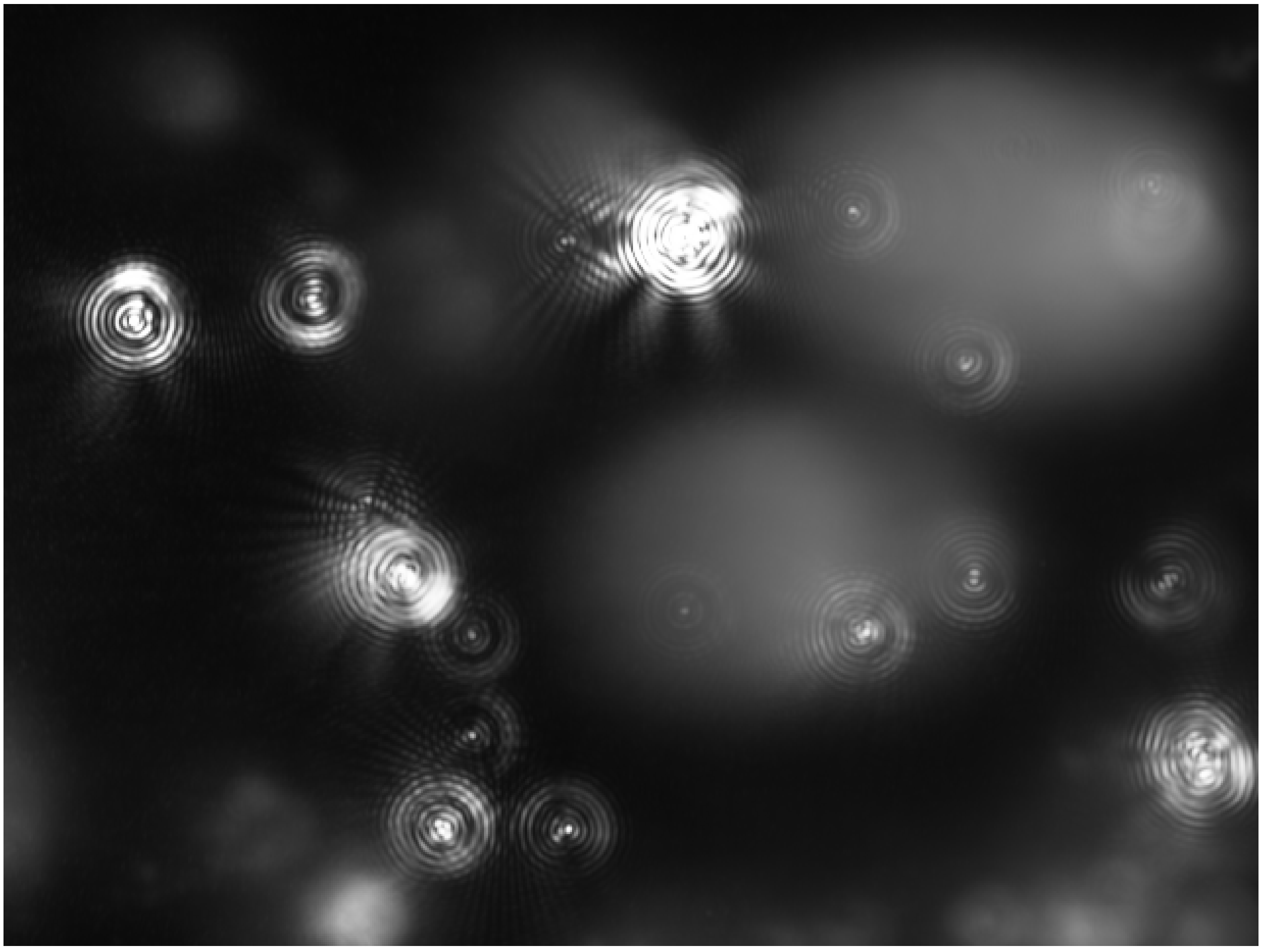

Fig. S28 Stationary image of the nanoparticle Z90-IgG dispersion seen through NTA. The rings around the particles are diffraction spheres, due to the crystallographic nature of the conjugate.

9 Studies on the ZIF-antibody bond

To study the nature of the bond between our ZIFs (ZIF-90 and Z8P) and the antibody (sheet anti-mouse IgG) we design a high-resolution mass spectroscopy methodology combined with TEM pictures.

9.1 TEM images of the ZIF-IgG conjugates

The protocol to characterize the particles via TEM is described in SI-4.4.2.

TEM images are consistent with the ZIF structure and particle size being retained upon formation of both ZIF conjugates. The antibodies appear as a rugged layer surrounding the ZIF particles.

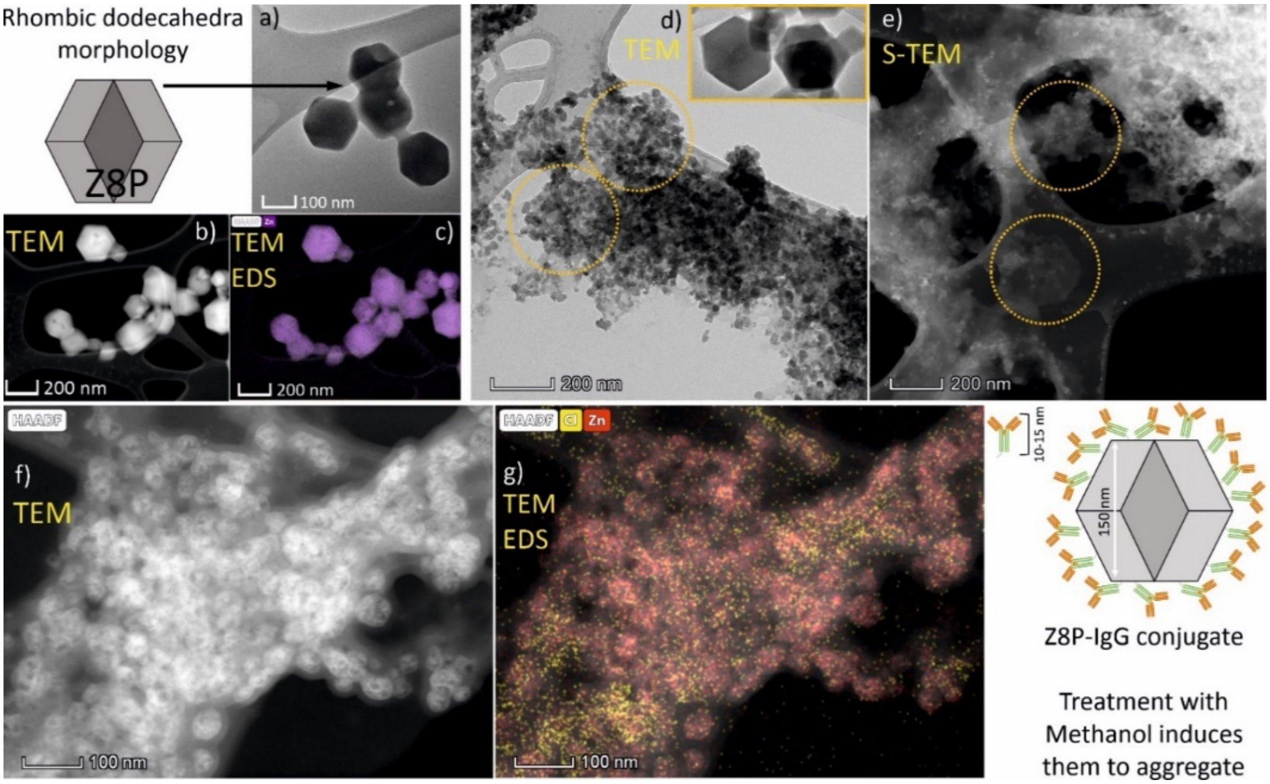

Fig. S29 TEM pictures for the ZIFs. A rhombic dodecahedra morphology can be observed for the Z8P-ZIF particles (pictures a-c), with some polydispersity in size, between 50 and 150 nm and averaging 100 nm. The pictures were complemented with EDS analysis (picture c), confirming that the signal for Zn was contained within the particles. For Z8P-IgG (pictures d-g) the particles were covered by a rugged layer of antibodies. The relative size of the whole system was in the same range as the one for the ZIF particles without the antibodies. The relative size of each entity was around 10 nm, with was consistent with the relative size of the antibodies themselves. S-TEM pictures were taken to detect the ZIF particles (picture e) and they were located within the yellow circles. EDS analysis complements the images (picture g). Most of the Zn signals were inside the rugged particles, which was consistent with the system description. There was also a not insignificant number of signals for zinc outside of the ruggedness, suggesting that after the conjugation procedure some of the ZIF have been damaged.

9.2 Study of the nature of the bond on the ZIF-IgG conjugates via HRMS

In order to confirm covalent conjugation through the imidazole carboxaldehyde moiety of the ZIF particles to a free amino group of the antibody, conjugates were digested (SI-9.2.1). Digestion results in the cleavage of most amide bonds in the protein and the potential release of L-lysine-bound Imidazolate-2-carboxaldehyde. The hydrolysate was then screened with liquid chromatography high-resolution mass spectrometry (LC-HRMS) (Fig. S30). After developing the methodology to detect the fragment (SI-9.2), it revealed a potential lysine-imidazole derivative at a retention time of 558 s (Fig. S31b). The compound was present in both ZIF-IgG conjugates and absent in all control experiments (individually digested IgG and ZIF without conjugation), thus supporting the notion of it being formed exclusively in ZIF−IgG conjugates.

9.2.1 Digestion of the conjugate

The protocol for digesting the samples was as follows:

To a distinct amount of sample (See Table S21 for more information) 1 mL of an aqueous solution of HCl 6N was added, the air was purged with the use of a Schlenk line with vacuum and Argon, and then heated to 107 °C for 24 h. After the digestion, the samples were cooled down and transferred to a 1.5 mL Eppendorf tube, and the solvent was removed using a SpeedVac.

Table S21. Amount for each species used in the digestion for subsequent mass spectrometric characterization.

Sample	Amount of sample
Sheep anti-mouse IgG	50 µL of a solution 2 mg/mL
ZIF-90	0.4 mg
Z8P	0.4 mg
ZIF-90−IgG conjugate	2 mg of conjugate
Z8P−IgG conjugate	2 mg of conjugate
Sheep IgG / i2ca	100 µg of IgG + 10 µg of i2ca in 0.5 mL of borate buffer
Sheep IgG / i2ca	100 µg of IgG + 1 µg of i2ca in 0.5 mL of borate buffer

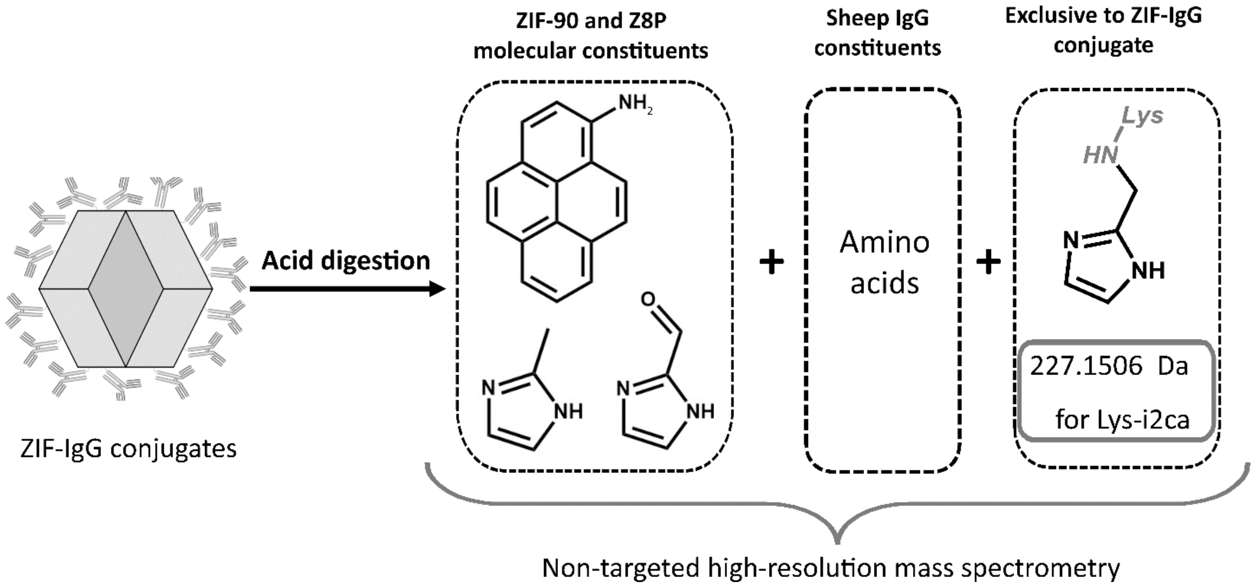

Fig. S30 Molecular constituents as expected to be released by the acidic hydrolysis of ZIF−IgG conjugates. The highlighted Lys amino acid imidazole derivatives (m/z 227.1506 Da) accepted to be exclusive obtained from ZIF−IgG conjugates.

9.2.2 High-resolution mass spectrometry

First, the efficiency of this hydrolysis was tested using pure antibody instead of the conjugate. Extracted ion chromatograms (EICs) of protonated and deprotonated molecular peaks ($[M+H]^+$, $[M-H]^-$) acquired in both positive and negative electrospray ionisation modes suggest the presence of 17 of the 20 proteinogenic amino acids in the sample (Fig. S32), indicating efficient protein breakdown. Next, ZIF-IgG conjugates were hydrolysed and subjected to LC-HRMS. Chromatograms of hydrolysed ZIF-IgG conjugates contained approximately 140 chromatographically deconvoluted compounds ("features") in the mass range of 50 to 600 Da (Fig. S31a). This shows extensive hydrolytic breakdown of conjugates into smaller molecules. In these chromatograms, ion traces of possible amino acid imidazole derivatives were checked using a target list of theoretical precursor ions (Table S22).

This approach revealed a potential lysine imidazole derivative at a retention time of 558 s (Fig. S31b). The compound was present in both ZIF/IgG conjugates but in none of the control measurements (IgG alone, ZIFs alone), supporting the notion that it was formed exclusively in ZIF/IgG conjugates.

Structural evidence for the compound could be obtained from both isotope pattern and MS/MS spectrum. First, isotopologue intensities matched calculated ones within instrument-limited error margins (<2%; Table S23). Second, all major MS/MS fragments could be assigned substructures that explained the putative molecular structure very consistently (Fig. S31c).

Taken together, while only co-elution of a chemical standard or NMR could give definite structural evidence, covalent bonding of an Imidazolate-carboxaldehyde moiety to an amino acid residue of the antibody was well supported by the present results.

For mass spectrometric analysis (SI-3.9), hydrolysed samples were reconstituted in acetonitrile/methanol (75:25) containing 0.2% formic acid and injected into a 1290 UHPLC system (Agilent, Germany) coupled to a 6600 TripleTOF mass spectrometer (SCIEX, Germany). Samples were separated on a 2.1 mm × 75 mm × 1.8 µm BEH Amide column (Waters, Germany) using a hydrophilic liquid interaction chromatography (HILIC) mode.

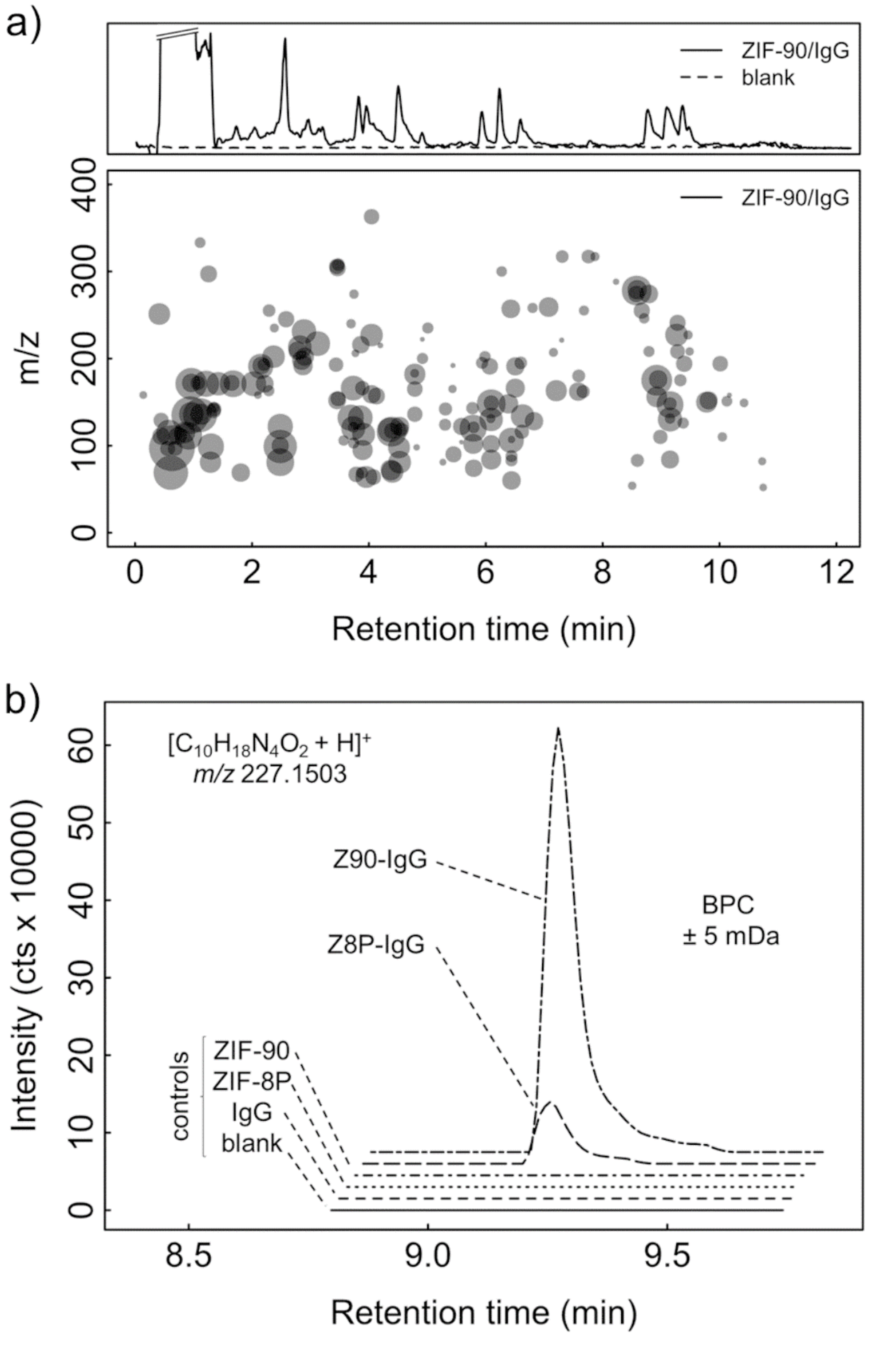
a)
ZIF-90/IgG
blank
ZIF-90/IgG
m/z
Retention time (min)
b)
[$C_{10}H_{18}N_4O_2$ + H]$^+$
m/z 227.1503
Z90-IgG
Z8P-IgG
BPC
± 5 mDa
controls
ZIF-90
ZIF-8P
IgG
blank
Intensity (cts x 10000)
Retention time (min)

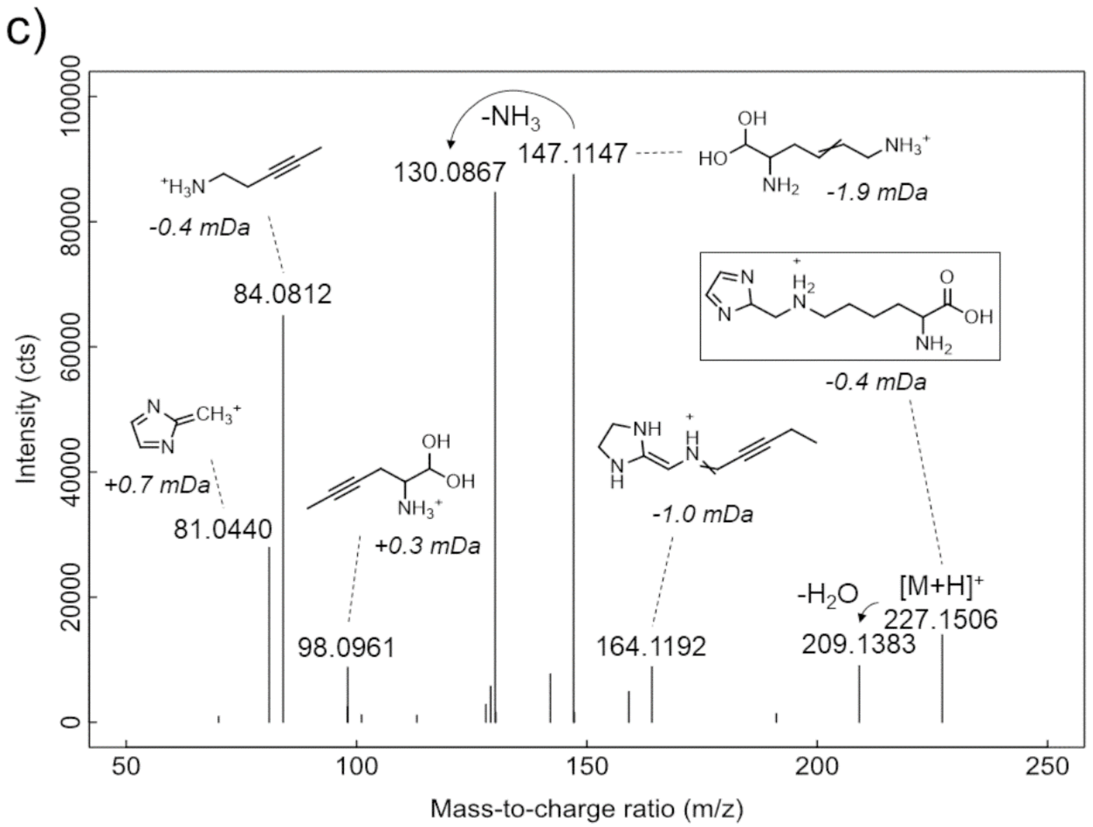

Fig. S31 Mass spectrometric characterization of the assumed covalent bond in ZIF-IgG conjugates. (a) Nontargeted (HILIC-)LC-ESI-HRMS of hydrolysis products; representative total ion chromatogram (TIC) and ion map, respectively, demonstrating sample complexity. (b) Extraction of a putative lysine imidazolate derivative ($C_{10}H_{18}N_4O_2$, m/z 227.1503). Ion traces were detected in measurements of both ZIF conjugates but not in those of controls. (c) MS/MS spectrum of putative lysine derivative (precursor m/z 227.1503, isolation window 1 Da). MS/MS fragments were assigned most likely molecular structures based on competitive fragmentation modelling (CFM-ID). Deviations of theoretical from measured masses were indicated. Minor fragment annotation left out for clarity.

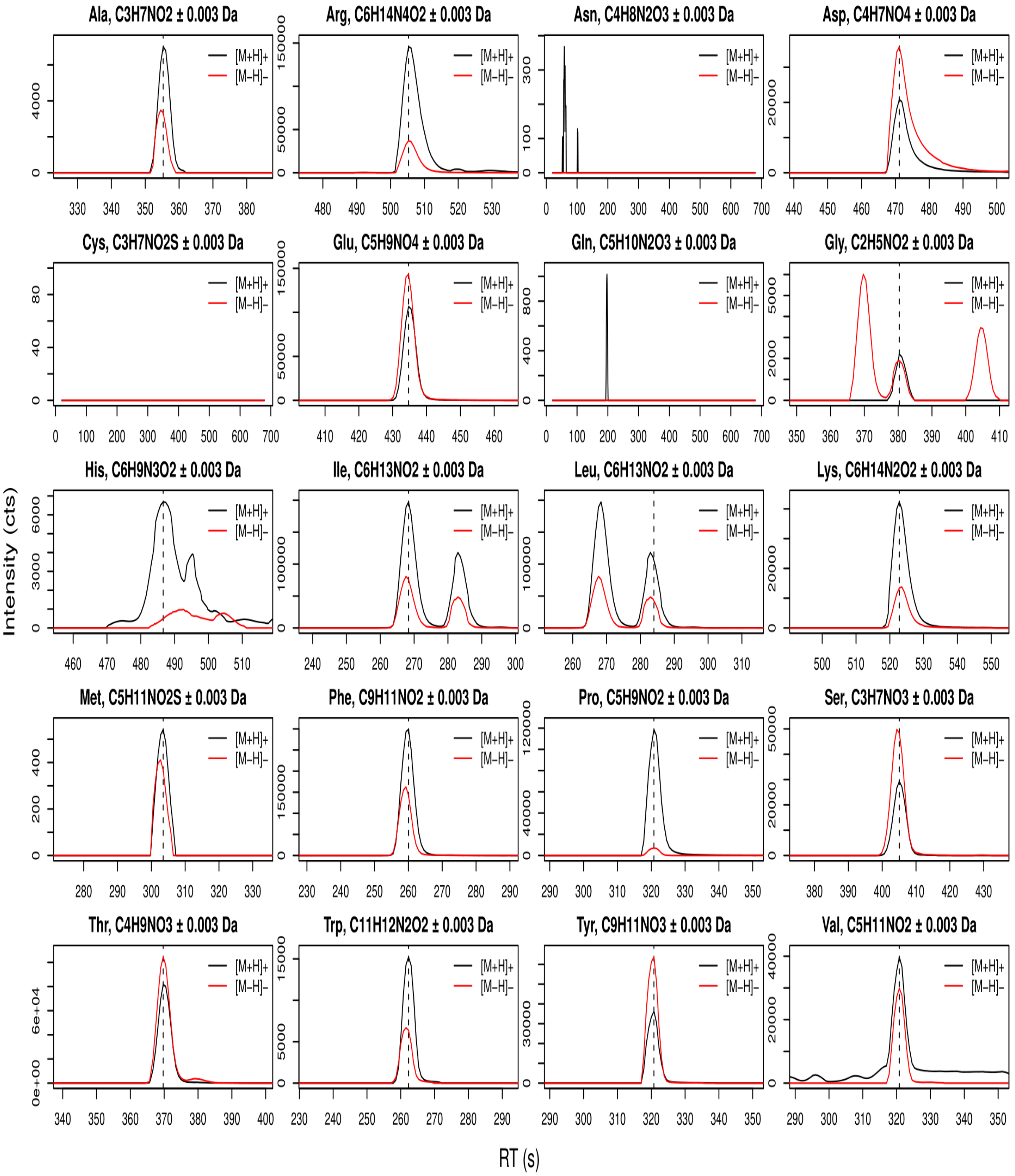

Fig. S32 *Extracted ion chromatograms for the 20 proteinogenic amino acids released from acidic hydrolysis of 0.01 mg IgG (anti-mouse, from sheep). Extracted ions ($[M+H]^+$, $[M-H]^-$) and mass windows are indicated.*

Table S22 LC-MS target list for amino acids and putative amino acid imidazolate derivatives

Name	SMILES	Formula	Neutral mass	$[M+H]^+$	$[M-H]^-$
Ala	CC(C(=O)O)N	C3H7NO2	89.0477	90.0550	88.0404
Ala imidazole derivative 1	CC(C(=O)O)NCC1N=CC=N1	C7H11N3O2	169.0851	170.0924	168.0779
Arg	C(CC(C(=O)O)N)CN=C(N)N	C6H14N4O2	174.1117	175.1190	173.1044
Arg imidazole derivative 1	C(CC(C(=O)O)NCC1N=CC=N1)CN=C(N)N	C10H18N6O2	254.1491	255.1564	253.1418
Arg imidazole derivative 2	C(CC(C(=O)O)N)CN=C(N)NCC1N=CC=N1	C10H18N6O2	254.1491	255.1564	253.1418
Asn	C(C(C(=O)O)N)C(=O)N	C4H8N2O3	132.0535	133.0608	131.0462
Asn imidazole derivative 1	C(C(C(=O)O)NCC1N=CC=N1)C(=O)N	C8H12N4O3	212.0909	213.0982	211.0837
Asn imidazole derivative 2	C(C(C(=O)O)N)C(=O)NCC1N=CC=N1	C8H12N4O3	212.0909	213.0982	211.0837
Asp	C(C(C(=O)O)N)C(=O)O	C4H7NO4	133.0375	134.0448	132.0302
Asp imidazole derivative 1	C(C(C(=O)O)NCC1N=CC=N1) C(=O)O	C8H11N3O4	213.0750	214.0822	212.0677
Cys	C(C(C(=O)O)N)S	C3H7NO2S	121.0197	122.0270	120.0125
Cys imidazole derivative 1	C(C(C(=O)O)NCC1N=CC=N1)S	C7H11N3O2S	201.0572	202.0645	200.0499
Glu	C(CC(=O)O)C(C(=O)O)N	C5H9NO4	147.0532	148.0604	146.0459
Glu imidazole derivative 1	C(CC(=O)O)C(C(=O)O) NCC1N=CC=N1	C9H13N3O4	227.0906	228.0979	226.0833
Gln	C(CC(=O)N)C(C(=O)O)N	C5H10N2O3	146.0691	147.0764	145.0619
Gln imidazole derivative 1	C(CC(=O)NCC1N=CC=N1)C (C(=O)O)N	C9H14N4O3	226.1066	227.1139	225.0993
Gln imidazole derivative 2	C(CC(=O)N)C(C(=O)O)NCC1N=CC=N1	C9H14N4O3	226.1066	227.1139	225.0993
Gly	C(C(=O)O)N	C2H5NO2	75.0320	76.0393	74.0248
Gly imidazole derivative 1	C(C(=O)O)NCC1N=CC=N1	C6H9N3O2	155.0695	156.0768	154.0622

Name	SMILES	Formula	Neutral mass	[M+H]+	[M-H]-
His	C1=C(NC=N1)CC(C(=O)O)N	C6H9N3O2	155.0695	156.0768	154.0622
His imidazole derivative 1	C1=C(NC=N1)CC(C(=O)O)NCC1N=CC=N1	C10H13N5O2	235.1069	236.1142	234.0996
Ile	CCC(C)C(C(=O)O)N	C6H13NO2	131.0946	132.1019	130.0874
Ile imidazole derivative 1	CCC(C)C(C(=O)O)NCC1N=CC=N1	C10H17N3O2	211.1321	212.1394	210.1248
Leu	CC(C)CC(C(=O)O)N	C6H13NO2	131.0946	132.1019	130.0874
Leu imidazole derivative 1	CC(C)CC(C(=O)O)NCC1N=CC=N1	C10H17N3O2	211.1321	212.1394	210.1248
Lys	C(CCN)CC(C(=O)O)N	C6H14N2O2	146.1055	147.1128	145.0983
Lys imidazole derivative 1	C(CCNCC1N=CC=N1)CC(C(=O)O)N	C10H18N4O2	226.1430	227.1503	225.1357
Lys imidazole derivative 2	C(CCN)CC(C(=O)O)NCC1N=CC=N1	C10H18N4O2	226.1430	227.1503	225.1357
Met	CSCCC(C(=O)O)N	C5H11NO2S	149.0510	150.0583	148.0438
Met imidazole derivative 1	CSCCC(C(=O)O)NCC1N=CC=N1	C9H15N3O2S	229.0885	230.0958	228.0812
Phe	C1=CC=C(C=C1)CC(C(=O)O)N	C9H11NO2	165.0790	166.0863	164.0717
Phe imidazole derivative 1	C1=CC=C(C=C1)CC(C(=O)O)NCC1N=CC=N1	C13H15N3O2	245.1164	246.1237	244.1092
Pro	C1CC(NC1)C(=O)O	C5H9NO2	115.0633	116.0706	114.0561
Ser	C(C(C(=O)O)N)O	C3H7NO3	105.0426	106.0499	104.0353
Ser imidazole derivative 1	C(C(C(=O)O)NCC1N=CC=N1)O	C7H11N3O3	185.0800	186.0873	184.0728
Thr	CC(C(C(=O)O)N)O	C4H9NO3	119.0582	120.0655	118.0510
Thr imidazole derivative 1	CC(C(C(=O)O)NCC1N=CC=N1)O	C8H13N3O3	199.0957	200.1030	198.0884
Trp	C1=CC=C2C(=C1)C(=CN2)CC(C(=O)O)N	C11H12N2O2	204.0899	205.0972	203.0826
Trp imidazole derivative 1	C1=CC=C2C(=C1)C(=CN2)CC(C(=O)O)NCC1N=CC=N1	C15H16N4O2	284.1273	285.1346	283.1200
Tyr	C1=CC(=CC=C1CC(C(=O)O)N)O	C9H11NO3	181.0739	182.0812	180.0666

Name	SMILES	Formula	Neutral mass	[M+H]+	[M-H]-
Tyr imidazole derivative 1	C1=CC(=CC=C1CC(C(=O)O) NCC1N=CC=N1)O	C13H15N3O3	261.1113	262.1186	260.1041
Val	CC(C)C(C(=O)O)N	C5H11NO2	117.0790	118.0863	116.0717
Val imidazole derivative 1	CC(C)C(C(=O)O)NCC1N=CC=N1	C9H15N3O2	197.1164	198.1237	196.1092

Table S23. Mass accuracy and isotope pattern analysis for putative lysine imidazole derivative at 558 s (m/z 227.1500)

Isotope	m/z	theoretical m/z	Error (mDa)	Intensity (%)	Theoretical intensity (%)	Difference (%)
M	227.1500	227.1503	0.3	100	100	0
M+1	228.1533	228.1530	0.3	14.26	12.56	-1.69
M+2	229.1567	229.1552	1.5	1.31	1.14	-0.17

10 Studies on the mobility of the ZIF-antibody conjugate

The mobility of the ZIF conjugates was important for their application in bioanalytical assays. The feasibility of our ZIF-IgG system as a mobile conjugate on a solid matrix for lateral flow assays is next tested. PVDF matrix is chosen from the candidates available; tests on nitrocellulose were performed but proved to be an unsuitable matrix given the strong interactions between the nitrocellulose and the conjugates.

In order to facilitate the proof of concept for the Z8P platform as a bio label, we create a new conjugate system, **Z8P-IgG HRP**, by changing the antibody conjugated to the ZIFs from **sheep-anti-mouse IgG** to **HRP labelled donkey anti-sheep IgG**

For details on the antibodies see SI-1.6.

10.1 Conjugation reaction of Z8P particles to IgG−HRP

10.1.1.1 *Process for the conjugation of ZIF-90 and Z8P with IgG-HRP*

1) 1.5 mg of **Z8P** was carefully weighed on a 5-figure balance and transferred to a small glass vial with a plastic cap.
2) 1.5 mL of a borate buffer solution (see SI-2.2.1) was added, measured with a 2.5 mL pipette
3) After sealing the glass vial with the cap, it was sonicated for 15 min
4) 50 µL of an **anti-sheep IgG-HRP solution** (HRP conjugated to donkey anti-sheep IgG was added without any previous purification step using a 100 µL pipette to this dispersion
5) A magnetic stirrer bar was added, the glass vial closed, and the mixture stirred for 15 min at room temperature
6) 50 µL of a commercially prepared aqueous solution of **Na[BH$_3$(CN)]** (5 M) in 1 M NaOH was added using a 200 µL pipette and allowed to react for an additional 4 hours at room temperature.

Warning: Exercise extreme care when handling Cyanoborohydride. Always wear gloves, glasses and work in a properly enclosed fume hood.

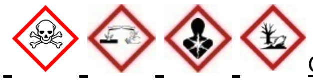
GHS05, GHS06, GHS08, GHS09

10.1.1.2 *Protocol for washing the freshly prepared Z8P-IgG HRP*

Next step was designed to remove any anti-sheep IgG HRP not covalently bonded to Z8P. The washing protocol of this conjugation reaction is as follows:

1) Transfer the 1.5 mL suspension to a 2 mL Eppendorf tube with the help of a 1 mL pipette and centrifuge it at 15000 rpm for 5 min.
2) Carefully remove the supernatant taking care not to disturb the solid residue and transfer it to a common container labelled as “Wash 1”. This wash is reserved for later use, in case the ELISA experiment fails suggesting unsuccessful binding between the

IgG and the ZIF. "Wash 1" will help to investigate if the free IgG in the wash was active or inactive. "Wash 1" is disposed of, after successful completion Elisa experiments

Warning: The very first supernatant of "Wash 1" contains cyanide derivatives. Extreme care must be exercised when dealing and disposing with "Wash 1".

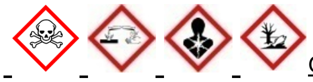
GHS05, GHS06, GHS08, GHS09

3) Add 1 mL of borate buffer to the Eppendorf tube to wash the solid residue.
4) Sonicate the suspension for 2 min to ensure full dispersion of the solid particles
5) Centrifuge the dispersion at 15000 rpm for 5 min
6) Remove carefully the supernatant avoiding contact with the solid residue and transfer it to a separately container labelled as ""Wash 2"
7) Add 1 mL of TRIS buffer (see SI-2.2.3) to the residue
8) Redisperse the solid residue by vigorous agitation, and centrifuge at 15000 rpm for 5 min
9) Carefully remove this TRIS buffer supernatant and discard it
10) Repeat **steps 7-9** three more times to wash out any IgG not properly conjugated to the ZIF
11) Perform **steps 7-9** two additional times, this time with borate buffer
12) Disperse the properly washed solid residue containing the ZIF conjugate in 2 mL of borate buffer. This is the **ZIF-IgG HRP stock solution** (0.75 mg/ml) used in all the ELISA analysis

Conclusion: The amount of washing steps was developed so that only the covalently bound antibody remains on the ZIF particles.

10.2 Mobility studies of ZIF conjugates on a PVDF membrane

The mobility was studied on the commercial membrane TransBlot® Turbo™ Midi-size PVDF Membrane (Bio-Rad, Cat. # 10026933).

10.2.1.1 Protocol for the PVDF mobility tests of Z8P-IgG

1) The **activation of the PVDF strip** is done by submerging the membrane in pure ethanol for 10 sec
2) Remove the excess ethanol by rinsing it in deionized water for 3 sec
3) Allow the strip to dry at open atmosphere
4) Place 1 µL of a **sheep anti-mouse IgG** (2 mg/ml) solution on the immobilization point using a 5 µL micropipette operating in reversed mode
5) Allow the PVDF strip with the immobilized sheep IgG dry
6) Submerge the strip into a **casein** solution in PBS (1mg/mL, see SI-7.2.2) for ten seconds to block all active sites on the strip
7) Allow the strip to dry at room temperature.

8) Dispense 10-30 µL of the **Z8P-IgG HRP** dispersion in borate buffer (0.75 mg/mL) on the elution starting point using a reverse micropipette.
9) Allow to dry
10) Attach an adsorption pad on the opposite side of the strip in relation to the elution starting point.
11) Elute the Z8P-IgG HRP using borate buffer until the deposited Z8P-IgG HRP reaches the adsorption pad (yellow colouring from the Z8P)
12) Allow dry.

The Z8P-IgG HRP can be seen by the naked eye on the capture zone and on the adsorption pad (yellow colouring), indicating antibody binding.

13) **TMB** solution is used to reveal the location of the HRP-labelled antibodies. This will trigger an intense blue coloration within minutes wherever IgG HRP labelled are present, with by extension shows were the ZIF-IgG HRP conjugates are present.
14) Cut out the relevant area of the strip and digest it in 200 µL of 1 M H_2SO_4 for 15 min using a mechanical shaker and a desk centrifuge to aid the process.
15) Pipetted the digestion solution into a 96-well black microtiter plate.
16) Measure the fluorescence of the solution measured

Note: TMB and PVDF samples were also prepared as negative controls.

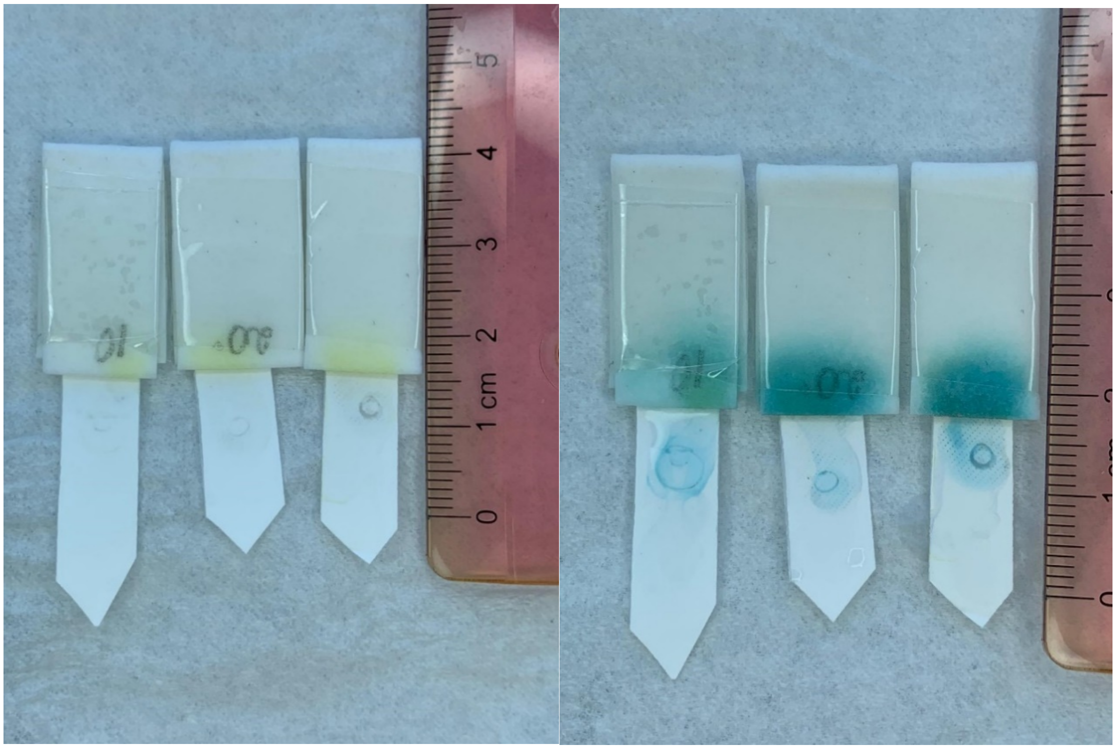

Fig. S33 Outcome of the Lateral Flow Immunoassay built upon Z8P-IgG. Left: The Z8P-IgG can be seen by the naked eye in the capture zone and on the adsorption pad, indicating antibody binding and surplus antibody, respectively. Right: TMB solution was used to reveal the location of the HRP-labelled antibodies. In both pictures all amounts for all compounds of the system were kept constant (1 µL of a sheep IgG solution in PBS (2 mg/mL); casein solution 1 mg/mL in PBS) and the initial volume of Z8P-IgG dispersion increases from left to right from 10, 20 to 30 µL of the Z8P-IgG in borate buffer (1 mg/mL).

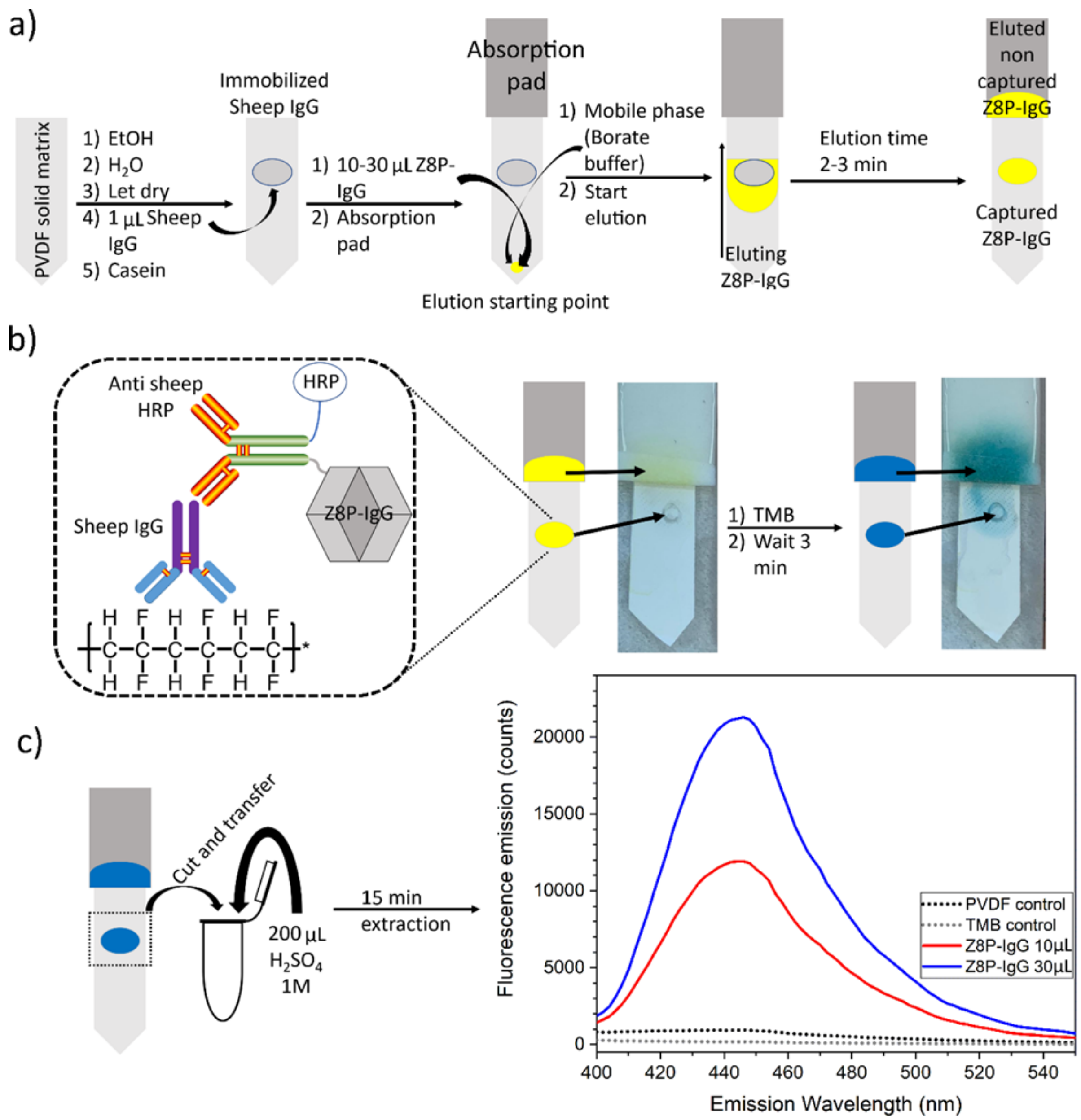

Fig. S34 Experimental set-up to demonstrate the aptitude of Z8P-IgG for the bioanalytical application lateral flow immunoassay (LFIA). a) Assay. The protocol begins by activating the PVDF membrane through submersion in pure ethanol followed by rinsing in deionized water. After air drying, a solution containing sheep IgG as target is placed in a central part of the strip, the later capture zone. The PVDF strip with the immobilized sheep IgG is then submerged in a casein solution to block all remaining active sites on the strip, and afterwards allowed to dry. A varying volume of the Z8P-IgG-HRP dispersion is pipetted on the elution starting point and allowed to dry. Then the absorption pad is attached to the strip. Subsequently, the Z8P-IgG-HRP is eluted by adding enough of a mobile phase (borate buffer) to the strip that the phase's front-line flows across the capture zone into the absorption pad. This is followed by drying the strip in air. b) Revelation of mobility of the composite and retention of binding specificity of the antibody: The capturing of Z8P-IgG by the target (sheep antibody) in the capture zone is visible by the colour of the ZIF particles. For stronger evidence that the labelled antibody is binding selectively to the immobilized target, the HRP enzyme linked to Z8P-IgG is revealed via its reaction with the added substrate TMB. c) Proof of intact ZIF label. The area to which the antibody has bound is cut out and digested in H_2SO_4 to decompose the ZIF and liberate the pyrene. The supernatant is transferred to a black microwell and fluorescence of the solution measured. The emission spectra show that fluorophore is released in amounts related to the volume of conjugate employed (PVDF and TMB samples that were not run through the assay serve as negative control).

11 References

[1] L. Oberleitner, U. Dahmen-Levison, L.-A. Garbe, R. J. Schneider, *Analytical Methods* **2016**, *8*, 6883-6894.
[2] R. Tautenhahn, C. Boettcher, S. Neumann, *BMC Bioinformatics* **2008**, *9*, 504.
[3] F. Allen, A. Pon, M. Wilson, R. Greiner, D. Wishart, *Nucleic acids research* **2014**, *42*, W94-W99.
[4] C. Wu, Q. Liu, R. Chen, J. Liu, H. Zhang, R. Li, K. Takahashi, P. Liu, J. Wang, *ACS applied materials & interfaces* **2017**, *9*, 11106-11115.
[5] H. Kaur, G. C. Mohanta, V. Gupta, D. Kukkar, S. Tyagi, *Journal of Drug Delivery Science and Technology* **2017**, *41*, 106-112.
[6] W. Morris, C. J. Doonan, H. Furukawa, R. Banerjee, O. M. Yaghi, *Journal of the American Chemical Society* **2008**, *130*, 12626-12627.
[7] J. Cravillon, R. Nayuk, S. Springer, A. Feldhoff, K. Huber, M. Wiebcke, *Chemistry of Materials* **2011**, *23*, 2130-2141.
[8] N. Lugg, G. Kothleitner, N. Shibata, Y. Ikuhara, *Ultramicroscopy* **2015**, *151*, 150-159.
[9] J. Troyano, A. Carné-Sánchez, C. Avci, I. Imaz, D. Maspoch, *Chem Soc Rev* **2019**, *48*, 5534-5546.
[10] E. Cunha, M. F. Proença, M. G. Pereira, M. J. Fernandes, R. J. Young, K. Strutyński, M. Melle-Franco, M. Gonzalez-Debs, P. E. Lopes, M. D. C. Paiva, *Nanomaterials (Basel)* **2018**, *8*.
[11] A. Chapartegui-Arias, J. A. Villajos, A. Myxa, S. Beyer, J. Falkenhagen, R. J. Schneider, F. Emmerling, *ACS Omega* **2019**, *4*, 17090-17097.
[12] J. R. Lakowicz, in *Principles of fluorescence spectroscopy*, Springer, **1983**, pp. 257-301.
[13] C. Albrecht, **2008**, *390*, 1223-1224.
[14] K. E. Al Ani, A. E. Ramadhan, S. Khanfar, *Materials Sciences and Applications* **2017**, *8*, 1027.
[15] F. Adams, S. Aime, L. A. Andersson, I. Ando, D. M. Andrenyak, D. L. Andrews, L. Andrews, S. R. Anthony, T. G. Appleton, C. M. Arroyo, **2010**.
[16] C. Hofmann, A. Duerkop, A. J. Baeumner, *Angewandte Chemie International Edition* **2019**, *58*, 12840-12860.
[17] J. Cravillon, R. Nayuk, S. Springer, A. Feldhoff, K. Huber, M. Wiebcke, *Chem Mater* **2011**, *23*, 2130-2141.
[18] P. H. Ho, F. Salles, F. Di Renzo, P. Trens, *Inorganica Chimica Acta* **2020**, *500*, 119229.
[19] S. Beyer, C. Prinz, R. Schürmann, I. Feldmann, A. Zimathies, A. M. Blocki, I. Bald, R. J. Schneider, F. Emmerling, *ChemistrySelect* **2016**, *1*, 5905-5908.
